# From Mechanoelectric Conversion to Tissue Regeneration: Translational Progress in Piezoelectric Materials

**DOI:** 10.1002/adma.202417564

**Published:** 2025-05-28

**Authors:** Xinyu Wang, Sílvio Terra Stefanello, Victor Shahin, Yun Qian

**Affiliations:** ^1^ National Center for Orthopaedics, Department of Orthopaedics Shanghai Sixth People's Hospital Affiliated to Shanghai Jiao Tong University School of Medicine 200233 Shanghai China; ^2^ Institute of Physiology II University of Münster Robert‐Koch‐Str. 27b 48149 Münster Germany

**Keywords:** advanced materials for translational medicine, biosensors, piezoelectricity, regenerative medicine, tissue engineering

## Abstract

Piezoelectric materials, capable of converting mechanical stimuli into electrical signals, have emerged as promising tools in regenerative medicine due to their potential to stimulate tissue repair. Despite a surge in research on piezoelectric biomaterials, systematic insights to direct their translational optimization remain limited. This review addresses the current landscape by bridging fundamental principles with clinical potential. The biomimetic basis of piezoelectricity, key molecular pathways involved in the synergy between mechanical and electrical stimulation for enhanced tissue regeneration, and critical considerations for material optimization, structural design, and biosafety is discussed. More importantly, the current status and translational quagmire of mechanisms and applications in recent years are explored. A mechanism‐driven strategy is proposed for the therapeutic application of piezoelectric biomaterials for tissue repair and identify future directions for accelerated clinical applications.

## Graphical abstract

This review highlights recent progress in piezoelectric materials for regenerative medicine, emphasizing their ability to convert mechanical stimuli into bioelectric signals that promote tissue repair. Key discussions cover the intrinsic piezoelectric properties of biological tissues, co‐stimulation cellular mechanisms for tissue regeneration, and optimized structural designs aligned with specific tissue demands for translational applications.

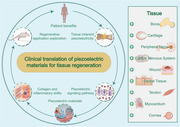

## 1 Introduction

The goal of regenerative medicine is to restore tissues and organs to their normal function and structure using biomedical principles and engineering tools. It is a cutting‐edge field in the 21st century. While mesenchymal stem cell (MSC) therapies like Ryoncil^[^ ^1^ ^]^ and Alofisel^[^ ^2^ ^]^ represent current FDA‐approved clinical strategies, significant hurdles persist. These include safety concerns, such as infections and fever observed with Ryoncil,^[^ ^3^ ^]^ and challenges with demonstrating consistent long‐term efficacy, which contributed to Alofisel's market withdrawal.^[^ ^2^ ^]^ In order to provide better clinically translatable regenerative medicine therapies, finding more reliable and controllable alternatives is necessary.

Fundamental to tissue development and repair are endogenous bioelectrical and mechanical signals. In the human body, bioelectricity is present in all tissues and closely relates to cellular functions such as proliferation, differentiation, migration, and apoptosis, which are important in embryonic development, regenerative medicine, and cancer.^[^ ^4^ ^]^ Reprogramming of bioelectrical signals can help develop novel approaches to tissue regeneration. Mechanical signals are also associated with embryonic development and cell fate. Force transduction between the cytoskeleton and extracellular matrix can direct various cellular behaviors, especially cell migration, which is closely related to tissue regeneration.^[^ ^5^ ^]^ Therefore, modulating these bioelectrical and mechanical cues represents a powerful strategy to guide tissue regeneration more effectively.

The unique advantage of piezoelectric materials over existing means is the property for fine modulation of these bioelectrical and mechanical cues, through the customization of materials and the programming of mechanical stimuli, which means that they have good controllability and clinical translational potential. Piezoelectric materials generate a voltage under stress, thus bridging mechanics and electricity, and are thus innate electromechanical synergistic agents. Compared to direct electrical stimulation, piezoelectric materials do not require an external power source or invasive setup, providing a more adaptable and natural electrical environment. Compared to unstable cellular therapies, piezoelectric materials allow for better control of tissue regeneration by optimizing material parameters. Moreover, many biological tissue components like bone, skin, proteins, and amino acids have piezoelectric properties.^[^ ^6^ ^]^ Therefore, piezoelectric materials also possess unique biomimetic properties, which can promote tissue regeneration by mimicking natural electromechanical properties and adapting to dynamic movement. Developing biomimetic piezoelectric materials is significantly important to mimic the tissue electromechanical microenvironment, reprogram pathological signals, and enable tissue regeneration.

Many clinical healing dilemmas are expected to be solved by the introduction of piezoelectric materials. For patients with non‐healing fractures, piezoelectric materials such as barium titanate or hydroxyapatite composites can be used in implants or scaffolds.^[^ ^7^ ^]^ These materials generate electrical signals in response to mechanical stress from weight‐bearing activities, stimulating osteoblast differentiation and bone formation. In cases of peripheral nerve injury, piezoelectric polymers such as polyvinylidene fluoride (PVDF) can be made into nerve conduits.^[^ ^8^ ^]^ These conduits provide both structural support and electrical stimulation, thereby improving functional recovery by promoting nerve regeneration. For chronic wounds such as diabetic or pressure ulcers, piezoelectric dressings made from materials such as PVDF or zinc oxide can convert mechanical forces, such as wound contraction or external pressure, into electrical signals that promote cell proliferation and migration, speeding up the healing process and reducing the risk of infection.^[^ ^9^ ^]^ For patients with heart failure due to myocardial infarction, piezoelectric heart patches can be used to stimulate the growth and differentiation of cardiomyocytes, helping to regenerate damaged myocardium and improve heart function.^[^ ^10^ ^]^ Theoretically, piezoelectric materials have a clinical translational potential that cannot be ignored for repair and regeneration in the context of all types of tissue injuries.

It is worth noting that even though piezoelectric materials have great potential, there are no practical clinical trial cases of their use for tissue regeneration. This means that piezoelectric materials face considerable clinical translational challenges, including, but not limited to, ambiguous perceptions of the complex microenvironment of tissue regeneration and the role of piezoelectric materials.^[^ ^11^ ^]^ Therefore, it is important to comprehensively review the research lineage of piezoelectric materials in the context of tissue regeneration. While there are existing reviews on piezoelectric materials in tissue regeneration, they often focus on material properties alone and lack an updated perspective on the translational potential.^[^ ^12^ ^]^ This review aims to pull existing research out of the mire and push it toward the path of clinical translation by providing a comprehensive introduction to piezoelectric materials, including piezoelectric principles, piezoelectric components present in living organisms, and piezoelectric materials for tissue regeneration. Critically, it summarizes the potential pathways for synergistic electromechanical signals and their translational prospect in tissue regeneration. The review also addresses the optimization of processes and topologies, discusses biosafety considerations necessary for clinical translation, and presents thorough descriptions of the latest application cases.

## 2 Biological Basis of the Piezoelectric Effect: Biomimetic Cornerstone

### 2.1 Inherent Piezoelectricity of Biological Tissues

The piezoelectric effect is present not only in inorganic materials but also in organic components in various parts of living organisms. The piezoelectric properties of human tissues have been shown to be involved in regulating essential life activities such as bone remodeling.^[^ ^13^ ^]^ Therefore, to gain a comprehensive understanding of the piezoelectric characteristics inherent in biological tissues, thorough investigation and exploration are essential, which can help researchers link the piezoelectric concept with the biomedical field, to combine macroscopic tissue deformation with microscopic bioelectric signals, to establish the theoretical basis of piezoelectricity in the biomedical perspective, and to provide inspirations for novel diagnostic and biomimetic therapeutic strategies. The inherent piezoelectricity of biological tissues is summarized in **Table** **1**.

**Table 1:** The inherent piezoelectricity of biological tissues.

Species	Tissue	Piezoelectricity	Refs.
Human	Tibia	d_33_ 7.66–8.72 pC N^−1^	[14]
Rat	Tail tendon	Macroscopic d_33_ 0.09 pm V^−1^, d_31_ 0.07 pm V^−1^, d_14_ −2.7 pm V^−1^, d_15_ 1.5 pm V^−1^ Nanoscale d_33_ 0.9 pm V^−1^, d_31_ −4.8 pm V^−1^, d_14_ −12 pm V^−1^, d_15_ 6.2 pm V^−1^	[15]
Porcine	Articular cartilage	Larger voltage signals in deeper layers	[16]
Bovine	Intervertebral disk	d_33_: Annulus fibrosus 0.073 pC N^−1^, nucleus pulposus 0.034 pC N^−1^	[17]
Human	Corneal	d_?_: diagonal (2250 pC N^−1^) > vertical (600 pC N^−1^) > horizontal (200 pC N^−1^)	[18]
Human	Sclera	d_?_: circumferential 7–23 pC N^−1^, anterior‐posterior 6–8 pC N^−1^ Circumferential: middle (23 pC N^−1^) > posterior (17 pC N^−1^) >anterior (7 pC N^−1^)	[19]
Sperm whale	Dental tissues	d_?_: Dentin and cementum, 0.027‐0.028 pC N^−1^	[20]
Human	Dental tissues	d_33_: Dentin 0.30 pC N^−1^, enamel 0.51 pC N^−1^	[21]
Human	Live human epidermis	d_33_: 0.02–0.19 pC N^−1^	[22]
Human	Skin tissues	d_14_: dermis 0.05–0.1 pC N^−1^, epidermis 0.01–0.03 pC N^−1^, corneum 0.1–0.2 pC N^−1^	[23]
Guinea pigs	Outer hair cells	50‐µm‐long lateral membrane piezoelectric coefficient 20 µC N^−1^	[24]

Fukada and Yasuda first demonstrated the piezoelectric properties of bone tissue in 1957, using dried bone plates from the bovine and human femurs.^[^ ^25^ ^]^ They measured the shear piezoelectric coefficients of human and bovine bones, and attributed them to the movement of collagen fibers under shear stress. Bassett et al. observed a correlation between the magnitude of the stress and the amplitude of the potential, as well as a correlation between the polarity and the direction of deformation in specimens. Freshly prepared bone tissue specimens produced a potential amplitude increase compared to bone tissue that had been subjected to freezing/thawing/air‐drying operations.^[^ ^26^ ^]^ These phenomena suggest the existence of an alternative pathway for pressure‐generated potentials in bone tissue in living organisms, which was demonstrated by Anderson et al. and termed “streaming potentials”.^[^ ^27^ ^]^ They assessed the piezoelectric coefficients of both dry and wet bone, discovering that the wet bone exhibited substantially higher values. They suggest that only a few of the wet bone collagen retains its piezoelectric properties because it is covered with hydroxyapatite (HA) and has not hydrated. The main mechanical‐electrical signals originate from the streaming potential of the fluid in the pores. However, Halperin et al. stated that there was no significant difference in the piezoelectric response of wet and dry bone samples measured by piezoresponse Force Microscopy (PFM).^[^ ^14^ ^]^

Fukada and Yasuda also measured the shear piezoelectric coefficients, d_14_, of bovine and horse Achilles tendon in 1964, finding them about ten times that of the bone tissue, which may be due to the consistent hydrogen bonding orientation of collagen in tendons along the long axis of collagen fibers.^[^ ^28^ ^]^ A recent nanoscale study showed that the piezoelectric response was observed in rat tail tendon collagen from 10% to 70% relative humidity by transverse PFM.^[^ ^29^ ^]^ Denning et al. studied rat tail tendons by PFM and found regions of opposite polarity in the aligned collagen fibers. The longitudinal piezoelectric coefficient d_33_ of a single collagen fibril was an order of magnitude higher than that of the macroscopic tendon.^[^ ^15^ ^]^

The potential generated by strain in biological cartilage tissues is thought to result from a combination of piezoelectricity, flow potentials, Donnan potentials, and diffusion potentials.^[^ ^30^ ^]^ The earliest piezoelectricity of cartilage should be traced back to the study of Bassett et al.^[^ ^31^ ^]^ Different types of hydrated cartilage exhibited varying degrees of response voltage, with the lowest in tracheal cartilage and the highest in articular cartilage and meniscus. A recent study on porcine articular cartilage showed that cartilage's piezoelectric response depends on the collagen fibers’ deformation after being subjected to shear forces.^[^ ^16^ ^]^ The porcine articular cartilage has a transverse, irregular, and longitudinal arrangement of collagen fibers from superficial to deep layers so that the voltage signal is most pronounced in the deeper layers. Poillot et al. measured the piezoelectric properties of the annulus fibrosus and nucleus pulposus in the intervertebral discs by using PFM and found that the median d_33_ of the annulus fibrosus was significantly greater than that of the nucleus pulposus, probably due to the presence of a collagen network in the annulus fibrosus.^[^ ^17^ ^]^

The cornea and sclera, as collagen‐rich tissues in the human body, are also subject to piezoelectricity observation. It has been found that the cornea has an anisotropic piezoelectric response.^[^ ^18^ ^]^ The average piezoelectric coefficient of human corneal samples cut along the diagonal was measured to be the largest, followed by those cut vertically, and the smallest for those cut horizontally. Ghosh et al. investigated the piezoelectric properties of human and bovine scleral tissues.^[^ ^19^ ^]^ The piezoelectric coefficients of samples cut circumferentially around the pupil were larger than those cut in an anterior‐posterior direction. In the circumferential samples, the piezoelectric coefficients were ordered as follows: middle > posterior > anterior. The reason for the large piezoelectric coefficient difference between the cornea and sclera deserves to be explored in more studies and may be related to various factors such as tissue composition, tissue structure, sampling location, stress‐strain direction, and measurement method.

Teeth mainly consist of dentin, enamel, cementum, and pulp. The first three, all piezoelectric teeth components, comprise inorganic components (HA, the majority) and organic components (collagen type I, a small fraction).^[^ ^20^, ^21^, ^32^ ^]^ Marino et al. first reported on the piezoelectricity of dentin in 1989 and found that the piezoelectric constants of sperm whale dentin and cementum were essentially the same, which was one‐tenth of that of adult tibia.^[^ ^20^ ^]^ Reyes‐Gasga et al. studied the piezoelectricity of adult enamel and dentin by PFM.^[^ ^21^ ^]^

Shamos et al. demonstrated for the first time that dry human skin and stratum corneum are piezoelectric.^[^ ^33^ ^]^ They conjectured that this effect originated from collagen and elastin. Athenstaedt et al. determined the piezoelectric coefficient d_33_ of the epidermis of the hairless region on the back of the finger of a living human being and found that the piezoelectric coefficient of the dry skin was 20–60% lower than that of the living skin.^[^ ^22^ ^]^ Derossi et al. further determined the piezoelectricity of the skin of human breasts, thighs, prepuce, and plantar calluses.^[^ ^23^ ^]^ They separated the dermis, epidermis, and stratum corneum and vacuum dehydrated them. The maximum shear piezoelectric coefficient d_max_ was measured, with the stratum corneum having the most pronounced piezoelectric properties. They concluded that the piezoelectricity of the skin originates mainly from the shear piezoelectricity of the collagen network in the dermis, and from the a‐helical keratin‐like fibrils in the epidermis and stratum corneum.

Besides the tissues mentioned above, cochlear outer hair cells, blood vessels, and lungs have also received attention. Brownell summarized cochlear outer hair cells’ structural and piezoelectric properties (OHCs).^[^ ^34^ ^]^ The piezoelectricity of OHCs from theoretical modeling speculations because of their ability to convert membrane potentials into cellular deformations and electromechanical‐electrical transitions. Dong et al. measured the piezoelectric coefficient to be 20 µC N^−1^ for OHCs.^[^ ^24^ ^]^ Fukada et al. reported piezoelectric properties in porcine aorta and vena cava, tracheal and intestinal tissues of dogs, and ligaments of bovine legs.^[^ ^35^ ^]^ They suggested that the main contributors were collagen and elastin. The piezoelectricity of elastin has also been demonstrated in rat lung tissue.^[^ ^36^ ^]^ Furthermore, cell membranes and DNA appear to have piezoelectric properties.^[^ ^37^ ^]^ The piezoelectric properties of amino acids, peptides, and proteins are described in detail in the review by Kim et al.^[^ ^6^ ^]^ and will not be repeated here.

### 2.2 Utilizing the Inherent Piezoelectricity of Tissues: Where Do We Stand?

While the inherent piezoelectricity of biological tissues, such as bone and tendons, has been well‐established and linked to critical processes like mechanotransduction and tissue remodeling, its direct utilization in the design of piezoelectric biomaterials remains in its early stages. Current research predominantly focuses on developing materials that replicate the piezoelectric properties observed in natural tissues, thereby providing biomimetic electrical stimulation to enhance regeneration. A notable example is the development of natural collagen scaffolds that preserve the complete tertiary structure of collagen, retaining its intrinsic piezoelectricity. Studies have demonstrated that such scaffolds, when subjected to mechanical stimulation significantly promote bone regeneration by generating piezoelectric signals akin to those in native tissues.^[^ ^38^ ^]^ This approach leverages the tissue's inherent properties by mimicking them in the biomaterial, rather than directly interacting with or modulating the tissue's own piezoelectric signals.

In contrast, as we will detail in the following sections on various materials and studies, most contemporary strategies, such as those involving PVDF‐based materials, emphasize the material's ability to generate electrical signals under physiological loads or mechanical stimulation to simulate the bioelectric environment, without explicitly integrating the tissue's dynamic piezoelectric response. The natural collagen scaffold approach stands out as a rare instance where the material's piezoelectricity is derived from preserving a structure mirroring that of native tissues, suggesting a step toward utilizing inherent piezoelectricity. Developing materials that not only mimic but also harness the tissue's inherent piezoelectricity could unlock significant advantages. Such materials could establish a dynamic electromechanical synergy, potentially amplifying the tissue's natural regenerative signals and creating an adaptive feedback loop where the material responds to the tissue's real‐time physiological demands. This could result in more efficient and targeted regeneration, closely aligned with the body's natural processes. Moreover, by matching the material's electromechanical properties to those of the native tissue, these materials could enhance tissue integration, reducing inflammation or rejection risks. Clinically, this approach could decrease dependence on external devices for stimulation, simplifying treatments and improving patient comfort, especially for conditions like bone defects requiring sustained regeneration support. However, the potential for designing materials that can synergize with or amplify the tissue's own piezoelectric signals remains largely unexplored. Advancing this frontier could lead to more sophisticated biomimetic strategies, potentially offering superior therapeutic outcomes by harmonizing the electromechanical behaviors of both the material and the host tissue.

## 3 The Role of Piezoelectric Signals in Tissue Regeneration

Piezoelectric signals consist of both electrical and mechanical components. Indeed, it is not new that electrical and mechanical stimulation modulate cellular activity separately to promote tissue repair.^[^ ^39^ ^]^ Discussing the two jointly is a worthwhile endeavor that contributes to a more comprehensive piezoelectric‐tissue regeneration perspective. As early as 1997, Spadaro made an interesting reflection on the mechanism of mechano‐electrical interactions during bone remodeling by suggesting that electrical stimulation (ES) needs to be accompanied by other stimuli that include mechanical stimulation (MS) and that MS has a higher priority than ES.^[^ ^40^ ^]^

Crucially, the hallmark of piezoelectric stimulation lies in its inherent coupling of mechanical stress and electrical charge generation at the source. Unlike conventional ES, which typically applies an exogenous electrical field (AC or DC) independent of the local mechanical state, piezoelectricity generates electrical signals as a direct consequence of mechanical deformation within the material or tissue itself. This results in a unique microenvironment where cells experience simultaneous mechanical forces and the resultant, highly localized, and often transient electrical cues. The magnitude, polarity, and temporal dynamics of these piezoelectric signals are intrinsically linked to the characteristics of the applied mechanical load.

This inherent mechano‐electrical conversion distinguishes piezoelectric phenomena significantly. The piezoelectric effect delivers a combined stimulus package—mechanical deformation plus the resulting electrical potential—potentially engaging cellular mechanotransduction and electrotransduction pathways concurrently and synergistically in a manner fundamentally different from applying either stimulus modality in isolation or sequentially. Understanding this integrated signaling is key to deciphering the full potential of piezoelectricity in regenerative contexts.

Therefore, the mechanisms outlined in this section specifically address potential pathways activated or modulated by this distinctive electromechanical coupling inherent to piezoelectric signals, rather than solely by independent mechanical or electrical stimuli. Admittedly, the mechanism is so complex that we can only paint a general picture.

**Figure 1:**
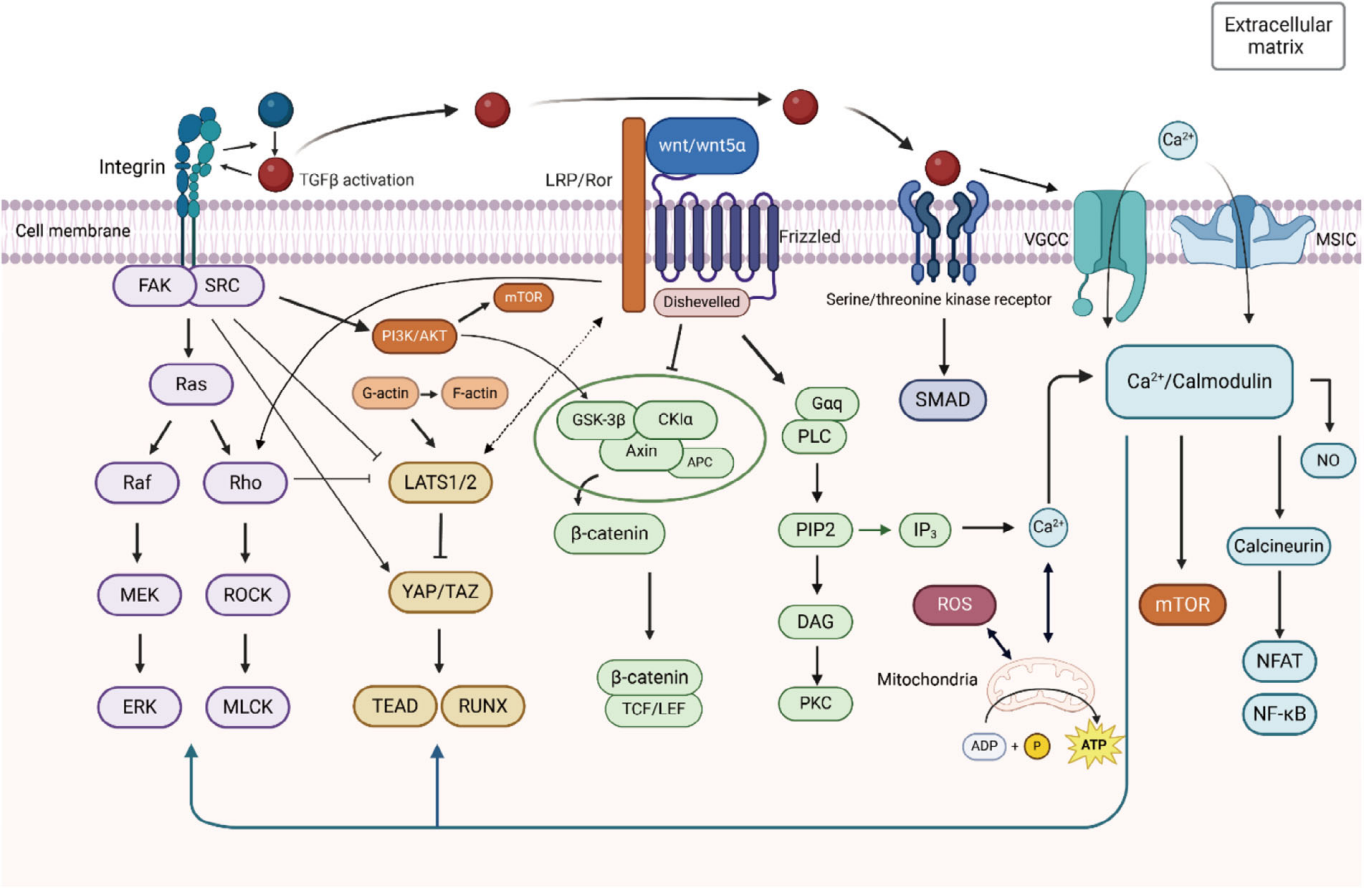
Possible synergistic signaling pathways for mechanical and electrical signals in tissue regeneration. There are seven possible pathways: integrin/FAK/Ras, Hippo (YAP/TAZ), wnt/β‐catenin, TGF‐β, ion channels, calcium signaling, and piezocatalytic effects. Complex cross‐talk exists between pathways, especially between the integrin pathway and the Hippo pathway. The calcium signaling pathway plays a predominantly downstream role and also has a regulatory role on upstream pathways. Created in BioRender. (2024) https://BioRender.com/a48v522.

Here, using the knowledge base available at this stage, we try to establish a preliminary electromechanical signaling network oriented to tissue regeneration by marking the locations of possible encounters between mechanical and electrical signals from seven directions: intracellular Ca^2+^ levels, ion channels, ECM (extracellular matrix)/integrins/FAK (focal adhesion kinase), the YAP (Yes‐associated protein)/TAZ (transcriptional co‐activator with PDZ‐binding motif) pathway, the Wnt pathway, the TGF‐β (transforming growth factor‐β)/SMAD (small mother against decapentaplegic) pathway, and the piezoelectric catalytic effect. Admittedly, the electromechanical tissue regeneration network is a rather large and complex system, and precise crosstalk mechanisms remain to be further elucidated. Possible synergistic signaling pathways for mechanical and electrical signals are shown in **Figure** **1**.

### 3.1 Intracellular Ca^2+^ Levels

Both electrical and MS increase intracellular Ca^2+^, which triggers a series of downstream cellular responses related to tissue regeneration.^[^ ^39^, ^41^ ^]^ There are two pathways by which intracellular Ca^2+^ increases: one is through membrane ion channels that mediate the inward flow of extracellular Ca^2+^, and the other is by stimulating the release of Ca^2+^ from the endoplasmic reticulum within the cell.^[^ ^42^ ^]^ The increase in intracellular Ca^2+^ further mediates tissue repair‐related signaling through a variety of possible pathways, including calmodulin (CaM), myosin, and immune responses.^[^ ^43^ ^]^ CaM promotes cell proliferation mainly by promoting cell cycle progression and activating CaM‐dependent protein kinases.^[^ ^44^ ^]^ In addition, Ca^2+^ and CaM crosstalk with a variety of pathways, such as the Ras/Raf/MEK/ERK, SRC‐family protein tyrosine kinase (SFK), mechanistic target of rapamycin (mTOR), transforming growth factor‐beta (TGF‐β), and Hippo signaling pathway, thereby promoting cell proliferation, differentiation, migration, regulating cell autophagy and maintaining cellular homeostasis.^[^ ^45^ ^]^ A detailed summary of Ca^2+^‐promoted tissue repair‐related pathways was provided by Ghilardi et al.^[^ ^43b^ ^]^ Briefly, calcium concentration waves can promote fibroblast contraction, migration, and wound introgression through activation of the calcium‐dependent CaM‐myosin light chain kinase (MLCK) complex or through activation of the proline‐rich kinase‐2 (Pyk2)/Ras homolog gene family member A (RhoA) pathway.^[^ ^43^, ^46^ ^]^ It can also promote fibroblast proliferation and activation by activating transcription factor pathways such as the nuclear factor of activated T cells (NFAT) and nuclear factor kappa‐B (NF‐κB) via calmodulin phosphatase.^[^ ^47^ ^]^ In addition, immune cells can be recruited via DUOX (an NADPH oxidase), or low levels of nitric oxide (NO) can be produced via nitric oxide synthase (NOS), which in turn promotes tissue repair.^[^ ^48^ ^]^ However, the immune response has both protective and destructive effects and should be viewed dialectically.^[^ ^49^ ^]^

### 3.2 Ion Channels

ES and MS can promote extracellular Ca^2+^ inward flow and increase intracellular Ca^2+^ concentration through specific ion channels, thereby triggering a series of downstream cellular responses related to tissue regeneration.

#### 3.2.1 Voltage‐Gated Calcium Channels

ES can introduce extracellular Ca^2+^ through activation of voltage‐gated calcium channels (VGCC), as well as release Ca^2+^ from the endoplasmic reticulum within the cell through activation of the phospholipase C (PLC)/inositol‐1,4,5‐trisphosphate (IP3) pathway, which in turn triggers the above‐mentioned repair responses.^[^ ^50^ ^]^ Evidence for relevant electro‐calcium regulation is not lacking, but the details of the selection of different electro‐calcium pathways by ES deserve further exploration. For example, Brighton et al. found that the mode of ES was related to the pathway, with capacitive coupling first activating VGCC in MC3T3‐E1 osteoblasts, whereas inductive coupling and combined electromagnetic fields first released intracellular calcium.^[^ ^50b^ ^]^ Xu et al. concluded that bovine patellar chondrocytes under a capacitively coupled electric field (60 kHz, 20 mV/cm) mainly activated VGCC to promote extracellular Ca^2+^ inward flow and downstream pathways rather than PLC/IP3‐mediated intracellular calcium pathway.^[^ ^50c^ ^]^ Khatib et al. concluded that high‐intensity exogenous ES (10 V/cm) activated VGCC in fetal osteoblasts, whereas physiological‐intensity ES (1–2 V cm^−1^) activated the PLC/IP3 pathway and stretch‐activated cation channels (SACC).^[^ ^50f^ ^]^ Notably, upon physiological ES of osteoblasts, they found that the maximum Ca^2+^ fluorescence intensity under blocked SACC treatment was very close to that placed in Ca^2+^‐free Hanks equilibrium salt solution, suggesting that physiological ES appears to activate SACC, possibly bridging the gap between ES and mechanosensitive ion channels (MSICs). Clearly, more studies are needed to elucidate possible differences in the electro‐calcium pathway at different ES intensities and modalities to reach a consensus.

#### 3.2.2 PIEZO 1/2

MS can promote extracellular Ca^2+^ endocytosis via MSICs, the most talked about being the PIEZO family. As the first class of non‐selective mechanically gated cation channels identified in mammals, the PIEZO family is a crucial mechano‐electrical transducer in cellular physiological activities and is also a focus of attention in piezoelectricity‐mediated tissue repair studies.^[^ ^51^ ^]^ Since the discovery of PIEZO1/2 channels, they have revolutionized our understanding of physiology to open entirely new avenues for translational medicine.^[^ ^52^ ^]^ These channels respond to physical stimuli such as pressure, stretch, and shear, allowing precise regulation of intracellular Ca^2+^ levels in response to mechanical signals.

PIEZO1/2 channels have been shown to mediate calcium signaling in a variety of cells, such as chondrocytes, cultured mesenchymal cells of the lamina propria of the human bladder, human erythrocytes, human dental cells, neurons, and macrophages.^[^ ^53^ ^]^ This Ca^2+^ influx acts as a versatile second messenger, engaging multiple downstream pathways critical for regeneration. Notably, PIEZO‐mediated Ca^2+^ signaling interfaces with the Wnt/β‐catenin and YAP/TAZ pathways—master regulators of cell fate and tissue morphogenesis.^[^ ^54^ ^]^

The role of PIEZO1/2 in bone formation was investigated by Zhou et al.^[^ ^55^ ^]^ They found that PIEZO1/2 channels promote skeletal development in mice by mediating Ca^2+^ inward flow in response to mechanical signals, which in turn activates CaM and its downstream NFAT/YAP1/β‐catenin pathway. Beyond bone, PIEZO channels exhibit diverse, context‐dependent roles in regeneration. In cartilage, studies link PIEZO1/2 activation to chondrocyte apoptosis and matrix degradation in osteoarthritis, suggesting a pathological role.^[^ ^56^ ^]^ In skin wound healing, inhibiting PIEZO1 accelerates keratinocyte migration and reduces scarring, fostering a regenerative phenotype.^[^ ^57^ ^]^ In neural regeneration, PIEZO1 acts as an endogenous inhibitor of axon regrowth, whereas PIEZO2 contributes to neuropathic pain post‐injury.^[^ ^58^ ^]^ These findings highlight the pleiotropic nature of PIEZO channels, necessitating a nuanced understanding of their tissue‐specific roles.

In summary, the discovery of PIEZO1/2 has revolutionized our understanding of how cells sense and respond to mechanical stimuli, with profound implications for physiology and medicine. As research continues to reveal its full function, PIEZO1/2 will likely be central to the development of therapeutic approaches in the field of tissue regeneration.

#### 3.2.3 TRPV4

Transient receptor potential (TRP) channels are a class of non‐selective transmembrane cation‐permeable channels classified into six subfamilies: TRPC, TRPV, TRPM, TRPA, TRPP, and TRPML.^[^ ^59^ ^]^ The mechanically relevant TRP channel that has received the most attention in mammals is TRP vanilloid 4 (TRPV4). Although TRPV4 itself lacks evidence of activation by membrane stretch, it can respond to indirect mechanical stimuli such as fluid shear stress and osmotic swelling to mediate extracellular Ca^2+^ inward flow, which in turn affects cell proliferation.^[^ ^51^, ^60^ ^]^

Through its role as a mechanosensor, TRPV4 significantly influences tissue regeneration across multiple systems, integrating physical and chemical cues into calcium‐mediated responses. In skeletal and cartilage regeneration, TRPV4 regulates chondrocyte proliferation, differentiation, and extracellular matrix synthesis, and its activation contributes to cartilage formation and osteoblast migration.^[^ ^61^ ^]^ In skin wound healing, it drives keratinocyte and fibroblast migration, promoting wound closure, though excessive activation risks pathological scarring.^[^ ^62^ ^]^ In lung repair, TRPV4 maintains epithelial barrier integrity but can exacerbate fibrosis by activating fibroblasts under certain conditions.^[^ ^63^ ^]^ Within the nervous system, it supports neurite outgrowth during development, yet post‐injury activation may worsen spinal cord damage, where inhibition reduces inflammation and scarring.^[^ ^64^ ^]^ In vascular regeneration, TRPV4 facilitates endothelial migration and angiogenesis, though its role in tumor vasculature remains debated, with evidence suggesting both pro‐ and anti‐angiogenic effects depending on context.^[^ ^65^ ^]^ This dual nature, promoting regeneration in some scenarios while driving pathology in others, underscores TRPV4's complex, context‐dependent contributions to tissue regeneration.

### 3.3 ECM/Integrin/FAK

ECM, whose guidance of cellular phenotypes and remodeling are central to the process of tissue repair, is the non‐cellular component of tissues, consisting mainly of collagen, fibronectin, laminin, elastin, proteoglycans, glycosaminoglycans, and other matrix cellular proteins such as Tenascin, Vitronectin, and Osteopontin.^[^ ^66^ ^]^ ECM can interact with the cytoskeleton to provide support and mechanical signals to cells and transmit biochemical signals through various cytokines. Clause et al. summarized the pathways by which ECM promotes tissue repair into three categories: transmembrane receptors such as integrins, proteolytic cleavage such as matrix metalloproteinases (MMPs), and the exposure of cryptic sites such as RGD sites.^[^ ^66a^ ^]^ Today, with the development of quantitative proteomics, decellularization techniques, and databases of ECM by researchers, as well as a deeper understanding of the process of tissue fibrosis and scarring, the idea of targeting or recreating ECM to repair tissues is getting closer to reality.^[^ ^67^ ^]^

The cytoskeleton, composed of actin filaments, intermediate filaments, and microtubules, is an essential skeletal network for cells to sense external stimuli.^[^ ^5a^ ^]^ Integrins are transmembrane dimers composed of α and β subunits, and β1‐type integrins have been shown to be associated with tissue regeneration.^[^ ^68^ ^]^ The head end of the integrin binds to ECM proteins such as collagen, and the tail is attached to actin within the cell, bridging the ECM and the cytoskeleton.^[^ ^5a^ ^]^ FAK is a class of tyrosine kinases located within the focal adhesion that integrins are involved in forming and is a key downstream component of integrins. While much of its research has focused on the invasive behavior of tumor cells and the progression of fibrotic diseases,^[^ ^69^ ^]^ recent evidence suggests an important role in the regeneration of neural and musculoskeletal tissues as well as dentin.^[^ ^70^ ^]^

Activation of the Rho pathway may be a critical downstream mechanism of ECM/Integrin/FAK during tissue regeneration.^[^ ^71^ ^]^ Specifically, FAK is phosphorylated at Tyr397, which binds and activates Src. The activated Src phosphorylates the Tyr925 site of FAK, leading to the binding of GRB2, p190RhoGEF to FAK, which in turn activates the Ras, ERK2, and Rho‐family GTPases (Rho, Rac, and Cdc42) pathways to regulate cell proliferation and survival. Activation of Rho increases the tension of the actinomyosin network by activating MLCK via Rho‐associated coiled‐coil containing protein kinase (ROCK), and it also stabilizes microtubules via the RhoA‐mDia (RhoA effector) pathway. In addition, FAK increases cytoskeletal mobility by decreasing the cross‐linking of α‐actinin to actin and regulates cell migration by affecting cadherin to modulate intercellular contacts.^[^ ^72^ ^]^ Due to the close correlation with the cytoskeleton, it makes sense that MS could modulate the ECM/Integrin/FAK/Rho pathway.^[^ ^73^ ^]^ It is important to note that there is also consensus that ES can modulate the integrin pathway with cell migration, and thus, the ECM/Integrin pathway may be an important pathway for electromechanical synergy to promote tissue repair.^[^ ^74^ ^]^ As mentioned above, both excessive ECM deposition and excessive activation of FAK signaling lead to fibrosis and scarring, so how to effectively utilize the advantageous facets of these mechanisms is an important consideration for electromechanical stimulation, such as controlling the intensity and duration of the electromechanical stimulation or looking for other signals that can inhibit the excessive activation.

### 3.4 Hippo (YAP/TAZ) Pathway

The Hippo signaling pathway, named after the Hippo (Hpo) protein kinase in Drosophila,^[^ ^75^ ^]^ is a pathway closely related to regenerative medicine.^[^ ^76^ ^]^ Transcriptional co‐activators (YAP and TAZ) and core Hippo kinases that inhibit their activity (MST1/2, LATS1/2, SAV1, and MOB1) are central to the pathway. Upstream of the pathway is a complex signaling network, and downstream relies on YAP/TAZ binding to transcription factors (TEAD, p73, RUNX, and TBX5), which promotes cell proliferation, survival, and stemness, and thus the regeneration of cardiac, hepatic, and intestinal tissues in mice.^[^ ^76a^ ^]^ Similar to the ECM/Integrin/FAK pathway, the Hippo pathway has been associated with the progression of cancer and fibrosis, even with the unintended side effect of muscle degeneration.^[^ ^76^ ^]^ In response, Moya et al. summarized four possible strategies for coping with side effects: short‐term activation of the pathway, attenuation of the intensity of activation such as activation of the pathway using the MST1/2 inhibitor XMUMP‐1, tissue‐specific activation, and activation of the pathway target genes such as CCN1/2.^[^ ^76a^ ^]^

Mechanical signaling can regulate YAP activity through four aspects: F‐actin levels, adhesion junctions, focal adhesions, and the nuclear membrane.^[^ ^77^ ^]^ Mechanical stress causes free G‐actin to assemble into polymerized F‐actin^[^ ^78^ ^]^ and binds angiomotin (AMOT) or Arid1A‐SWI/SNF that would otherwise bind YAP, thus allowing YAP to bind transcription factors such as TEAD.^[^ ^77^ ^]^ On the other hand, the inhibition of YAP activity by G‐actin through PKA and AMOT activation of LATS1/2 is attenuated.^[^ ^77^ ^]^ Cadherin conducts tension to the cytoskeleton, leading to conformational changes in α‐catenin linked to actin filaments and ultimately inhibiting LATS1/2.^[^ ^77^ ^]^ After integrins mediate the formation of the FAK/SRC complex formation by responding to mechanical forces, the complex can activate YAP by directly phosphorylating YAP or indirectly inhibiting LATS1/2.^[^ ^77^ ^]^ In addition, cell adhesion deformation results in actin filaments pulling on the nucleoskeletal protein LINC, which flattens the nucleus and increases permeability to YAP, thereby increasing YAP entry into the nucleus.^[^ ^77^ ^]^ Notably, since the integrin pathway can activate YAP activity and Rho has the ability to inhibit LATS1/2, the Integrin/FAK/Rho pathway mentioned above can activate the Hippo pathway and also provides a basis for the indirect activation of the Hippo pathway by ES via the integrin pathway. In addition, ES also appears to induce F‐actin accumulation directly.^[^ ^79^ ^]^

### 3.5 Wnt Signaling Pathway

Wnt signaling is an important pathway that regulates embryonic development and tissue homeostasis^[^ ^80^ ^]^ and is closely associated with hair growth, wound healing, bone formation, neurogenesis, and liver and kidney tissue repair.^[^ ^81^ ^]^ Targeting the Wnt pathway has become one of the core directions in regenerative medicine. The Wnt pathway is categorized into the canonical β‐catenin‐dependent pathway and the non‐canonical Wnt/Calcium and planar cell polarity pathway.^[^ ^81b^ ^]^ In the canonical pathway, the Wnt glycoprotein binds to the transmembrane proteins Frizzled receptor and LRP5/6 co‐receptor, activating the scaffolding protein Dishevelled (Dvl), which recruits the β‐catenin disruption complex (APC/Axin/GSK‐3β/CK1α), leading to the accumulation and ectopic translocation of cytoplasmic β‐catenin to the nucleus binding to the TCF/LEF family of transcription factors. In the non‐canonical pathway, the atypical Wnt ligand binds to Frizzled and the receptor tyrosine kinase‐like orphan receptor 2 (Ror2), and the ligand‐receptor conformer binds to Dvl and G proteins, which further activates the endogenous calcium pathway of PLC/IP3. The Wnt/Frizzled/Ror2/Dvl complex also activates Rho‐family GTPases, further activating the downstream ROCK pathway.^[^ ^81b^ ^]^

Mechanical signals, such as ECM stiffness and intermittent cyclic mechanical tension, have been shown to activate the Wnt pathway.^[^ ^82^ ^]^ Specifically, much of the consensus focuses on the fact that ECM increases β‐catenin via the Integrin/FAK pathway, thereby activating the Wnt pathway, possibly by phosphorylating GSK‐3β leading to the accumulation of β‐catenin and positively feedback increasing the expression of the Wnt1 gene.^[^ ^82b,c^ ^]^ It is important to note that the effects appear to vary from cell to cell and that crosstalk between the Wnt pathway and the Hippo pathway is surfacing, with clearer mechanisms yet to be elucidated.^[^ ^82c^ ^]^ There is also growing evidence that ES activates the Wnt pathway to promote tissue regeneration, mainly in the areas of osteogenesis and nerve regeneration.^[^ ^83^ ^]^ The results show that ES can increase the protein levels of wnt and β‐catenin, but the exact mechanism is not precise, and there may be an indirect role of the integrin pathway.

### 3.6 TGF‐β/SMAD Pathway

The TGF‐β pathway is closely associated with tissue regeneration, such as bone, skin, and intestinal epithelium.^[^ ^84^ ^]^ The TGF‐β signaling consists of two pathways, TGF‐β and bone morphogenetic protein (BMP), a member of the TGF‐β superfamily. Specifically, TGF‐β‐like ligands bind to type II serine/threonine kinase receptors and recruit and phosphorylate type I receptors, which in turn activate receptor‐regulated SMAD proteins (R‐SMAD), with the TGF‐β pathway activated as SMAD2/3 and the BMP pathway as SMAD1/5/9 (8).^[^ ^84^, ^85^ ^]^

Notably, TGF‐β is often secreted in a latent form and requires activation before signaling. Among various activation mechanisms, mechanical forces play a pivotal role, particularly through integrin‐mediated tension.^[^ ^86^ ^]^ Extracellular mechanical cues such as matrix stiffness, cellular contractility, and fluid shear stress induce conformational changes in the latent TGF‐β complex, leading to its activation.^[^ ^86^ ^]^

The coSMAD, usually the SMAD4 protein, binds and translocates R‐SMAD into the nucleus, thereby regulating target genes.^[^ ^84^, ^85^ ^]^ It should be noted that TGF‐β, as a pro‐inflammatory cytokine, also has an important role in the development of tumors, and both sides of the coin need to be utilized wisely.^[^ ^85^ ^]^ Notably, the TGF‐β pathway downstream activates the Rho‐family GTPases and its downstream ROCK pathway, and thus also has a possible endpoint of cytoskeletal regulation.^[^ ^85^ ^]^ This mechanical activation directly links TGF‐β signaling to cytoskeletal dynamics, reinforcing its role in force‐responsive cellular processes.

Similar to the Wnt pathway, the TGF‐β/SMAD pathway can also be regulated by integrins, in this way providing opportunities for electromechanical synergy.^[^ ^87^ ^]^ As mechanosensitive molecules, integrins are key mediators of TGF‐β activation, transmitting extracellular forces to modulate its signaling strength and downstream effects. Specifically, integrins promote the activation of latent state TGF‐β through the protease and the cytoskeletal pathways and can also be activated by TGF‐β, forming a very important integrin‐growth factor positive feedback loop.^[^ ^87^ ^]^ As mentioned above, the integrin pathway is synergistically activated by electromechanics, so it is par for the course that electromechanics can synergistically activate the TGF‐β pathway to regulate behaviors such as cell differentiation.^[^ ^88^ ^]^ In addition, a recent study has shown that cold atmospheric plasma ‐induced electric fields of moderate intensity contribute to the structural stability and ligand‐binding capacity of the type I receptor of the TGF‐β pathway and that electric fields with intensities higher than 0.1 V nm^−1^ are detrimental.^[^ ^89^ ^]^ This again emphasizes that more detailed control of activation methods, crosstalk, and side effects is still an inescapable topic.

### 3.7 Piezoelectric Catalytic Effect

The piezoelectric effect may catalyze the redox reaction of the substrate through energy band theory or screening charge effect, which leads to the direct generation of reactive oxygen species (ROS), with the process known as piezo catalysis.^[^ ^90^ ^]^ ROS has an important role in bactericidal and anti‐infectious properties and, therefore, has a potential value in tissue repair processes that cannot be ignored. Physiological levels of ROS promote stem cell proliferation and differentiation, as well as angiogenesis and bone regeneration.^[^ ^91^ ^]^ However, high levels of ROS are known to be harmful.^[^ ^92^ ^]^ Therefore, controlling ROS generation at a low level is vital to promote tissue regeneration, which is also a point to consider when applying piezoelectric materials.

### 3.8 Mechanoelectric Pathways in Clinical Translation

The seven pathways vary in translational readiness, with TGF‐β/SMAD and Wnt/β‐catenin signaling currently offering the highest therapeutic leverage while emerging routes like piezo‐driven ROS generation remain exploratory. Each pathway plays distinct tissue‐specific roles, such as bone, cartilage, neural, and skin, yet all face knowledge gaps.

TGF‐β/SMAD and Wnt/β‐catenin pathways rank highest in therapeutic potential due to active clinical strategies leveraging their roles in tissue regeneration. The TGF‐β family is already harnessed in therapies. For example, bone morphogenetic proteins, BMP‐2/7, part of the TGF‐β superfamily, are approved to enhance bone repair.^[^ ^93^ ^]^ Antibodies against Wnt inhibitors like sclerostin (romosozumab) are FDA‐approved for osteoporosis, increasing bone formation by unleashing Wnt activity.^[^ ^94^ ^]^ These pathways are pivotal in musculoskeletal and connective tissues: Wnt drives osteogenic and chondrogenic gene programs, which is critical for bone formation and cartilage development,^[^ ^95^ ^]^ while TGF‐β/SMAD signaling governs chondrocyte maturation and scar‐forming myofibroblast activation.^[^ ^96^ ^]^ Notably, both are double‐edged swords biologically—excess Wnt can induce aberrant ossification or tumorigenesis, and TGF‐β can either promote regeneration or, if unchecked, lead to fibrosis. Thus, despite their high translational promise, precision control and context‐specific delivery remain key challenges.

ECM‐integrin‐FAK signaling and mechanosensitive ion channels (PIEZO1/2) form a second tier of therapeutic interest with broad tissue relevance. Integrin‐targeting biomaterials, such as RGD‐peptide coatings, and even small‐molecule FAK inhibitors are being tested to improve healing or reduce fibrosis.^[^ ^62b^ ^]^ Integrin/FAK mechanotransduction is essential for load‐bearing tissues like bone and cartilage as well as skin: integrin engagement under physiological stress can trigger anabolic and anti‐inflammatory responses, for example, focal adhesion kinase activation leading to IL‐4 release and matrix synthesis in cartilage.^[^ ^97^ ^]^ Similarly, PIEZO1/2 ion channels act as primary mechanical sensors across multiple organs—PIEZO1 mediates osteoblast responses to fluid shear and is required for normal bone formation, whereas PIEZO2 is central to touch sensation in skin and neural tissues.^[^ ^95^ ^]^ While no approved therapies directly target PIEZO channels yet, they are viewed as promising targets. For instance, inhibiting PIEZO activity is being studied to reduce chondrocyte overactivation and pain in osteoarthritis.^[^ ^56^ ^]^ The tissue‐specific role of these pathways is evident: moderate mechanical loading that engages integrins and PIEZO channels is generally beneficial, promoting bone density and cartilage homeostasis,^[^ ^95^, ^97^ ^]^ whereas excessive stimulation can be detrimental, overloading a joint disrupts integrin‐cytoskeletal links, activating NF‐κB and catabolic enzymes that degrade cartilage.^[^ ^97^ ^]^ This underscores the translational need to fine‐tune mechanical inputs or dosing of any future PIEZO/FAK‐targeted therapies to maximize regeneration without inducing inflammation.

The Hippo pathway (YAP/TAZ) and intracellular Ca^2+^ signaling are recognized as critical mechanotransducers with somewhat less direct clinical translation so far. YAP/TAZ, as mechano‐responsive transcription co‐activators, orchestrate cell proliferation and differentiation in response to matrix stiffness—they are key to bone and skin tissue adaptation. Active YAP maintains bone/cartilage mass under loading^[^ ^98^ ^]^ and is implicated in skin wound repair,^[^ ^99^ ^]^ but it also contributes to pathology if dysregulated, for example, fibrosis or cancer from constitutive YAP activation. No approved therapies yet target YAP/TAZ directly, though research is intense, such as YAP inhibitors to halt tumor growth or approaches to transiently activate YAP for regenerative purposes.^[^ ^100^ ^]^ The challenge is that Hippo signaling is highly context‐dependent, and upstream triggers are not fully elucidated,^[^ ^100^ ^]^ making it hard to intervene without broad effects. Intracellular Ca^2^⁺ fluxes, on the other hand, are a ubiquitous early step in mechanotransduction: virtually all mechanically responsive cells, from osteocytes to neurons, convert physical stimuli into Ca^2^⁺ signals that regulate downstream pathways. Clinically, this pathway is usually harnessed indirectly. For instance, therapeutic ultrasound or electrical stimulation invokes Ca^2^⁺‐mediated healing responses in bone and muscle without directly drugging Ca^2^⁺ itself.^[^ ^95^ ^]^ While fundamental for tissue function, Ca^2^⁺ signaling's therapeutic targeting is limited by its ubiquity, that is, systemic Ca^2^⁺ modulators lack specificity, so efforts focus on upstream mechano‐sensitive channels, for example, TRPV4 agonists to improve cartilage matrix production via Ca^2^⁺ influx.^[^ ^56^ ^]^ Finally, piezo‐catalytic ROS generation stands out as an innovative, materials‐based modality: by converting mechanical energy into reactive oxygen species, piezoelectric nanoparticles or scaffolds can be used to ablate tumors or pathogens in a targeted manner.^[^ ^101^ ^]^ This approach has shown promise in preclinical cancer models and in eradicating bacteria in infected bone defects, but it remains the least clinically mature of the seven pathways. Its relevance is currently niche (limited to solid tumor therapy and wound disinfection), and translation will hinge on solving issues of precise targeting and safety.

The biological effects of ROS are known to be dose‐dependent; low‐to‐moderate physiological levels act as crucial signaling molecules involved in processes such as wound healing, immune response modulation, and stem cell function, whereas excessive levels lead to cellular damage and impeded regeneration.^[^ ^102^ ^]^ Consequently, harnessing piezoelectric stimulation for tissue regeneration necessitates strategies to precisely manage local ROS concentrations, maintaining them within a beneficial therapeutic window while avoiding detrimental oxidative distress.

Several approaches are being explored to achieve this balance. Optimizing the parameters of external stimuli, such as ultrasound intensity, frequency, and duration, offers a potential route to modulate the piezoelectric response and subsequent ROS generation, although complexities like concomitant ROS production via cavitation remain a challenge.^[^ ^103^ ^]^ Material‐based strategies represent another key direction. These range from selecting piezoelectric materials with intrinsically lower ROS‐generating capacity, to engineering material properties through methods like doping or creating heterojunctions to potentially limit ROS production.^[^ ^104^ ^]^ More prominently, research focuses on integrating ROS‐scavenging capabilities, for instance, by incorporating antioxidants, ROS‐degrading enzymes, or catalytic nanozymes such as ceria or manganese dioxide nanoparticles directly within the piezoelectric scaffold, or by designing ROS‐responsive systems that release protective agents on demand.^[^ ^92^ ^]^ Furthermore, combination strategies that focus on managing the overall tissue microenvironment, such as utilizing the piezoelectric effect to promote immunomodulation towards a pro‐regenerative M2 macrophage phenotype, can help counteract potential ROS‐induced inflammation and create a more favorable milieu for repair.^[^ ^103^ ^]^ Ultimately, the goal is to leverage the beneficial mechanoelectrical signaling pathways while actively mitigating or balancing the associated ROS production, thereby ensuring the safe and effective translation of piezoelectric biomaterials in regenerative medicine.

Overall, pathways like TGF‐β and Wnt already have clinical or near‐clinical interventions, whereas integrin/FAK and mechanosensitive Ca^2^⁺ channels are on the cusp of translation via advanced biomaterials and devices. YAP/TAZ represents a mechanobiology cornerstone with indirect translational strategies being explored. Piezoelectric ROS generation is a relatively new concept showing promise for future therapies. This ranking may evolve as new findings emerge, but it provides a framework for prioritizing piezoelectric targets for clinical translation. The following table summarizes these knowledge gaps and practical research directions to address them, aiming to drive each pathway closer to safe and effective clinical use (**Table** **2**).

**Table 2:** Knowledge gaps and research directions of the seven piezoelectric pathways, from a translational perspective.

Pathway	Knowledge gaps	Research directions
Intracellular Ca^2^⁺	Hard to target specifically (broad roles). Limited in vivo spatiotemporal data. Uncertain links to downstream transcription.	Develop upstream channel modulators. Use live Ca^2^⁺ imaging to track signals under load. Use of controlled piezoelectric stimulation to monitor systemic effects
Ion Channels (PIEZO1/2)	Few selective modulators; many are not clinical‐grade. Limited disease‐focused channel regulation. Risk of off‐target effects (widespread expression).	Identify safer Piezo agonists/antagonists. Explore tissue‐specific gene therapy for precise up/down‐regulation. Develop localized mechanotherapeutics.
ECM/Integrin/FAK	Unclear dose‐response. Redundancy and variety in the integrin family. FAK signaling crosstalk.	Biomechanical dose studies. Integrin‐subtype therapies (antibodies/peptides). Test FAK modulators in tissue‐specific models.
Hippo (YAP/TAZ)	Unknown upstream force sensors. Context‐specific behavior. Lack of precise, tissue‐specific modulators.	Characterize how piezoelectricity gets converted to YAP/TAZ activity. Develop selective inhibitors/activators. Tune substrate stiffness in piezoelectric tissue engineering.
Wnt/β‐catenin	Systemic activation risks ectopic growth or tumors. Wnt proteins are unstable, making delivery tricky. The tipping point remains unclear.	Localize Wnt delivery (scaffolds, hydrogels). Refine strategies that target Wnt inhibitors (sclerostin, DKK1). Engineer context‐specific Wnt responses to piezoelectric cues.
TGF‐β/SMAD	Mechanistic ambiguity. Isoform‐specific roles insufficiently understood. Immunoregulatory side effects.	Pursue isoform‐focused treatments. Exploit latent TGF‐β activation with electromechanical triggers. Combine TGF‐β modulators with controlled load to reduce scarring.
Piezo‐catalytic ROS	Targeting and safety issues. Material biocompatibility and clearance. Dose‐response data for deep tissue therapy are limited.	Develop site‐specific piezocatalysts (triggered by pH or biomarkers). Standardize ultrasound settings for safe ROS generation. Combine with existing therapies to reduce the required ROS dose.

## 4 Piezoelectric Materials for Tissue Regeneration

### 4.1 Overview of Piezoelectric Materials in Tissue Engineering

**Figure 2:**
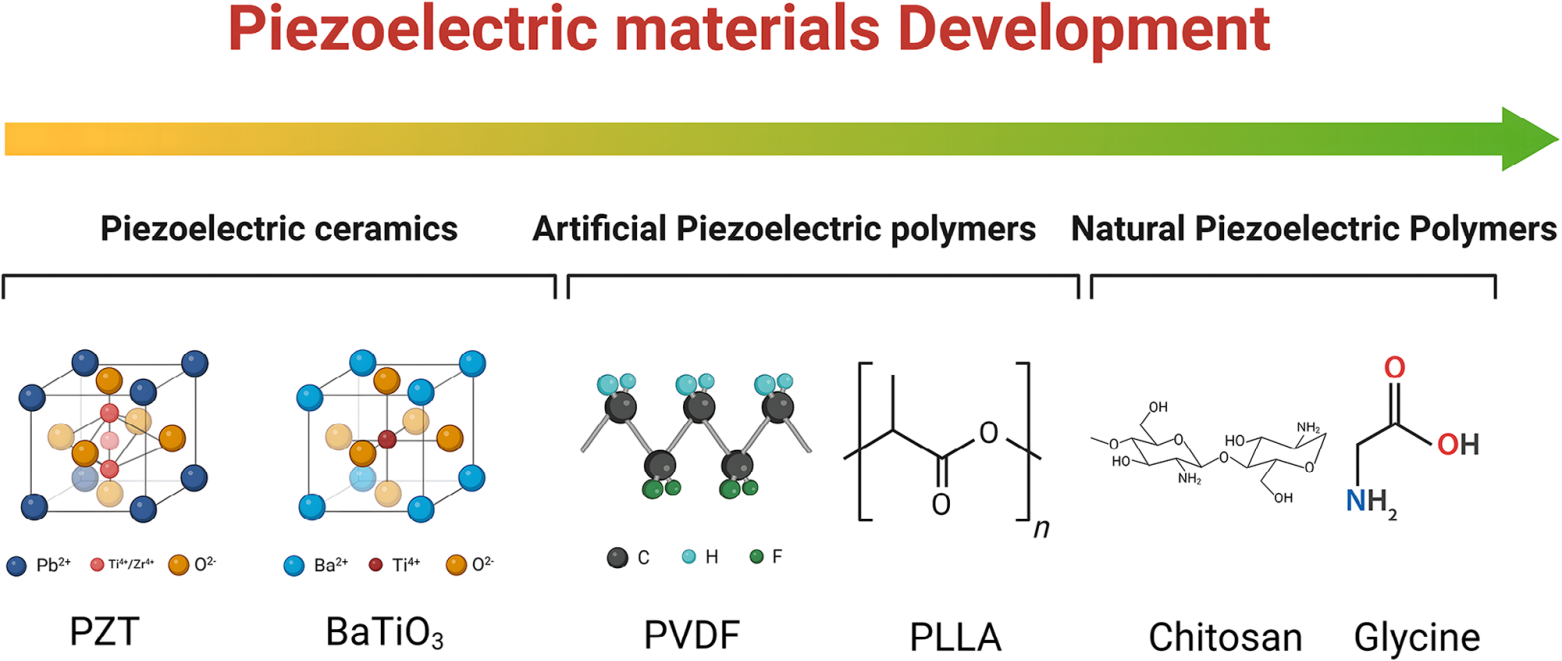
Brief history of piezoelectric materials. Piezoelectric materials have gone through three main stages: piezoelectric ceramics, artificial piezoelectric polymers, and natural piezoelectric polymers. Piezoelectric ceramics are represented by PZT and BaTiO_3_, and artificial piezoelectric polymers are represented by PVDF and PLLA. Natural piezoelectric polymers are emerging, including chitosan and glycine. Created in BioRender. (2024) https://BioRender.com/y99j480.

Piezoelectric materials are categorized into inorganic piezoelectric ceramics, organic piezoelectric polymers, piezoelectric composites consisting of piezoelectric ceramics and flexible polymers, and natural piezoelectric materials.^[^ ^12^, ^105^ ^]^ The evolution of piezoelectric materials is briefly represented in **Figure** **2**. The earliest piezoelectric ceramics were barium titanate (BaTiO_3_, BT) ceramics reported by Gray et al. in 1946, after which the strongly piezoelectric lead‐based piezoelectric ceramics, PZT, was discovered in 1952, and the d_33_ of PZT‐5H could be as high as 593 pC N^−1^.^[^ ^105^, ^106^ ^]^ Since the toxicity of elemental lead is unsuitable for biomedical applications, further in‐depth research has been carried out on lead‐free piezoelectric ceramics, which, in addition to the BTs mentioned above, include chalcocite‐based piezoelectric ceramics such as BiNaTiO_3_ (BNT), BiKTiO_3_ (BKT) and KNaNbO_3_ (KNN), tungsten‐bronze‐based piezoelectric ceramics, and bismuth‐layered‐structured piezoelectric ceramics.^[^ ^107^ ^]^ Organic piezoelectric materials, namely piezoelectric polymers, have received focused attention in the biomedical field due to their excellent biocompatibility and deformation ability, although their macroscopic piezoelectric coefficients are significantly inferior to those of piezoelectric ceramics. Piezoelectric polymers applied to tissue regeneration mainly include polyvinylidene fluoride (PVDF), polyvinylidene fluoride‐trifluoro ethylene (P(VDF‐TrFE)), and poly‐l‐lactic acid (PLLA).^[^ ^108^ ^]^ PVDF and P(VDF‐TrFE) have the strongest piezoelectric properties, with d_33_ reaching about 30 pC N^−1^ after stretching and polarization.^[^ ^105^, ^106^, ^109^ ^]^ Piezoelectric composites are currently a major research direction, which can bring together the electromechanical properties of piezoelectric ceramics and the mechanical properties of piezoelectric polymers. For example, PVDF can be combined with PZT or BT to form piezoelectric composites to enhance the piezoelectric response of the PVDF material alone.^[^ ^110^ ^]^ In addition, natural piezoelectric materials such as chitosan and glycine are gradually beginning to be used in biomedical applications.^[^ ^111^ ^]^ Besides amino acids and polysaccharides, peptides, proteins, and viruses are also potential natural piezoelectric materials.^[^ ^112^ ^]^ Many well‐written publications have summarized the piezoelectric and mechanical properties of the above classical materials, so it will not be repeated here.^[^ ^12^, ^105^, ^106^, ^109^ ^]^ We will provide an in‐depth introduction to applying novel piezoelectric composites in tissue regeneration in Part V.

### 4.2 Fabrication Process and Improvement of Piezoelectric Materials

Fabrication processes can improve the piezoelectric properties, mechanical properties, and biocompatibility of piezoelectric materials, among other things. Preparation and modification processes clearly vary for different materials. Waqar et al. summarized the methods to enhance the piezoelectric performance of lead‐free piezoelectric ceramics, including optimization of lattice, domain, phase boundary and interface, and defect engineering.^[^ ^113^ ^]^ Specifically, the above methods to enhance piezoelectric properties may be based on the following principles described in the text. At the lattice level, the higher the polarization rate and the greater the hybridization ability of the cation, the stronger the macroscopic piezoelectric effect exhibited. Small regions with the same direction of spontaneous polarization are called domains, and the boundaries between domains are called domain walls. The piezoelectric response of a ferroelectric material consists of the polarization extension and polarization rotation at the lattice level, as well as the motion of domain walls. Optimizing domains to enhance piezoelectricity includes configuring optimal domain configurations by polarizing in different directions, reducing domain size by changing polarization or chemical composition, and promoting the formation and stabilization of charged domain walls. Phase transition refers to a change in crystal structure driven primarily by composition or temperature, and materials near the phase boundary require less free energy for polarization and exhibit a stronger piezoelectric response. The phase transitions in Pb‐based and Pb‐free piezoelectric ceramics are driven by the composition and temperature of the components, respectively, and are referred to as morphotropic phase boundary and polymorphic phase boundary.^[^ ^113^ ^]^ The main effort in phase boundary engineering is to prepare piezoelectric materials that can be stabilized near the phase boundary at room temperature by chemical modification and other means. In addition, interfaces between different phases or different compositions may also improve the piezoelectric response through elastic, electrostatic, and electronic effects. Defect engineering, mainly manipulating oxygen vacancies, is an important method for improving lead‐free piezoelectric ceramics, which has emerged in recent years.^[^ ^114^ ^]^

As the most common piezoelectric polymer in the biomedical field today, PVDF has excellent piezoelectricity, flexibility, physicochemical stability, and biocompatibility.^[^ ^115^ ^]^ PVDF exhibits five crystal structures, namely α, β, γ, δ, and ε phases. Among them, the α phase is the only non‐polar phase with the best thermodynamic stability, and the β phase is the most piezoelectric.^[^ ^115a^ ^]^ Methods to increase the β‐phase content in PVDF include mechanical stretching, strong electric field polarization, low‐temperature quenching, annealing, hot pressing, formation of copolymers such as P(VDF‐TrFE), appropriate addition of fillers such as particles, piezoelectric, conductive, and hard materials, and nanoconfinement effects.^[^ ^115b^ ^]^ The main methods for manufacturing PVDF‐based materials include melt blending, solvent casting, 3D printing, and electrospinning.^[^ ^115a^ ^]^ Among them, electrospinning is perhaps the most popular technique for preparing piezoelectric scaffolds due to the combination of stretching and polarization and the good mimicry of the extracellular matrix by the spun material.^[^ ^115a,b^ ^]^ Ways to improve the electrospinning process mainly include optimization of electrospinning parameters (voltage, flow rate, collection distance, and so on), solution properties (polymer molecular weight, solution concentration, solvent system, and so on), and environmental parameters (temperature and humidity).^[^ ^116^ ^]^ In addition, single‐axis electrospinning is gradually starting to transition to multi‐axis and coaxial electrospinning for more complex application scenarios.^[^ ^116^ ^]^ Plasma technology is a classical material surface modification technology that can improve hydrophilicity properties, pro‐cell adhesion, proliferation ability, biocompatibility, and even realize the self‐healing of materials.^[^ ^117^ ^]^ It is widely used for surface optimization of flexible piezoelectric materials like PVDF, which has an extensive potential for application.^[^ ^118^ ^]^ Notably, Haick and colleagues have extensively explored potential applications and improvement strategies for self‐healing materials. These flexible, self‐repairing materials offer a promising framework for the design of piezoelectric biomaterials in tissue regeneration, potentially enhancing both the durability and wearability of piezoelectric devices.^[^ ^119^ ^]^

**Figure 3:**
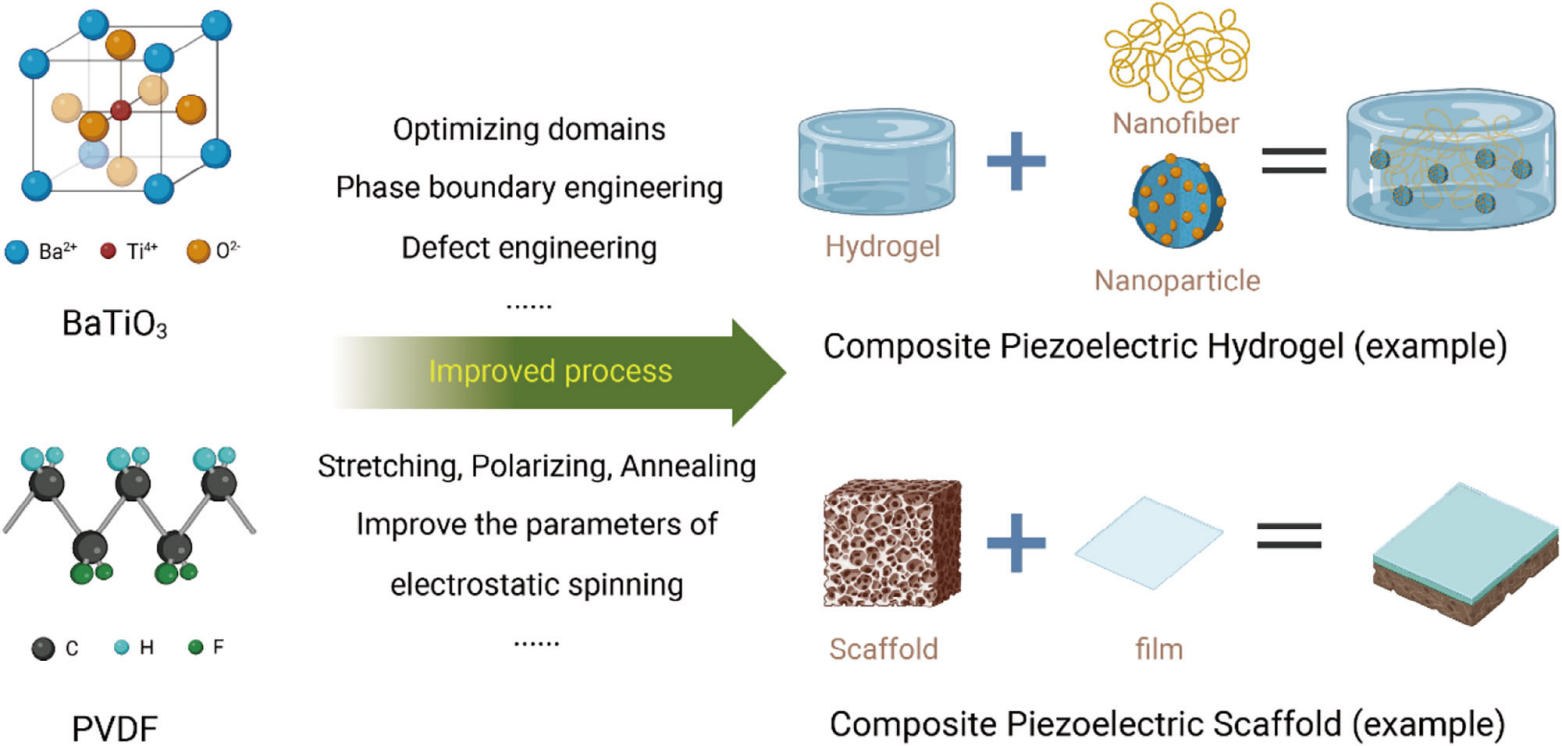
Schematic diagram of the fabrication of piezoelectric composite hydrogel and scaffold. The first example is the enhancement of BaTiO₃ nanoparticles by domain optimization, phase boundary engineering, and defect engineering, followed by a combination with nanofibers and hydrogel matrix to form a composite piezoelectric hydrogel. The second example is the enhancement of PVDF films by stretching, polarization, annealing, and optimization of electrostatic spinning parameters, which are then combined with porous scaffolds to form composite piezoelectric scaffolds. Existing approaches emphasize the improvement of piezoelectric properties through material processing and combinatorial techniques. Created in BioRender. (2024) https://BioRender.com/g01t330.

Piezoelectric materials are designed in different morphologies, such as hydrogels, nanoparticles (NPs), thin films, and wires/rods/tubes, in order to suit different application scenarios.^[^ ^120^ ^]^ Due to their inherent good biocompatibility, biodegradability, and similarity to the extracellular matrix, hydrogels have the potential to mimic the bioelectrical microenvironment of tissues after the introduction of piezoelectricity and have thus been emphasized in wound healing and tissue regeneration engineering in recent years.^[^ ^120a^ ^]^ Specifically, piezoelectric hydrogels are categorized into natural polymer hydrogels (filipin, chitin, chitosan, collagen, and bacterial cellulose), which are assembled mainly by physical cross‐linking and synthetic polymer hydrogels (polyacrylamide, PAM, polyacrylonitrile, PAN, and poly(vinyl alcohol), PVA), which have nearly perfect biocompatibility and degradability, and the latter is mainly chemically crosslinked and has strong flexibility.^[^ ^120a^ ^]^ The introduction of organic (PVDF) or inorganic piezoelectric materials (BT) is used to enhance the piezoelectric properties of hydrogels but reduces their flexibility. Therefore, the goal of piezoelectric hydrogels at this stage of research and development is to simultaneously achieve excellent piezoelectric properties, flexibility, biocompatibility, and degradability.^[^ ^120a^ ^]^ Piezoelectric materials Another common material is piezoelectric NPs, of which BaTiO_3_ NPs are valued in the field of tissue engineering due to their negligible cytotoxicity, cell‐targeting ability, and photosensitizing generation of ROS.^[^ ^120b^ ^]^ Due to its combination of optical and ferroelectric properties, there is an opportunity to synchronize therapeutic and image monitoring. BT NPs are prepared by co‐precipitation, hydrothermal, solvothermal, sol‐gel, and high‐energy milling methods.^[^ ^120b^ ^]^ To achieve better particle dispersion and biocompatibility, the surface modification of particles is usually carried out by hydroxylation, polyethylene glycolization, and gold (AURNM, Au) modification.^[^ ^120b^ ^]^ The durability, subcellular localization, and biosafety of piezoelectric NPs need to be further explored. Thin films are classical two‐dimensional materials, the most common of which are probably PVDF‐based piezoelectric films.^[^ ^120f^ ^]^ The main preparation methods for piezoelectric films include spin‐coating, solvent evaporation, and electrostatic spinning, of which electrostatic spinning is the most versatile technique, with modifications as described above. BT NPs, graphene and its oxides, nanoclays, and carbon nanotubes have also been used as additives to increase the piezoelectric properties of PVDF films. Ultra‐thin piezoelectric films may be a potential development, but preparation is difficult.^[^ ^121^ ^]^ One‐dimensional piezoelectric materials such as piezoelectric nanowires, nanorods, and nanotubes are another focus of the field.^[^ ^120c–e^ ^]^ Nanotubes are hollow nanostructures, and nanowires and nanorods are solid nanostructures. The former have aspect ratios greater than 20, and the latter have aspect ratios greater than one but less than 20. Template‐assisted, electrostatic spinning, and molten‐salt methods are the most common syntheses of nanotubes, nanowires, and nanorods, respectively.^[^ ^120d^ ^]^ Although they have good mechanical and chemical stability, their piezoelectric properties are weaker than those of chalcogenide ceramics, and PFM and atomic force microscope (AFM) are used to evaluate the piezoelectric behavior of the materials. The main ways to enhance the piezoelectric properties are to improve the size and crystal structure of the material, chemical doping, and morphological phase boundary engineering.^[^ ^120e^ ^]^ Most of the current studies use composites of the above materials, such as composite scaffolds and composite hydrogels (**Figure** **3**).

### 4.3 Topological Structure of Biological Materials in Tissue Regeneration

As mentioned earlier, mechanical signals closely influence tissue regeneration. Varghese and colleagues have conducted extensive research in biomechanics, demonstrating that the physical properties and interfacial architecture of biomaterials can profoundly influence cellular behavior.^[^ ^122^ ^]^ Changes in cell shape have been shown to affect lipid raft assembly directly, trigger the serine/threonine protein kinase B pathway, known as the Akt pathway, which directs MSC differentiation, and that triangular and square (highly contractile) cell shapes promote osteogenic differentiation, and round (low contractile) cell shapes promote lipogenic differentiation.^[^ ^123^ ^]^ Additionally, topology is closely correlated with cell migration.^[^ ^124^ ^]^ Concretely, cells can sense substrate stiffness and topology through integrins, MSICs, and the cytoskeleton, which in turn promotes signaling cascades, such as the calcium pathway, that direct their chemotaxis.^[^ ^124^, ^125^ ^]^ Topological defects in the epithelial cell layer itself, “comet‐like defects,” have also been shown to be associated with apoptosis and extrusion, suggesting that topological cues have an important influence on cell behavior.^[^ ^126^ ^]^ The topology of the charge distribution on the material's surface also profoundly affects cell proliferation, differentiation, adhesion, and migration.^[^ ^127^ ^]^ Positively charged polymer surfaces may be closely associated with tissue regeneration by improving hydrophilicity or by adsorbing specific cells and proteins to promote cell adhesion, proliferation, and differentiation. Zeta potential is the most common means of evaluating the charge indicated by a material.^[^ ^127^ ^]^ In summary, the topology of the material, that is, the physical characteristics and geometrical layout of the contact surface between the material and the cell, is of great significance in the field of tissue engineering and is an essential aspect to focus on in the design of piezoelectric materials.^[^ ^128^ ^]^

**Figure 4:**
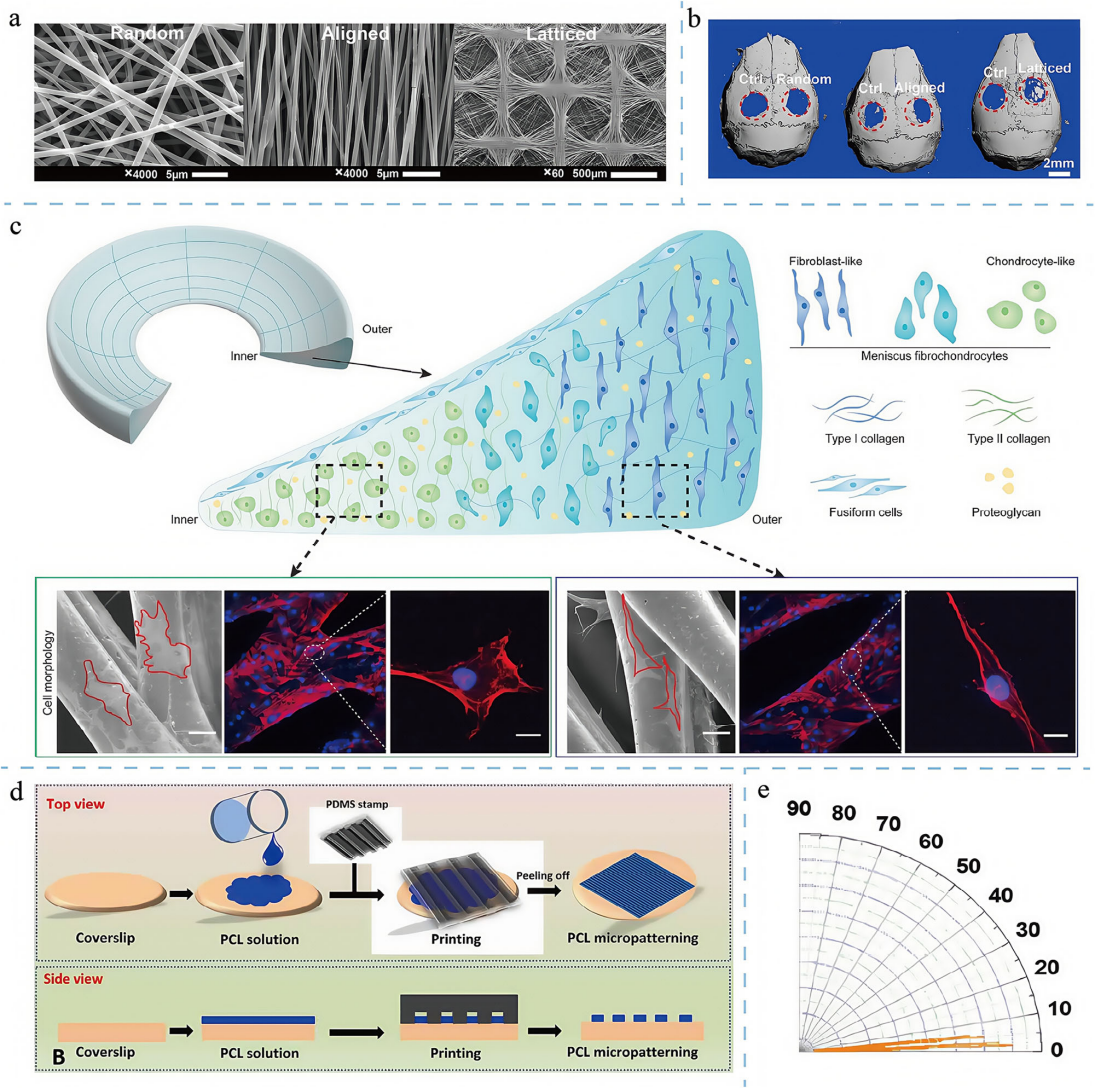
Topological materials for bone, cartilage, and nerve regeneration. a) SEM images of randomized, aligned, and latticed poly (lactate‐co‐glycolate)/fish collagen/nano‐HA fibrous membranes. b) Latticed structure has the strongest ability to repair cranial defects. Reproduced with permission.^[^ ^129b^ ^]^ Copyright 2021, Elsevier. c) Gradient‐sized diamond‐pored microstructure induces heterogeneous differentiation of BMSC. The inner is fibroblast‐like cells, and the outer is chondrocyte‐like cells. Scale bars, 50 µm. Reproduced with permission.^[^ ^129c^ ^]^ Copyright 2024, Ke Ai Publishing. d) Process of making micropatterned/aligned PCL scaffolds. e) Micropatterned/aligned PCL scaffolds induce directed growth of Schwann cells. Reproduced with permission.^[^ ^129d^ ^]^ Copyright 2021, American Association for the Advancement of Science.

The design of topological structures has played a rather popular role in tissue regeneration, particularly in repairing tissues such as bone, cartilage, and nerves (**Figure** **4**).^[^ ^129^ ^]^ Liu et al. prepared electrostatic spinning polycaprolactone (PCL) scaffolds with aligned fibers for inoculation of rat bone marrow mesenchymal stem cells (rBMSCs) supplemented with cyclic mechanical tensile stimulation (deformation ≤ 1%, displacement = 2 mm, frequency = 0.5 Hz) and compared with randomly oriented PCL scaffolds.^[^ ^129a^ ^]^ The results showed that there was no statistical difference in the proliferation ability of the two groups of rBMSCs in the absence of MS, whereas rBMSCs on the aligned scaffolds showed the ability to grow directionally along the fibers. MS promotes the ability of rBMSCs to grow along the direction of mechanical force in both groups, but it does not seem significant in the random group, implying that topology may have a higher priority than mechanical signals. Moreover, real‐time fluorescence quantitative PCR was performed on the osteogenic genes Runx2 and BMP‐2, and the results showed that both fiber alignment and stretch stimulation significantly promoted the expression of osteogenic genes.^[^ ^129a^ ^]^ Jin et al. prepared poly (lactate‐co‐glycolate)/fish collagen/nano‐HA (PFCH) fibrous membranes and designed three topologies, namely, random fibers, aligned fibers, and lattice‐like fibrous networks (Figure 4a).^[^ ^129b^ ^]^ Unlike the previous study, this study found that the lattice topology contributes to the initial proliferation of BMSCs, monocyte, and macrophage recruitment, M2‐type polarization of macrophages, angiogenesis, and eventual new bone formation (Figure 4b) in the region of cranial defects in rats, by a mechanism that may be the activation of the HIF‐1α signaling pathway, which further stimulates the VEGF and NOS pathways.^[^ ^129b^ ^]^ Du et al. designed a bionic meniscus PCL scaffold with gradient‐sized diamond‐pored (GSDP) microstructure using 3D printing and treated it with alcohol and NaOH solution to improve the roughness and hydrophilicity of the scaffold surface.^[^ ^129c^ ^]^ BMSCs were isolated from the bone marrow of New Zealand white rabbits. Compared with PCL scaffolds with uniform square and rhombus pores, the GSDP scaffold has good mechanical bionic properties and can induce the bionic‐specific differentiation of BMSCs (Figure 4c), showing the best meniscus regeneration and protection ability. Specifically, the chondrocyte‐like matrix and round cells are distributed on the inner side of the scaffold, and the fibrous tissue and spindle‐shaped cells are distributed on the outer side of the scaffold, simulating the biochemical heterogeneity of the natural meniscus. The pathways activated in the inner region of GSDP are mainly the p53 and TGFβ pathways, and the outer region is mainly the PI3K‐Akt pathway.^[^ ^129c^ ^]^ Li et al. prepared four PCL scaffolds, including random, aligned, micropatterned, and micropatterned/aligned (MA) scaffolds (Figure 4d), using the electrospinning process, and modified them with IKVAV peptide to evaluate the effect of the topological structure on Schwann cell behavior.^[^ ^129d^ ^]^ The results showed that Schwann cells in the MA/IKVAV group had the strongest directional growth ability (Figure 4e) and angiogenesis ability and could promote the expression of myelin‐related genes MBP and Sox10.^[^ ^129d^ ^]^ Gao et al. introduced topology into piezoelectric materials. By dispersing dopamine (PDA)‐modified BT particles in ovalbumin droplets, further imprinting the droplets with polydimethylsiloxane (PDMS) containing ridge/trough topology, and removing the PDMS after natural air drying, they obtained ovalbumin/BaTiO_3_ piezoelectric scaffolds with topology.^[^ ^129e^ ^]^ The results show that this topological scaffold has good mechanical properties and high piezoelectric output after polarization and can promote the directional growth of Schwann cells and DRG axons. CNTN2 and TAZ may be key mechanism genes.^[^ ^129e^ ^]^ Regrettably, the research did not go into more in‐depth design and comparison of the topology but instead focused on the concentration of BT particles, finding that 5% was the optimal concentration. Kong et al. summarized the two‐dimensional alignment design and three‐dimensional topological design in peripheral nerve regeneration, emphasizing the importance of multi‐lumen nerve scaffolds in guiding nerve bionic repair.^[^ ^130^ ^]^

### 4.4 Biosafety of Piezoelectric Materials

As an external implant, piezoelectric materials still have many issues to be resolved before they can be clinically translated. Their biosafety is a critical topic that must be addressed. At present, there is a lack of discussion on the side effects of piezoelectric material implantation reactions, but there are three possible aspects: cytotoxicity, implant infection, and side effects of ES. It is important to note that foreign body reactions (FBRs) to implants also include FBRs that cause fibrosis and immune imbalances, which we will cover in‐depth in the next section.

The cytotoxicity of materials is mainly related to the material's elemental composition, as metal ions released from piezoelectric ceramics may induce excessive production of ROS. For example, Mg, Pb, and Fe are cytotoxic to the chromaffin PC‐12 cells, a model of nerve cells, mainly via the formation of MgSO_4_, Pb(NO_3_)_2_, FeCl_2,_ and FeCl_3_.^[^ ^131^ ^]^ These metal elements are also often found in piezoelectric ceramics or piezoelectric composites. Another common piezoelectric material, ZnO, also significantly inhibits neuronal and cardiac muscle cell lines due to the release of free zinc ions.^[^ ^132^ ^]^ The supernatant of KNN powder soaked in the medium for 24 h inhibited 16% cell activity, and the addition of lithium to KNN increased cytotoxicity to 42%. This may be related to the release of Li and Nb ions and the pH change during degradation.^[^ ^133^ ^]^ Solutions may include selecting less toxic common elements or directly reducing the release of toxic ions. Guo et al. used the sol–gel method to coat TiO_2_ sheaths on the surface of ZnO nanowires and calcined them with ammonia to incorporate nitrogen, resulting in a new type of ZnO nanowires (NWs), ZnO NWs@TiO_2‐x_N_y_.^[^ ^134^ ^]^ After 120 h of co‐culture with L‐929 fibroblasts, the cell viability remained above 90%. They speculated that the sheath layer of the material reduced the release of free zinc ions. This is a creative idea, but it may involve lower material degradation and secondary trauma to the body when the material is removed. Therefore, more in‐depth thinking is still necessary.

Infection caused by implants is a classic problem. In fact, some methods have been developed to strengthen the response to implant infections. Catalyzing the production of ROS to kill bacteria at the implant site is the main method. For example, Sun et al. prepared a BaTiO_3‐x_/l‐arginine complex, which has a good ROS and NO yield under the piezoelectric effect of ultrasonic excitation and promotes the polarization of macrophages to M1 type, with obvious killing ability against methicillin‐resistant Staphylococcus aureus.^[^ ^135^ ^]^ Similarly, Xu et al. prepared a piezoelectric PCL film loaded with Janus NPs BT@Au, which has the ability to generate ROS with sensitive ultrasonic response, and the antibacterial rate against Staphylococcus aureus subcutaneous in rats reached 96.9%.^[^ ^136^ ^]^ However, studies have shown that tissues and cells have a tolerance and clearance effect on inflammation caused by implants, so it is necessary to consider whether the induction of ROS will aggravate the metabolic disorder of tissues, that is, whether the harm outweighs the benefit.^[^ ^137^ ^]^ Moreover, as mentioned in the mechanism section above, materials with piezoelectric properties inherently have certain piezoelectric catalytic ROS generation and antibacterial abilities, so the pursuit of more ROS generation may be limited to the regeneration of tissues with a high risk of infection. In addition to increasing ROS production, another method of antibacterial action is to construct positive charge traps on the implant surface to capture negatively charged bacteria. Sun et al. constructed a hierarchical TiO_2_ nanotube (NT) layer with positive charge traps and a PVDF layer with an appropriate pore size and negative charge on the surface of a titanium implant. The results showed that *Staphylococcus aureus* and *Escherichia coli* were adsorbed on the NT layer, and the cell membrane was destroyed, with antibacterial abilities of 30.4% and 61.9%. The structure also promoted the polarization of M1 macrophages to the M2 type and promoted bone formation.^[^ ^138^ ^]^ Compared to enhancing ROS production, the antibacterial method of building a charge trap may be gentler. It is important to note that different clinical application scenarios place different requirements on the biosafety of materials. In open systems such as dental implantation or wound healing, the material may be directly exposed to the external bacterial environment, and the risk of infection is higher, so stronger antimicrobial strategies (e.g., higher levels of ROS generation) may have a more pronounced advantage in controlling infections; whereas, for complete implants, since they are located in a relatively closed in vivo environment, even lower levels of ROS may cause local cytotoxicity or chronic inflammation, thereby affecting the long‐term stability and function of the implant. Future studies should systematically evaluate the long‐lasting biosafety and immune responses of these antimicrobial materials in both open and closed systems in models that are more closely aligned with real‐world clinical applications in order to ensure their safety and efficacy in specific clinical contexts.

Possible side effects of ES are also a matter of concern. For example, Zeng et al. used 0–2 mA current to stimulate the ear canal of tinnitus patients intermittently, which caused pain and paresthesia.^[^ ^139^ ^]^ Saranya et al. showed that microcurrent nerve stimulation at 0.5 Hz and one mA was more effective than transcutaneous electrical nerve stimulation at 50 Hz and 0–60 mA in reducing pain (VAS score) in the masticatory muscles.^[^ ^140^ ^]^ Ren et al. found that pulsed DCEF (150 mV mm^−1^, D = 60%, f = 0.1 Hz) had significantly less effect on pH, temperature, and cell proliferation than constant DCEF (150 mV mm^−1^). It also triggered the electrotaxis of keratinocytes via phosphorylation of ERK1/2.^[^ ^141^ ^]^ The intensity of the ES can be controlled by changing the stimulation method or controlling the stimulation parameters. In tissue regeneration, piezoelectric materials can be activated in three ways: cell traction, physiological mechanical movement, and ultrasound (US), producing ES of different intensities.^[^ ^142^ ^]^ Currently, quantitative discussions on the generation of ES by piezoelectricity are still insufficient, and more research and consensus are needed to clarify the safety threshold of piezoelectric stimulation in tissue regeneration applications.

## 5 Collagen and Inflammatory Shifts in Piezoelectric Tissue Regeneration

Having elucidated the clinical biosafety considerations, we will approach the critical discussion of contemporary applications. However, prior to this progression, it is imperative to provide an in‐depth exposition of collagen dynamics and inflammatory transformation within piezoelectric contexts. These pivotal concepts will reinforce our understanding of the preceding sections while establishing essential contextual foundations for subsequent discussion. Moreover, we provide guiding clinical translation perspectives to enhance the translational relevance of this discourse.

### 5.1 Collagen Regulation in Piezoelectric Regeneration Contexts

As stated in section 2.2, collagen is the most important component of biological piezoelectricity. In mineralized bone, type I collagen forms the organic scaffold that imparts tensile strength and toughness, whereas hyaline cartilage is composed of ≈90–95% type II collagen, conferring load‐bearing elasticity and resistance to shear.^[^ ^143^ ^]^ In wound healing (such as skin), fibroblasts initially lay down type III collagen as a temporary matrix, later replaced by type I collagen for stronger scar tissue. An optimal outcome (e.g., in bone fracture or dermal wound) requires enough collagen to restore structural integrity but not the unchecked overproduction of collagen I/III that creates non‐functional fibrotic tissue.^[^ ^144^ ^]^ However, unrestrained collagen synthesis can yield hypertrophic scar or fibrosis, wherein dense type I collagen replaces normal tissue architecture. TGF‐β is the master regulator of collagen production in injury responses, orchestrating a balance between necessary matrix deposition and pathological excess.^[^ ^145^ ^]^ Excess TGF‐β signaling or prolonged inflammation can drive continued collagen synthesis and myofibroblast activation, culminating in scar formation.^[^ ^145^ ^]^

Piezoelectric stimulation offers a means to influence this collagen balance through mechanoelectrical signaling. When a piezoelectric scaffold is mechanically stressed—for example, by body movement or pulsatile forces—it generates localized electrical potentials that directly modulate cell behavior.^[^ ^143^ ^]^ These cues can activate the related signals mentioned in Section 3, such as VGCC, TGF‐β, and integrin pathways, and promote extracellular matrix synthesis, including collagen deposition for repair. The integrin‐growth positive feedback loop (mentioned in Section 3.6) under electromechanical stimulation tends to align collagen deposition with mechanical demand, encouraging organized matrix architecture. For instance, tensile forces normally orient collagen fibrils along the lines of stress during remodeling—piezoelectric materials that convert those forces into electrical signals may amplify such organized remodeling.^[^ ^145^ ^]^ In cartilage regeneration, electrical stimulation has been shown to tilt the balance of collagen production toward regenerative phenotypes: in vitro studies demonstrate that exogenous electrical cues can suppress type I collagen while increasing type II collagen, aggrecan, and Sox9 expression, thereby promoting the formation of hyaline‐like cartilage over scar tissue.^[^ ^146^ ^]^ Such findings underscore that piezoelectric cues can modulate not just the quantity but the quality of collagen in healing tissues, steering the process toward functional regeneration rather than fibrosis.

### 5.2 M2 Macrophage Polarization and Piezoelectric Stimulation

The inflammatory milieu plays a decisive role in how the collagenous matrix is laid down and remodeled. Particularly, macrophage phenotype shifts are pivotal in tissue repair. Pro‐inflammatory M1 macrophages dominate early after injury, clearing debris and bacteria, but a timely transition to the anti‐inflammatory M2 phenotype is essential for constructive healing. M2 macrophages secrete an array of cytokines, such as IL‐10 and IL‐4, and growth factors, including TGF‐β₁, VEGF, and PDGF, that dampen inflammation and actively encourage tissue rebuilding.^[^ ^147^ ^]^ Therefore, actively skewing the immune response toward M2 macrophages is a promising strategy to improve regeneration outcomes in both bone and soft tissue contexts.

Recent evidence shows that dynamic piezoelectric stimulation can reprogram macrophages toward the M2 phenotype, creating a pro‐regenerative inflammatory profile.^[^ ^148^ ^]^ The electromechanical stimulation is believed to recapitulate natural endogenous signals that encourage an anti‐inflammatory, pro‐healing response.^[^ ^147^ ^]^ Indeed, electrically active or positively charged material surfaces are known to be conducive to M2 polarization, whereas neutral or negative surfaces tend to induce an M1 phenotype.^[^ ^147^ ^]^ By mimicking the native electrical microenvironment of healing tissue, piezoelectric scaffolds can thus tilt the M1/M2 balance. Macrophages guided into an M2 state by these cues subsequently secrete mediators that further orchestrate regeneration. Notably, M2 macrophages produce high levels of TGF‐β₁ and IL‐10; the former not only drives collagen synthesis but also directly stimulates osteogenesis and chondrogenesis by acting on progenitor cells.^[^ ^149^ ^]^ This establishes a positive feedback loop wherein piezoelectric stimulation induces a healing phenotype that, in turn, improves extracellular matrix quality and organization. The net effect is a coupling of mechanical, electrical, and immune signals: piezo‐driven M2 macrophages help regulate collagen assembly, ensure a proper degradation‐synthesis balance via matrix metalloproteinases and TIMPs, and promote a well‐vascularized, innervated tissue bed conducive to regeneration.^[^ ^150^ ^]^ Such immune modulation is emerging as a key advantage of piezoelectric materials, linking mechanotransduction with the tissue‐healing machinery.

### 5.3 Translational Implications and Controversies

Harnessing collagen dynamics and macrophage polarization in clinical practice demands precise control over these bioresponses. Regarding collagen, bioengineers must rigorously monitor the activation of the integrin/TGF‐β/collagen positive feedback loop—a mechanism that has been extensively addressed in preceding sections and therefore will not be elaborated upon here.

And with macrophages, the situation gets even more interesting. Steering macrophages to the “pro‐healing” M2 state has immense benefits, but this is not always the case. M2 macrophages are not a monolithic entity; they encompass subtypes (M2a, M2b, M2c, etc.) with nuanced functions.^[^ ^151^ ^]^ In tissue engineering, encouraging an anti‐inflammatory M2‐dominant response can improve regeneration, but imbalances in macrophage activation can have unintended consequences. One concern is that an overly M2‐skewed response might suppress necessary early immune functions—for example, if the material drives macrophages to heal too quickly, the clearance of pathogens or debris (normally handled by M1 activity) might be incomplete, risking infection or poor integration.^[^ ^152^ ^]^ Another issue is the potential for fibrotic M2 phenotypes: certain M2 (especially those stimulated by IL‐13/IL‐4, termed M2a) can promote fibrogenesis by releasing TGF‐β and PDGF, leading to myofibroblast recruitment.^[^ ^153^ ^]^ In fact, macrophages present in late‐stage fibrosis often resemble M2 cells—they produce high levels of collagen‐stimulating factors and contribute to persistent scar tissue.^[^ ^144^ ^]^ This dual nature of M2 (“friend and foe” in fibrosis) underscores that simply boosting M2 is not universally beneficial. In some contexts, M2‐like tumor‐associated macrophages can even aid tumor growth and suppress immune surveillance—an extreme reminder that pro‐regenerative macrophages can have downsides if misdirected.^[^ ^152^ ^]^ In the context of biomaterials, there is the FBR to consider: macrophages responding to an implant can fuse into giant cells and orchestrate fibrous capsule formation.^[^ ^154^ ^]^ This FBR is often associated with a frustrated healing attempt involving cytokines from both M1 and M2 spectra. Designing piezoelectric materials that avoid chronic activation of macrophages is therefore critical. It remains a challenge to achieve the right macrophage activation at the right time—a too‐strong push toward M2 might inadvertently shortcut the normal healing sequence or create an immunosuppressive niche, whereas insufficient M2 activation could result in unresolved inflammation and tissue damage.

In summary, the translational use of piezoelectric scaffolds holds great promise due to their ability to merge mechanical and biochemical healing cues. However, it also demands a precision medicine approach—tailoring the material's properties and stimulation protocol to strike a balance between regenerative efficacy and the avoidance of unintended fibrosis or immune reactions. Section 6 will build upon these mechanistic insights by examining concrete applications in regenerative medicine—from bone grafts that exploit piezoelectric stimulation to enhance osteogenesis, to cartilage repair constructs that restore hyaline matrix, wound healing platforms that reduce scarring, and so on—illustrating how collagen and immune modulation translate into improved outcomes.

## 6 Current State of Piezoelectric Materials for Tissue Regeneration Applications

Piezoelectric materials have been used in a wide range of applications in the field of tissue regeneration, including regeneration of bone, cartilage, peripheral nerves, central nervous system, wounds, dental tissues, tendons, myocardium, and cornea. Here, we will present the important results in the last five years and summarize the important information in **Tables** 3, 4, 5, 6, 7. It should be stated that Tables 3, 4, 5, 6, 7 is located at the end of the section.

**Table 3:** Application of piezoelectric materials in bone regeneration. AESO, acrylate epoxidized soybean oil; ATP, Ag/3‐(trimethoxysilyl) propyl methacrylate/pBT; BT, BaTiO_3_; BTO, BaTiO_3_; BV, bone volume; C‐V curve, capacitance‐voltage curve; CG, chitosan/gelatin; CM, compressive modulus; CMBT, Ca/Mn co‐doped BaTiO_3_; CS, compressive strength; EABP, ECM/AlgMA/PDA‐modified black phosphorus nanosheets; EL, elongation; EM, elastic modulus; FCD, femoral condylar defect; HA, hydroxyapatite; HTP‐NG, hybrid tribo/piezoelectric nanogenerator; KBTO, (KH‐550)‐modified BaTiO_3_ Nanoparticles; KNN, KNaNbO_3_; OC, output current; OCS, oxidized chondroitin sulfate; OV, output voltage; PBT, polydopamine‐modified barium titanate; PCL, polycaprolactone; PD, poly(ethylene dioxythiophene)/polystyrene sulfonate; PDA, dispersing dopamine; PHA, polydopamine‐modified hydroxyapatite; PHB, poly[(R)3‐hydroxybutyrate]; PHBV, poly[3‐hydroxybutyrate‐co‐3‐hydroxyvalerate]; PLA, polylactic acid; PLLA, poly‐L‐lactic acid; PVDF, polyvinylidene fluoride; PWH, piezoelectric whitlockite; SM, storage modulus; SP, surface potential; T2DM, type II diabetes mellitus; TCP, tricalcium phosphate; TS, tensile strength; TV, total volume; VAuHpl@rGO, Au nanodots, nanoHA, and vancomycin‐modified graphene oxide sheets.

Model	Piezoelectric material	Mechanical performance	Piezoelectric performance	Activation	Regenerative contribution	Refs.
Mice, cranial defect (3.5 mm)	PLLA nanofiber mat	∖	OV 28mV	Ultrasound	Higher 10‐day ALP and mineralization	[156]
Mice, cranial defect (5 mm)	HA/PDA/PLLA scaffold	EM 5.4 GPa	d_14_ 1.82 pC N^−1^, OV 190 mV	Ultrasound	94.4% bone coverage at 12 weeks	[157]
Rats, cranial defect (5 mm)	PBT/PHA/PHBV bionic periosteum	TS 19–23 MPa	OV 11.2 V, OC 4.3 µA	Traction	60.8% BV/TV at 8 weeks	[158a]
∖	CaCO3‐PHB and ‐PHBV scaffold	∖	d_33_: PHB 3 pC N^−1^, PHBV 0.7 pC N^−1^	Traction	2 × Mineral Boost after 90 s US	[158b]
Rats, cranial defect (5 mm)	BT/PLA membrane	∖	d_33_ 4.5–7 pC N^−1^	Traction	90% bone regeneration at 12 weeks	[159a]
Rabbits, radial defect (15 mm)	TCP‐PLA/GeSe scaffold	∖	OV 7.5 V, OC 22 µA	Ultrasound	Around 40% BV/TV at 12 weeks	[159b]
Rabbits, radial defect (13 mm)	Porous BT/Ti_6_Al_4_V scaffold	EM 2.8±0.3 GPa, CS 63±6 MPa	∖	Ultrasound	Around 20% BV/TV at 12 weeks	[160a]
Sheep, spinal fusion (12,6 mm)	Porous BT/Ti_6_Al_4_V scaffold	EM 2.8±0.3 GPa, CS 63±6 MPa	d_33_ 0.7 pC N^−1^	Movement	58.68% bone coverage at 8 months	[160b]
Sheep, C4 vertebral corpectomy	Porous BT/Ti_6_Al_4_V scaffold	∖	d_33_ 3 pC N^−1^	Ultrasound	33.97% BV/TV at 12 months	[160c]
∖	BT/β‐TCP ceramic	∖	OV 590 mV, OC 300 nA	Traction	2.5‐fold intracellular Ca^2+^ at 6 hours	[161a]
Rats, cranial defect (5 mm)	Ti/PVTF/Ti sheet	∖	d_33_ 0–8 pC N^−1^, SP 10–80 mV	Traction	1.7‐3.8‐fold BV and surface at 8 weeks	[161b]
Rats, cranial defect (5 mm)	Mg^2+^‐releasing PWH/PCL scaffold	EL 35%, TS 14 MPa	CV curve redox peak at 0.8 V	Traction	17.53% BV/TV at 8 weeks	[162a]
Rats, cranial defect	Shape‐memory AESO/ATP scaffold	CS 1.4‐2.2 MPa, EL>20%	d_33_ 0.1–1.4 pC N^−1^	Traction	Covered by new bone at 8 weeks	[162b]
T2DM Rats, 5 mm cranial defect	PCL/KNN@PDA film	∖	OV 4.4 V, OC 20 µA	Ultrasound	62.5% BV/TV at 8 weeks	[162c]
Rats, cranial defect	CG/PHA/PBT hydrogel	SM 493‐432 Pa	OV 0.6–0.8 V	Traction	35.4% BV/TV at 8 weeks	[164a]
Rats, cranial defect (5 mm)	OCS/Gel/KBTO hydrogel	SM 2407–2560 Pa	OV −41.6–61.82 mV	Ultrasound	Around 25% BV/TV at 12 weeks	[164b]
Rats, cranial defect (5 mm)	Gel‐PD‐CMBT hydrogel	Strain 40%, CM (40%) 0.6 KPa	Large CV curve area, low impedance	Traction	53.38% BV/TV at 8 weeks	[164c]
Rats, FCD (3 mm)	EABP/HTP‐NG composite device	EABP Shear stress 196 Pa	HTP‐NG OV 35 V, OC 3.7 µA	Movement	1.4‐fold BV/TV at 6 weeks	[165]
Rats, cranial defect	VAuHp@rGO bone cement	Flexural strength 19–25 MPa	OV 10–123 mV, OC 0.4 µA cm^−2^	Traction	25% BV/TV at 11 days	[167a]
Rabbits, FCD (6*6*2 mm)	PMMA/BT bone cement	CS>70 MPa	d_33_ 90.1 pC N^−1^, OV 37.109 V	Movement	4.3‐fold new BV at 2 months	[167b]
Rats, FCD or diaphysis defect	PMMA/PEI/PVDF bone cement	Femoral flexural strength 99 MPa	OV 50–200 mV	Movement	Better bone growth at 12 weeks	[167c]
Rats, infectious FCD (1.5*3 mm)	Se@BTO nanoparticle	∖	d_33_ 11 pm V^−1^	Ultrasound	Around 40% BV/TV at 28 days	[163]

**Table 4:** Application of piezoelectric materials in cartilage regeneration. ADSCs, adipose‐derived stem cells; BMSCs, bone marrow mesenchymal stem cells; BT, BaTiO3; CM, compressive modulus; EM, elastic modulus; GO, graphene oxide; ICRS, international cartilage repair society (scores system); OV, output voltage; PLLA, poly‐L‐lactic acid; PVA, poly(vinyl alcohol); PVDF, polyvinylidene fluoride.

Model	Piezoelectric material	Mechanical performance	Piezoelectric performance	Activation	Regenerative contribution	Refs.
BMSCs	PLLA scaffold	EM 10.5–12.1 MPa	OV 1.03‐6.87 V	Traction	Osteochondral differentiation at 3 days	[168a]
ADSCs	BT/GO/VitroGel‐RGD hydrogel	CM 2 KPa	OV 43.1 µV	Ultrasound	Chondrogenic differentiation at 10 days	[168b]
Rabbits, 4 × 5 mm cartilage defect	PVA/PVDF/Ag hydrogel	CM 0.07–0.7 MPa	OV 0.32‐0.36 V	Movement	10 ICRS, 22 histological scores at 12 weeks	[169a]
Rabbits, 4 × 2 mm cartilage defect	PLLA/collagen scaffold	EM 642 MPa	OV 3.6 V	Movement	11 ICRS, 15 histological scores at 2 months	[169b]
Rabbits, 4 × 2 mm cartilage defect	PLLA/collagen hydrogel	Shear‐thinning	Dry OV 33.7 mV	Ultrasound	11 ICRS, 15 histological scores at 2 months	[169c]

**Table 5:** Applications of piezoelectric materials in peripheral nerve regeneration and central nervous system repair. BBB, Basso Beattie‐Bresnahan (scores system); BT, BaTiO_3_; CS, compressive strength; EM, elastic modulus; F‐VEP, flash visual evoked potential; KNN, KNaNbO_3_; MAPbBr_3_, methylammonium lead bromide perovskite; MN, micro‐needle; NP‐NGC, nanoporous nerve guide conduit; OC, output current; OV, output voltage; P(VDF‐TrFE), polyvinylidene fluoride‐trifluoro ethylene; P3EPT‐FA, folate‐azide‐functionalized poly(ethyl 5‐(thiophen‐3‐yl)pentanoate‐2,5‐diyl); PCBM, [6,6]‐phenyl‐C61‐butyric acid methyl ester; PDA, dispersing dopamine; PLA, polylactic acid; PLLA, poly‐L‐lactic acid; PPy, polypyrrole; PRG‐G‐C, CXCL12 and ginseng‐derived exosome‐like vesicles loaded PVDF/rGO/Gel; PVDF‐HFP, poly(vinylidene fluoride‐co‐hexafluoropropylene); PVDF‐TrFE‐CFE, poly(vinylidene fluoride‐trifluoroethylene‐chlorofluoroethylene); PZG, PCL/ZnO NPs/rGO; RCS, Royal College of Surgeons; SF, silk fibroin; SFI, Sciatic nerve function index; TP‐hNG, tribo/piezoelectric hybrid nanogenerator; TS, tensile strength.

Model	Piezoelectric material	Mechanical performance	Piezoelectric performance	Activation	Regenerative contribution	Refs.
Rats, 10 mm sciatic nerve defect	SF/PVDF‐HFP/MXene conduit	TS 5.33‐9.03 MPa	OV 23–100 mV	Movement	−33.3 SFI at 12 weeks	[171a]
Rats, 15 mm sciatic nerve defect	P(VDF‐TrFE) conduit	∖	d_33_ 15–45 pm V^−1^, OV 100–400 mV	Shockwave	−25 SFI at 80 days	[171b]
Rats, 10 mm sciatic nerve defect	BT/P(VDF‐TrFE) hydrogel conduit	TS 7.5‐20 MPa	OV 4.9–10.5 V, water 1.25–1.75 V	Ultrasound	Around −55 SFI at 8 weeks	[171c]
Rats, 10 mm sciatic nerve defect	PPy/PDA/PLLA conduit	EM 282 MPa, TS 16 MPa	OV −750 to 500 µV	Traction	−57.12 SFI at 12 weeks	[172]
Rats, 15 mm sciatic nerve defect	TP‐hNG/NP‐NGC composite device	NP‐NGC EM 72.52±0.5 MPa	TP‐hNG 16–32 V cm^−2^	Chest movement	Around −38 SFI at 12 weeks	[173a]
Rats, 15 × 15 mm skin full‐excision	PRG‐G‐C skin patch	TS 10 MPa	OV 35–350 mV	Traction	2.8–5.6‐fold scratching bout rate	[173b]
Rats, 10 mm sciatic nerve defect	Topological PZG conduit with MNs	EM 1.12 MPa, CS 400 N	OV 0.89–5.84 V, OC 0.63–2.41 µA	Traction	80.56% muscle fiber density (8 weeks)	[173c]
Rats, T9 spinal cord injury	PLA/KNN@PDA 3D scaffold	EM 25 N/mm^2^	d_33_ 20 pC N^−1^, OV 12 V, OC 20.8 µA	Ultrasound	Around 15 BBB scores at 8 weeks	[176a]
Rats, spinal cord injury	Au@BT nanoparticle	∖	Piezo‐catalytic efficiency	Ultrasound	Over 14 BBB scores at 28 days	[176b]
PC12 cells	P(VDF‐TrFE)/P(8‐AZO‐10)	Good flexibility	Maximum capacitance change (under blue light)	Isomerization	25 000 PPI resolution	[174c]
Rats, dystrophic RCS	P(VDF‐TrFE)/P3EPT‐FA/PCBM	Good flexibility	Maximum voltage change (under blue light)	Isomerization	F‐VEP 75.75 µV at 3 months	[174a]
∖	PVDF‐TrFE‐CFE/MAPbBr3	Good flexibility	Maximum capacitance change (under yellow‐green light)	Isomerization	20 ms response time	[174b]

**Table 6:** Application of piezoelectric materials in wound healing. BPV, PTEN inhibitor; BT, BaTiO_3_; BTO, BaTiO_3_; CIP, ciprofloxacin; CM, compressive modulus; CMZ, carbonized moxa@ZnO; CS, compressive strength; EL, elongation; EM, elastic modulus; MA, micropatterned/aligned; OC, output current; OV, output voltage; PCP, polyacrylamide/carboxymethyl chitosan (CMCS)/Ppy; PDMAA, poly(N, N‐dimethylacrylamide); PEGDA, poly (ethylene glycol) diacrylate; PENG, piezoelectric nanogenerator; PLLA, poly‐L‐lactic acid; PTFE, polytetrafluoroethylene; PVA, poly(vinyl alcohol); PVDF, polyvinylidene fluoride; SA, sodium alginate; SF, silk fibroin; SM, storage modulus; T1DM, type Ι diabetes mellitus; TS, tensile strength; ZIF‐8, zeolitic imidazolate framework‐8.

Model	Piezoelectric material	Mechanical performance	Piezoelectric performance	Activation	Regenerative contribution	Refs.
Mice, 10 mm infected wound	BTO@ZIF‐8/CIP nanoparticle	∖	Piezopotentials −2.8–0.8V	Ultrasound	99.3% wound healing at 11 days	[178a]
Mice, 10 mm infected wound	SF‐MA/PEGDA/Ag@BT hydrogel	EL>38%, CM 0.17 KPa‐83.1 MPa	Butterfly amplitude and phase curves	Ultrasound	99.9% wound closure at 12 days	[178b]
Rats, T1DM, 10 mm wound	PVA/PVDF hydrogel	EL 300%, TS 0.5‐6.5 MPa	d_33_ 0.5–8.4 pC N^−1^, OV 0.9–2.8 V, OC 104–248 nA	Traction	New epithelium closure at 10 days	[179a]
Mice, skin scald wound (5‐15 s)	PDMAA/PVDF hydrogel	TS 15–25 KPa, EM 3–5 KPa, Shear stress 13–21 KPa, adhesion strength 20 KPa	OV 50 mV, OC 20–150 nA	Massager	Almost complete repair at 18 days	[179b]
Rats, 10 × 10 mm wound	ZnO/PVDF/SA scaffold	EL 33%, TS 1415 KPa	OV ±200 mV, OC ±1.29 µA	Traction	Complete wound closure at 14 days	[180]
Rats, 7 × 4 mm oral mucosal defect	PTFE vs P(VDF‐TeFE) film	TS 2.3/7.1 MPa, EL 324%/215%	d_33_ 0.48/2.13 pm V^−1^	Traction	12‐day healing with 1.5 mm^2^ scar	[181]
Rats, infected full‐thickness wound	BTO‐Au/PEGDA/GelMA patch	∖	Piezo‐catalytic efficiency	Ultrasound	Best effect at 10 days	[182a]
Mice, full‐thickness wound	PLLA scaffold	TS 12.16 MPa, SM 616 MPa	OV 300 mV	Ultrasound	2.74 mm^2^/day healing rate	[182b]
Rats, full‐thickness wound	BPV@PCP/PENG composite	Hydrogel TS 0.15 KPa, CS 80 KPa	PENG OV 420–2630 mV	Movement	Complete wound closure at 16 days	[182c]
Rats, fungal infected wound	Carbonized moxa@ZnO nanosheet	∖	Piezoelectric photocatalytic stability	Ultrasound	Wound closure at 8 days, abscess ablation at 7 days	[182d]
Rats, full‐thickness wound	γ‐glycine/PVA composite device	EL 30–90%, TS 5–15 MPa	d_33_ 1.4–10.4 pC N^−1^, g_33_ (50‐324) × 10^−3^ Vm N^−1^	Ultrasound	Accelerated wound healing by 40%	[182e]

**Table 7:** Application of piezoelectric materials in the regeneration of other tissues. BPCL, blink‐driven piezoelectric contact lens; BT, BaTiO_3_; BV, bone volume; CS, compressive strength; EL, elongation; EM, elastic modulus; GO, graphene oxide; LM, loss modulus; OV, output voltage; P(VDF‐TrFE), polyvinylidene fluoride‐trifluoro ethylene; PCL, polycaprolactone; PETRR, a novel piezoelectric elastomer for tendon rupture regeneration and monitoring; PVDF, polyvinylidene fluoride; SM, storage modulus; TS, tensile strength; TV, total volume; UVECs, umbilical vein endothelial cells.

Tissue	Model	Piezoelectric material	Mechanical performance	Piezoelectric performance	Activation	Regenerative contribution	Refs.
Dental	Mice, periodontal defect	BT/GelMA hydrogel	SM 10 KPa, LM 1 KPa	OV 10 mV mm^−2^	Movement	0.2 mm pocket depth at 1 month	[183a]
Dental	Rats, periodontal defect	t‐BT/GelMA hydrogel	CS 20–45 KPa, EM 10–15 KPa	OV 33–113 mV	Movement	91.45% BV/TV at 12 weeks	[183b]
Dental	Rats, mandibular defect	Polarized P(VDF‐TrFE) Janus film	TS 30 MPa, EM 1200 MPa	d_33_ 10 pC N^−1^	Movement	Almost completely restored at 8 weeks	[184]
Dental	Dogs, dentin defect	SrCl_2_/P(VDF‐TrFE) film	TS 0.57 MPa, ES 0.62 MPa	d_33_ 14 pC N^−1^	Movement	New dentin formation at 3 months	[185]
Tendon	Rats, Achilles tendon injury (5 mm removed)	P(VDF‐TrFE) scaffold	EM 62 MPa, TS 31 MPa, EL 39%	d_33_ 36.5 pC N^−1^	Movement	1.25–1.75‐fold joint function at 8 weeks	[186a]
Tendon	Rats, Achilles tendon injury	PETERR elastomer	TS 0.9–1 MPa, EL 425–450%	OV 0.13–0.43 V	Movement	Around 15 Stoll scores at 8 weeks	[186b]
Myocardial	Human UVECs	PVDF/GO patch	EM 2.5–10.4 MPa, EL 97–119%	OV 1.5–9.4 V	Traction	3.4‐fold cell proliferation at 7 days	[189]
Myocardial	Rats, myocardial infarction	PCL/PVDF scaffold	TS 0.6–0.8 MPa	OV in vivo 0.5–1 mV	Movement	123% ejection fraction at 28 days	[187]
Corneal	Mice and Rabbits, corneal alkali burn	BPCL contact lens	EL 40%	OV 2.9 V	Movement	Higher repair rate (96%, 6 days, mice; 90%, 8 days, rabbit)	[188]

### 6.1 Bone Regeneration

The largest application of piezoelectric materials is in the field of bone repair. As early as 1997, Feng et al. implanted piezoelectric ceramics synthesized from HA and BT into the jawbones of dogs, generating ES with the help of the dog's chewing action, thus promoting bone growth.^[^ ^155^ ^]^ Significant advances have been made in the field of piezoelectric materials for bone regeneration in the last five years, which utilize mechano‐electrical conversion to enhance osteogenesis through different platforms such as scaffolds, hydrogels, and bone cement. The common philosophy across studies emphasizes mimicking the natural piezoelectricity of bone, and materials such as PLLA scaffolds, BaTiO_3_/Ti_6_Al_4_V composites, and PVDF‐based hydrogels can be used to generate electrical stimulation through ultrasound or physiological exercise, promoting calcium influx, angiogenesis, and M2 macrophage polarization. The main results are summarized in Table 3.

#### 6.1.1 Piezoelectric Scaffold in Bone Regeneration

**Figure 5:**
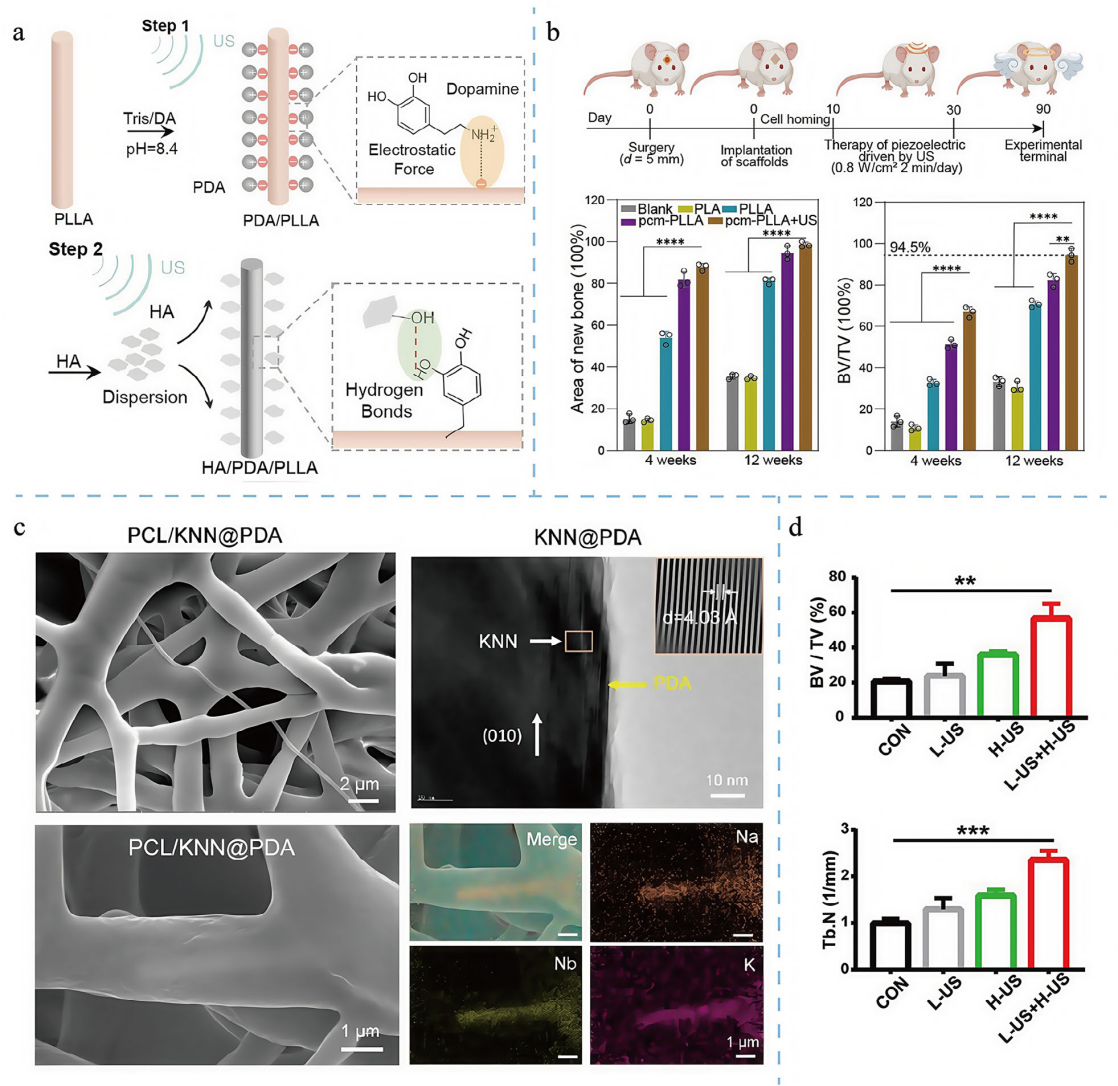
The cases for piezoelectric materials for bone regeneration. a) Structure of the mineralized PLLA scaffolds. b) Mineralized PLLA scaffolds promote bone regeneration in vivo. Reproduced with permission.^[^ ^157^ ^]^ Copyright 2024, Elsevier. c) Structure of the PCL/KNN@PDA films. d) PCL/KNN@PDA films promote bone regeneration in diabetic mice. Reproduced with permission.^[^ ^162c^ ^]^ Copyright 2023, American Chemical Society.

Polylactic acid (PLA) is a non‐centrosymmetric polymer, of which PLLA has been highlighted for its high shear piezoelectric coefficient d_14_ (10 pC N^−1^).^[^ ^6^ ^]^ Das et al. produced PLLA nanofiber mats by electrostatic spinning.^[^ ^156^ ^]^ A 40 kHz commercial ultrasonic water bath was used to measure the output voltage (OV), which resulted in ≈28 mV. Mice with cranial defects implanted with scaffolds were subjected to 30 min, 40 kHz US treatment every day, 5 days a week for 4 weeks. The results showed that scaffolds prepared at 3000 rpm had stronger OV under the US than those prepared at 1000 rpm and 300 rpm. It is evident that innovations in the preparation processes of materials have a significant impact on improving material properties. However, since many property (electrical stimulation)–mechanism (regeneration) pathways are still in the process of verification, the bridge between materials–efficacy–clinical application has not been fully established, despite the emergence of many promising studies. What we are currently doing is summarizing the points of inspiration from various studies and piecing these hopes together to form the outline of a bridge, thereby reflecting the current state of progress in the field. We believe that future research can gradually solidify the foundation of this bridge, ultimately allowing patients to step onto the bridge of healing. BMSCs showed enhanced osteogenic differentiation, assessed by increased alkaline phosphatase (ALP) activity, elevated osteocalcin and osterix gene expression, and greater calcium deposition quantified by alizarin red staining.^[^ ^156^ ^]^ Cui et al. prepared PLLA scaffolds with mineralized layers based on electrostatically spun PLLA scaffolds, which were sequentially covered with polydopamine (PDA) and HA coatings by water bath sonication (**Figure** **5**a).^[^ ^157^ ^]^ Considering that HA thickness is positively correlated with Young's modulus and negatively correlated with piezoelectric properties, they chose a moderate thickness (d_14_ = 1.82 pC N^−1^, OV = 190 mV) for the rat skull defect repair experiments. US with an intensity of 0.8 W cm^−2^ and a duration of 1 min was applied twice daily. In vitro experiments have shown that US piezoelectric signals increase calcium inward flow. In vivo experiments suggest that scaffold osteogenesis may occur in a manner related to the promotion of endogenous stem cell recruitment, angiogenesis, and macrophage M2 polarization, with 94.5% BV/TV at 12 weeks (Figure 5b).^[^ ^157^ ^]^ Liu et al. and Chernozem et al. conducted a similar study in which mineralized scaffolds were prepared to mimic the bone tissue microenvironment.^[^ ^158^ ^]^ Liu et al. composited polydopamine‐modified HA and barium titanate (PHA and PBT) onto poly[3‐hydroxybutyrate‐co‐3‐hydroxyvalerate] (PHBV) polymer matrix.^[^ ^158a^ ^]^ Chernozem et al.prepared scaffolds poly[(R)3‐hydroxybutyrate] (PHB) and PHBV with surface coated CaCO_3_.^[^ ^158b^ ^]^ Xu et al. and Dai et al. combined PLA with tricalcium phosphate (TCP)/Germanium Selenium (GeSe) and BT, respectively, to improve the piezoelectric and osteogenic properties of PLA.^[^ ^159^ ^]^ Among them, the TCP‐PLA/GeSe scaffold has dual piezoelectric and photothermal properties and is suitable for osteosarcoma removal and bone remodeling.^[^ ^159b^ ^]^

Guo et al. have published several studies on BaTiO_3_/Ti_6_Al_4_V scaffolds.^[^ ^160^ ^]^ They covered the surface of the porous titanium scaffold with a BT coating to mimic the mechanical and electrophysiological properties of natural bone. The elastic modulus (EM) of the composite stent was 2.8 ± 0.3 GPa, and the compressive strength (CS) was 63 ± 6 MPa.^[^ ^160a^ ^]^ The d_33_ of the stent after corona polarization (11.5 kV, 30 min) was 3.2 pC N^−1^, and it was able to generate a subcutaneous current of 6.7 µA.^[^ ^160c^ ^]^ They have implanted scaffolds in a rabbit radial defect model and a sheep cervical discectomy model, supplemented with low‐intensity pulsed US (frequency 1.5 MHz, duration 0.2 ms, strength 30 mW cm^−2^, repetition frequency 1 kHz), and achieved bong volume (BV)/total volume (TV) values of ≈20–33.97%.^[^ ^160a,c^ ^]^ Specific mechanisms may be the promotion of BMSCs activity,^[^ ^160a^ ^]^ the promotion of M2 polarization and immunomodulation in macrophages,^[^ ^160c^ ^]^ and the promotion of angiogenesis.^[^ ^160b^ ^]^ It is important to note that one of the studies utilized forces generated by the physiological motion of the cervical spine to excite the piezoelectric scaffolds in the cervical fusion model but did not compare this to the US mode of excitation.^[^ ^160b^ ^]^ The optimal excitation method remains controversial, with ultrasound‐driven systems providing precise control but lacking utility compared to motion‐driven methods, which remain underexplored in one‐to‐one comparisons. Nonetheless, evidence from this study supports the potential for native biomechanics to generate therapeutic electrical cues.^[^ ^160b^ ^]^ For instance, finite element analysis simulating physiological loading (a 400 N load) in the cervical fusion model predicted maximum elastic strains within the implant cage itself reaching 1.25 × 10^−3^. This deformation of the piezoelectric BaTiO_3_ coating predicted under physiological strain indeed generates functionally relevant local electrical signals, that is, the polarized scaffold exhibited a measured surface potential of ≈−30 mV, a value situated within ranges reported in other studies as being osteoinductive.^[^ ^160b^ ^]^ Correspondingly, in vivo experiments under physiological loading demonstrated that this polarized scaffold yielded superior osteogenesis and angiogenesis compared to non‐piezoelectric controls, highlighting a tangible biological benefit attributable to the motion‐induced electrical stimulation originating from the strained material.^[^ ^160b^ ^]^

However, whether the native biomechanical environment consistently provides sufficient stimulus to achieve therapeutic effects across different materials and anatomical locations remains an open question. The magnitude of the generated electrical signal is directly dependent on the applied stress/strain and the material's piezoelectric coefficients and mechanical properties. Achieving adequate strain within the implant necessitates not only sufficient external loading, dictated by patient activity levels, but also effective mechanical coupling and load transfer through appropriate scaffold design, avoiding stress shielding. Furthermore, the inherent variability in piezoelectric properties among different materials and even within the same material class due to processing differences, coupled with the challenge of precisely matching the generated localized, often transient, electrical signals to the effective parameters derived from sustained external stimulation studies, complicates predictions of therapeutic efficacy. Therefore, while physiological motion presents an appealing, power‐free activation strategy, its success likely hinges on meticulous optimization of material selection, scaffold architecture, implantation site, and consideration of the anticipated patient‐specific loading patterns. Further comparative studies evaluating motion‐driven versus external excitation modalities are warranted to fully elucidate their relative effectiveness.

Another very important point is that even though BV/TV values, also named as the bone volume fraction, are used in many osteogenesis studies,^[^ ^158^, ^159^, ^160^ ^]^ their quality control bias from study to study is a gulf in front of clinical translation. BV/TV measurements are mainly performed by µCT, and variations in µCT parameters, including resolution, threshold, and region of interest (ROI), are key to making results difficult to compare. Different thresholds can vary results by as much as 85%.^[^ ^6^ ^]^ And it is not surprising that different regions of interest have different results depending on the reference region referred to (either the interface or the entire implant region, and the defined volume). Liu et al. (rat cranial defects) scanned with SkyScan 1276 with an ROI of 5 mm × 1.5 mm, but no threshold was specified.^[^ ^158a^ ^]^ Xu et al. (rabbit radius) measured BV/TV with micro‐CT without defining the ROI or software.^[^ ^159b^ ^]^ Fan et al. (rabbit radius) specified 13 µm resolution and 300–1300 threshold, with an ROI of 5 mm × 13 mm,^[^ ^160a^ ^]^ while Wu et al. (sheep cervical spine) used similar equipment but with an ambiguous ROI.^[^ ^160c^ ^]^ Clearly, there is no standardization between these excellent studies. Future studies could focus on developing rigorous µCT scanning procedures in the context of biomaterials (including resolution, threshold settings, and ROI definitions), encouraging studies that are more transparent in their descriptions of µCT parameters, and reducing the variability associated with traditional image analysis with the visual capabilities of today's artificial intelligence. These efforts will improve the reliability of BV/TV as a metric, thus providing the possibility of comparison between different biomaterials and advancing the field of bone tissue regeneration.

The surface potential (SP) of piezoelectric scaffolds is closely related to the cellular state and is influenced by the polarized electric field, material deformation (locomotion state of animals), and preparation process.^[^ ^161^ ^]^ Mao et al. and Lin et al. quantitatively controlled the SP of the material.^[^ ^161^ ^]^ Mao et al. prepared BT/β‐TCP (BTCP) piezoelectric ceramic and optimized d_33_ (6–80 pC N^−1^) by increasing the sintering temperature, decreasing the sintering rate, and controlling the direction of the polarization electric field to tune the SP (+18.5 mV, +107 mV, and −175 mV). The results showed that SP with negative potentials increased fibronectin adsorption as well as extracellular calcium ion inward flow, and SP with positive potentials may be associated with the anti‐inflammatory phenotype of macrophages.^[^ ^161a^ ^]^ Lin et al. prepared Ti/PVDF/Ti sheets with controlled surface potential by sandwiching PVDF between Ti foils. The results show that the d_33_ of the metal surface is 0–8 pC N^−1^ at different field strengths, and its SP is related to the thickness and deformation of the sheets. In vivo, SP was correlated with rat locomotion and was 10 mV at rest and 80 mV when active. SP may promote osteogenesis by activating VGCC and calcium ion signaling pathways.^[^ ^161b^ ^]^

In addition to the above piezoelectric scaffolds, some novel scaffolds have also been reported.^[^ ^162^ ^]^ Whitlockite (WH), a magnesium‐containing calcium phosphate with piezoelectric properties and sustained release of magnesium ions after sintering, is an optimized alternative to HA and was used by Wang et al. for the preparation of piezoelectric WH (PWH)/PCL composite scaffolds.^[^ ^162a^ ^]^ Annealing at 650 °C for 3 h was used to induce WH to PWH. The PWH composite scaffolds had better mechanical and piezoelectric properties compared to WH and β‐TCP. Analysis of the expression of the neurogenic genes nestin, TUBB3, and NEFL in BMSCs showed that Mg^2+^ promoted neurogenesis more strongly compared to Ca^2+^ and that PWH was stronger compared to WH. The chicken embryo allantoic membrane model and rat cranial defect models were used to evaluate the angiogenic and osteogenic capacities of the scaffolds, respectively. The results showed that the expression of Ang‐1 and VEGF genes, ALP activity, and COL‐I synthesis were superior in the PWH scaffold group compared to the WH, β‐TCP, and PCL scaffolds, with a bone volume fraction of 17.53% and a mineral density of 288.07 mg cm^−3^ at 8 weeks.^[^ ^162a^ ^]^ Li et al. used 3‐(trimethoxysilyl) propyl methacrylate (TMSPM) and Ag NPs to modify BT NPs with PDA coating (pBT) to obtain Ag‐TMSPM‐pBT (ATP) NPs. ATP NPs were further loaded into acrylate epoxidized soybean oil (AESO), a shape memory polymer.^[^ ^162b^ ^]^ The results showed that the mechanical properties (compressive strength, compressive modulus, and maximum strain) of the composite scaffolds diminish with the addition of ATP NPs. AESO‐10% ATP was used for the repair of cranial defects in rats with a polarized d_33_ of 0.9 pC N^−1^ and its maximum output current (OC) of 146.4 nA, which had good osteogenic properties and almost restored the initial shape at 45 °C for 60 s.^[^ ^162b^ ^]^ Sun et al. prepared PDA‐modified KNN nanowires and synthesized US‐responsive piezoelectric membrane PCL/KNN@PDA together with PCL (Figure 5c).^[^ ^162c^ ^]^ Its underwater OV and OC under specific ultrasonic conditions (frequency 100 kHz, width 50 µs, interval 10 ms, intensity 0.7 W cm^−2^) were 4.4 V and 20 µA, respectively. Notably, they prepared a rat model of type 2 diabetes mellitus (T2DM) on which they fabricated cranial defects to explore the bone regenerative capacity of piezoelectric films in the context of diabetes. Biocompatibility experiments showed that both low (5 min) and high (20 min) doses of US (100 kHz, 100 KPa acoustic pressure) had no effect on cellular activity and were further used in the following experiments. The results showed that US‐excited piezoelectric membranes could induce M2 polarization of macrophages in high glucose environments, increasing CD206^+^ cells to ≈40% via the AKT2‐IRF5/HIF1α pathway with reduced AKT2 phosphorylation, enhance BMSC recruitment with higher CD44^+^ cell presence and migration efficiency under low‐dose US, and promote osteogenesis with elevated ALP, COL1, OSX, and RUNX2 expression, achieving a bone volume fraction of 62.5% at 8 weeks under combined US treatment (Figure 5d).^[^ ^162c^ ^]^

Importantly, existing studies are mired in effect validation, with weaknesses in the lack of standardized means of comparison (assessment of means such as inconsistency in BV/TV, differences in baseline regenerative capacity in different animals), difficulty in evaluating inconsistencies in reporting of piezoelectric outputs (e.g., 28 mV^[^ ^156^ ^]^ vs 4.4 V^[^ ^162c^ ^]^), and limited translational data of relevance to humans, especially for complex situations such as diabetes^[^ ^162c^ ^]^ or complex situations such as infections.^[^ ^163^ ^]^ Future research should prioritize transparent, standardized, and rigorous evaluation processes that focus on the integration of long‐term biocompatibility and efficacy to bridge the translational gap and ensure that these innovations are applied beyond animal models to orthopedic practice.

#### 6.1.2 Piezoelectric Hydrogel in Bone Regeneration

**Figure 6:**
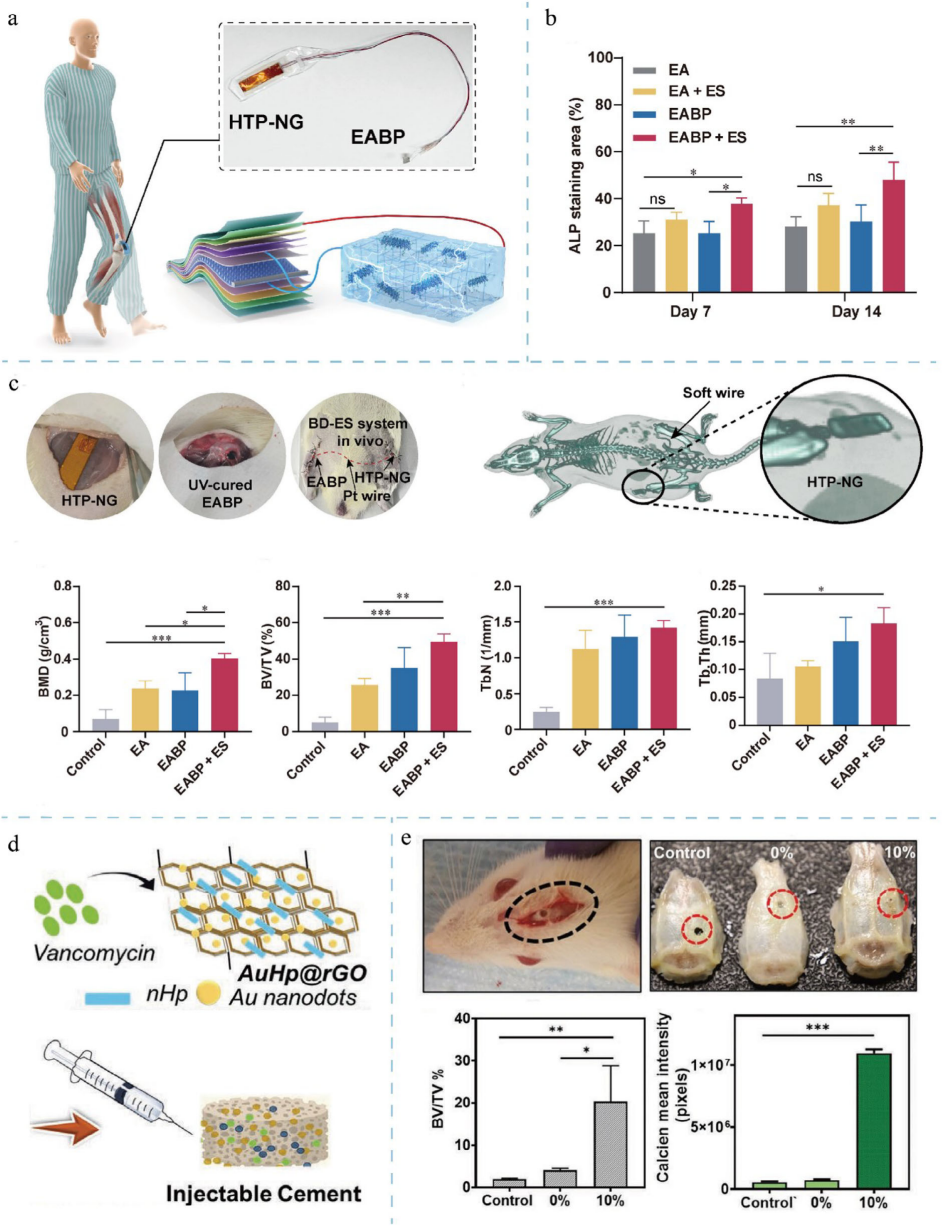
The cases for piezoelectric materials for bone regeneration. a) Structure of the self‐generating bone device. b) In vitro osteogenic capacity of the device. c) In vivo bone repair capacity of the device. Reproduced with permission.^[^ ^165^ ^]^ Copyright 2024, American Association for the Advancement of Science. d) Structure of the injectable piezoelectric bone cement. b) In vivo bone repair capacity of the piezoelectric bone cement. Reproduced with permission.^[^ ^167a^ ^]^ Copyright 2023, Wiley‐VCH.

In terms of material architecture, the field has evolved from rigid scaffolds to injectable systems, which has improved clinical applicability, but there is still a wide variation in material properties and mechanism of focus, complicating standardization. The core idea of preparing piezoelectric hydrogels is to load BT or its modified components into a gelatin matrix.^[^ ^164^ ^]^ Wu et al. loaded PBT and PHA into the chitosan/gelatin (CG) matrix to prepare CG/PHA/PBT piezoelectric hydrogels with storage modulus (SM) and OV positively correlated with the weight ratio of PBT.^[^ ^164a^ ^]^ CG/PHA/5% PBT (SM = 493.1 Pa, OV = 0.6 V) increased M2 macrophages to 21.1% and reduced M1 macrophages to 8.8% (13.5% and 20.0% in CG/PHA), achieving the highest BV/TV of 35.4 ± 4.1% at 8 weeks in vivo.^[^ ^164a^ ^]^ Zhou et al. loaded amino silane coupling agent (KH‐550)‐modified BT NPs (KBTO) into oxidized chondroitin sulfate/gelatin (OCS/Gel) matrices to prepare an injectable OCS/Gel/KBTO ultrasonically responsive piezoelectric hydrogel.^[^ ^164b^ ^]^ Ultrasonic waves with an intensity of 1.5 W cm^−2^ could excite OCS/Gel/0.1% KBTO to produce OVs from −41.16 to 61.82 mV, which were used for subsequent experiments. The results showed that the hydrogel could promote the expression of relevant osteogenic genes (e.g., RUNX2) by activating Ca^2+^ in‐flow and downstream PI3K/AKT and MEK/ERK signaling pathways, which in turn promoted the expression of relevant osteogenic genes.^[^ ^164b^ ^]^ Zheng et al. prepared Gel‐PD‐CMBT hydrogels by loading Ca/Mn co‐doped BT (CMBT) piezoelectric fibers into a matrix consisting of Gel and poly(ethylene dioxythiophene)/polystyrene sulfonate (PD).^[^ ^164c^ ^]^ Both PD and CMBT enhanced the Gel's mechanical properties; the composite gel's maximum strain was 40%, and the gel's compressive modulus (CM) at maximum strain was about 0.6 KPa. The CV curves and electrochemical impedance spectrum (EIS) indicate that the material has good electrical conductivity and piezoelectricity. In vitro, Gel‐PD‐CMBT boosted BMSC osteogenesis with the highest ALP, COL‐I, OPN, and OCN expression, while in vivo, it achieved the highest 8‐week BV/TV (53.38%) and BMD (0.529 g cm^−3^).^[^ ^164c^ ^]^ In addition, Wang et al. established a composite loop system with a flexible generator and a conductive hydrogel (**Figure** **6**a).^[^ ^165^ ^]^ Specifically, they assembled motion‐excited HTP‐NG nanogenerators using PVDF films with Ag electrode layers on both sides as piezoelectric layers, polytetrafluoroethylene (PTFE), and Kapton films as friction electric layers, and added other materials. PDA‐modified black phosphorus nanosheets (BP@PDA) and alginate methacryloyl (AlgMA) were loaded into ECM extracted from porcine subcutaneous tissues, assembled into the conductive ECM‐AlgMA‐BP@PDA (EABP) hydrogel, and connected to the hybrid tribo/piezoelectric nanogenerator (HTP‐NG) via platinum (Pt) soft wires to form a loop. In the femoral condylar defect (FCD) model in rats, the hydrogel was placed at the defect, and power‐generating pads were placed at the joint to achieve motor‐responsive ES at the defect site. The results show that the piezoelectric composite loop increases ALP staining area, with a 1.4‐fold higher BV/TV compared to the EABP group (Figure 6b), elevated calcium ion influx measured by Fluo‐4 AM via flow cytometry, and upregulated PIEZO1/2 expression linked to the PI3K/AKT pathway.^[^ ^165^ ^]^

#### 6.1.3 Piezoelectric Bone Cement

As a commonly used implant in orthopedic surgery, bone cement‐containing antibiotics are effective in preventing infections but may have post‐implantation side effects.^[^ ^166^ ^]^ Therefore, the development of piezoelectric bone cement with good osteogenic capacity and biosafety is of great interest to orthopedics.^[^ ^167^ ^]^ Chopra et al. prepared Au nanodots, nanoHA, and vancomycin‐modified graphene oxide sheets (VAuHp@rGO) and added them to an alginate solution of calcium sulfate and solidified them to obtain novel injectable piezoelectric bone cement (Figure 6c).^[^ ^167a^ ^]^ In vitro and in vivo experiments demonstrate that the 10% cement achieves a flexural strength of 25 ± 1.8 MPa, with 25 ± 9.1% BV/TV (5 ± 1.5% in the control group) (Figure 6d), upregulates VEGF expression by 4.5‐fold in HUVECs, and reduces bacterial survival to 5% with vancomycin release.

Conventional bone cement is mainly composed of polymethylmethacrylate (PMMA), on which the introduction of piezoelectric materials is a common means of preparing piezoelectric bone cement. Wang et al. and Zhang et al. compounded BT NPs and PVDF/polyethyleneimine (PEI) with PMMA to produce PMMA/BT and PMMA/PEI/PVDF piezoelectric bone cement, respectively.^[^ ^167b,c^ ^]^ PMMA/15%BT had an OV of 37.109 V at 152 KPa, 0.85 Hz bionic MS, while PMMA/PEI/PVDF had an OV of 30–130 mV at 1–30 N, 1–5 Hz MS, which seems to be a better piezoelectricity for the former. In both studies, an animal model of FCD was constructed, and cement was added to the defect site and stimulated by animal movement. For the latter, a femoral shaft defect was also established.

The results showed that PMMA‐BaTiO_3_ cement increased new bone volume by 4.3‐fold in the Piezo with exercise group against the Nonpiezo with exercise group after 2 months, upregulating ALP and RUNX‐2 by 1.74 and 2.31 times in 0.65Hz‐20min‐US hBMSCs,^[^ ^167b^ ^]^ while PMMA/PEI/PVDF implants enhanced femoral bending strength (99 MPa, versus 17–48 MPa in other groups) by 12 weeks, with the bone volume hardly mentioned.^[^ ^167c^ ^]^ The limitations of standardized assessments are further demonstrated here. Direct efficacy comparison is limited by differing models and metrics. Of particular importance here is the fact that the baseline biological environment varies in different animal models, which itself results in different rates and times of healing, independent of the material. Furthermore, as Zhang et al. found, the volume of newly added cortical bone in the femoral shaft is significantly larger than in the knee joint due to the higher stress,^[^ ^167c^ ^]^ which means that the stress experienced by the model itself should also be considered in the standardized evaluation process.

The mechanism of osteogenesis remains the classical calcium and mechanosensitive signaling pathways, coinciding with the synergistic mechanism of tissue regeneration stimulated by electromechanical signals as described previously.^[^ ^167b,c^ ^]^

In addition, Lei et al. prepared antimicrobial Se‐modified BT piezoelectric NPs with good ultrasonic response, which showed good anti‐infective and osteogenic effects in an infectious FCD model in rats.^[^ ^163^ ^]^ It seems possible to consider loading this NP into the above PMMA‐based piezoelectric bone cement materials to enhance their antimicrobial and piezoelectric properties.

### 6.2 Cartilage Regeneration

Examples of piezoelectric materials used for cartilage tissue regeneration are shown in Table 4. The role of piezoelectric materials in promoting chondrogenic differentiation of mesenchymal stem cells has been demonstrated in vitro.^[^ ^168^ ^]^ The magnitude of piezoelectricity may be related to the differentiation spectrum of BMSCs: low piezoelectricity promotes chondrogenic differentiation, and high piezoelectricity promotes osteogenic differentiation.^[^ ^168a^ ^]^ Remarkably, this indicates that the strength of piezoelectricity might serve as a critical factor in guiding stem cell differentiation, with lower levels potentially tilting the balance toward cartilage formation rather than bone formation, though this pattern needs broader confirmation across diverse material platforms. Ricotti et al. prepared VitroGel‐RGD hydrogels loaded with BT NPs and graphene oxide (GO) nanosheets with adipose‐derived stem cells (ADSCs) to evaluate the in vitro US‐responsive cartilage‐forming ability of composite piezoelectric hydrogels.^[^ ^168b^ ^]^ After evaluating different frequencies (38 kHz, 1 MHz, and 5 MHz) and different intensities (125, 250, and 500 mW cm^−2^), it was determined that 1 MHz and 250 mW cm^−2^ were the optimal US parameters to promote the expression of the cartilage genes (COL2A1 and ACAN), and to maintain the expression of the proliferative gene (MKI67) at a low level, with a favorable anti‐inflammatory capacity.^[^ ^168b^ ^]^ Although some optimal US settings were pinpointed by Ricotti et al., the influence of different stimulation approaches on long‐term regeneration outcomes is still largely unexplored and merits deeper study.

**Figure 7:**
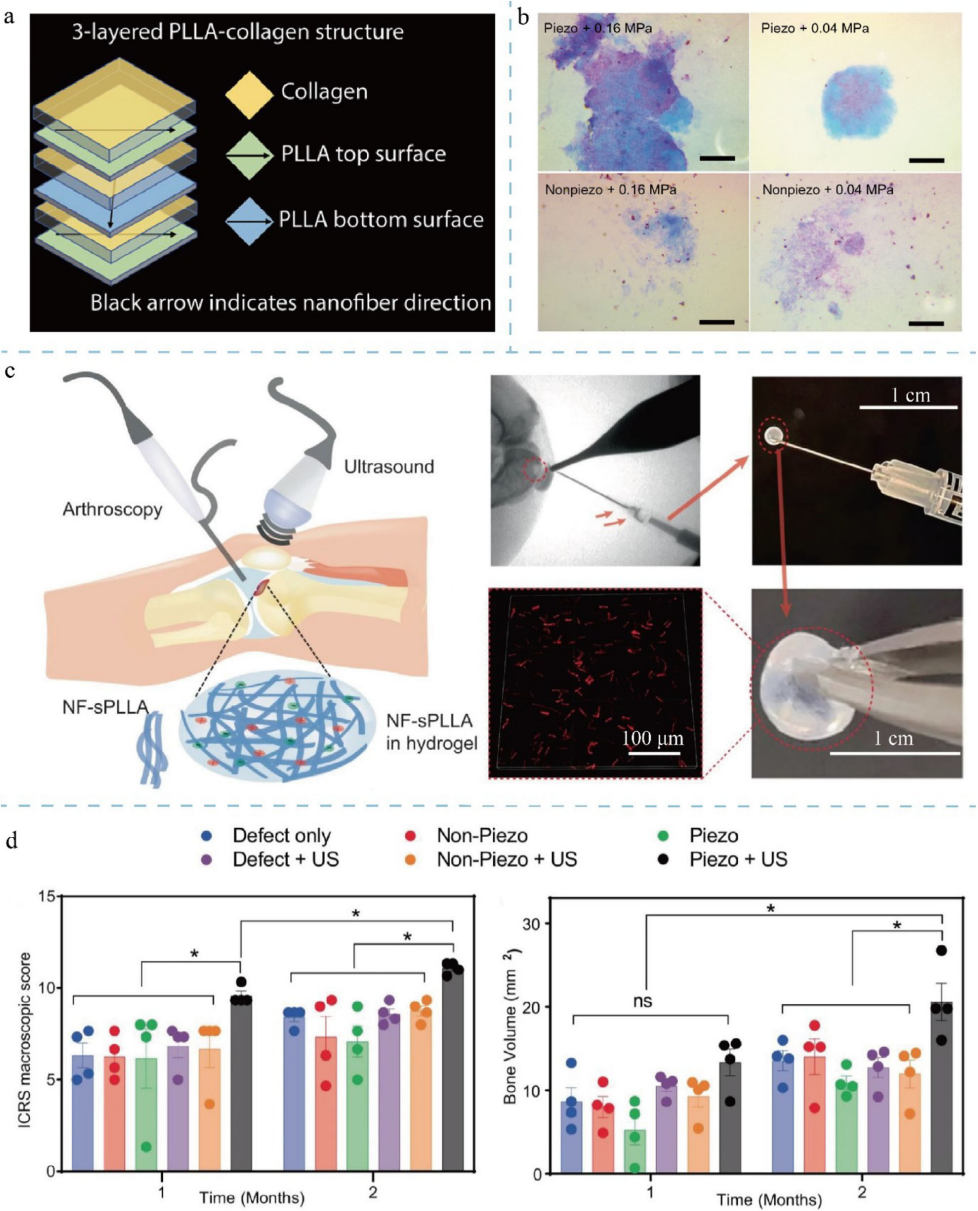
The cases for piezoelectric materials for cartilage regeneration. a) Structure of 3‐layer PLLA‐collagen scaffold. b) The scaffold promotes ADSC deposition of GAGs under different pressures. Scale bars, 200 µm. Reproduced with permission.^[^ ^169b^ ^]^ Copyright 2022, American Association for the Advancement of Science. c) Illustration of the injectable piezoelectric hydrogel. d) The pro‐chondrogenic capacity of the hydrogel in vivo. Reproduced with permission.^[^ ^169c^ ^]^ Copyright 2023, Nature Pub. Group.

Piezoelectric materials have also been used successfully in animal models of cartilage damage (mainly rabbits at present).^[^ ^169^ ^]^ A notable step forward in this area is the shift from non‐degradable options like PVDF to biodegradable materials such as PLLA, enhancing their suitability for in vivo use by tackling biocompatibility challenges. Wu et al. prepared a PVA/PVDF hydrogel loaded with Ag NWs with antimicrobial capacity for exercise‐inspired repair of cartilage defects in rabbits.^[^ ^169a^ ^]^ However, PVDF has poor in vivo degradability. Therefore, research on degradable PLLA materials has been emphasized.^[^ ^169b,c^ ^]^ Liu et al. prepared a three‐layer PLLA scaffold with the aligned fibers of the upper and lower layers oriented in the same direction and the middle layer oriented perpendicular to it, and the three layers were cross‐linked with collagen hydrogel to improve the hydrophilicity of the scaffold (**Figure** **7**a). This kind of scaffold has good mechanical properties (642±84 MPa) and produces a voltage output of 3.6 V at an impact force of 33N.^[^ ^169b^ ^]^ In vitro experiments showed that the negative surface had a better ability to promote chondrogenic differentiation of ADSCs compared with the positive pole due to the up‐regulated expression of chondrogenic genes (COL2A1, ACAN, and SOX9s) and glycosaminoglycans (GAGs) (Figure 7b) by recruiting ECM proteins and activating the calcium pathway and TGF‐β pathway. After the stent was implanted into the cartilage defect site of the rabbit, it was left to rest for a month and then supplemented with 20 minutes of daily treadmill exercise for optimal repair. The findings indicated that two months of exercise resulted in complete healing of the cartilage.^[^ ^169b^ ^]^ Vinikoor et al. prepared an injectable PLLA/collagen hydrogel with the ability to fit various shaped defects (Figure 7c), although its output voltage (33.7 mV) was not as ideal as in the previous study.^[^ ^169c^ ^]^ US treatment (20 min, 0.5 W cm^−2^, 40 kHz) was used to excite the hydrogels in vitro and in vivo. The results showed that the US‐excited material could enhance the expression of SOX9, ACAN, and COL2A1 genes by recruiting stem cells as well as promoting TGF‐β1 secretion. After two months of US treatment, the hydrogel‐injected rabbit cartilage defects showed white cartilage similar to natural tissue, with significantly higher ICRS scores (Figure 7d).^[^ ^169c^ ^]^ These findings showcase diverse activation strategies for piezoelectric materials, spanning mechanical stimulation via treadmill exercise (Liu et al.) to ultrasound triggering (Vinikoor et al.), underscoring the importance of tailoring these approaches to specific tissue repair objectives. Although promising in rabbit models, validating these results in larger animal models is critical to evaluate the mechanical properties and durability of regenerated cartilage, especially for human applications. The injectable piezoelectric hydrogels developed by Vinikoor et al. represent a key translational advance, offering a minimally invasive treatment option, yet their long‐term performance in dynamic joint environments remains unclear. Future research should focus on larger‐scale animal studies to explore the interplay between piezoelectric stimulation and biochemical pathways (e.g., TGF‐β signaling) and rigorously assess the durability of injectable systems. Overcoming these hurdles will pave the way for clinically viable therapies for cartilage defects.

### 6.3 Peripheral Nerve Regeneration

Piezoelectric materials have a long history of use in nerve regeneration. Since the early 1890s, piezoelectric PVDF conduits have been used for nerve regeneration.^[^ ^170^ ^]^ These piezoelectric materials, predominantly PVDF‐based, have transformed peripheral nerve regeneration by converting mechanical stress into electrical signals that mimic the bioelectrical cues vital for nerve tissue repair. Recent applications of piezoelectric materials in the field of peripheral nerve regeneration and spinal cord regeneration are summarized in Table 5. In this section, we focus on the important results of piezoelectric materials in peripheral nerve regeneration.

PVDF is widely used in peripheral nerve regeneration due to its good piezoelectric properties and flexibility, and most of the new piezoelectric nerve conduits are designed on the basis of PVDF.^[^ ^171^ ^]^ Zhang et al. added different concentrations of MXene (Ti3C2Tx) powder into a mixture of silk fibroin (SF) and poly(vinylidene fluoride‐co‐hexafluoropropylene) (PVDF‐HFP), and electrostatic spinning was used to obtain SF/PVDF‐HFP/MXene composite membrane, which was further processed into a nerve conduit.^[^ ^171a^ ^]^ SF and MXene were used to improve the biocompatibility and antimicrobial properties of the conduits, respectively. The material with the addition of 3% w/v MXene had the best tensile properties (the maximal stretch of 140%) and OV_max_ (100 mV) with more than 90% bacterial inhibition and was therefore used for in vivo and in vitro experiments. The results showed that treadmill exercise could stimulate the conduit to generate piezoelectric signals to promote sciatic nerve regeneration and inhibit gastrocnemius muscle atrophy.^[^ ^171a^ ^]^ This work highlights the versatility of PVDF, with MXene enhancement amplifying piezoelectric output to support Schwann cell proliferation and axonal growth, key drivers of nerve repair.

**Figure 8:**
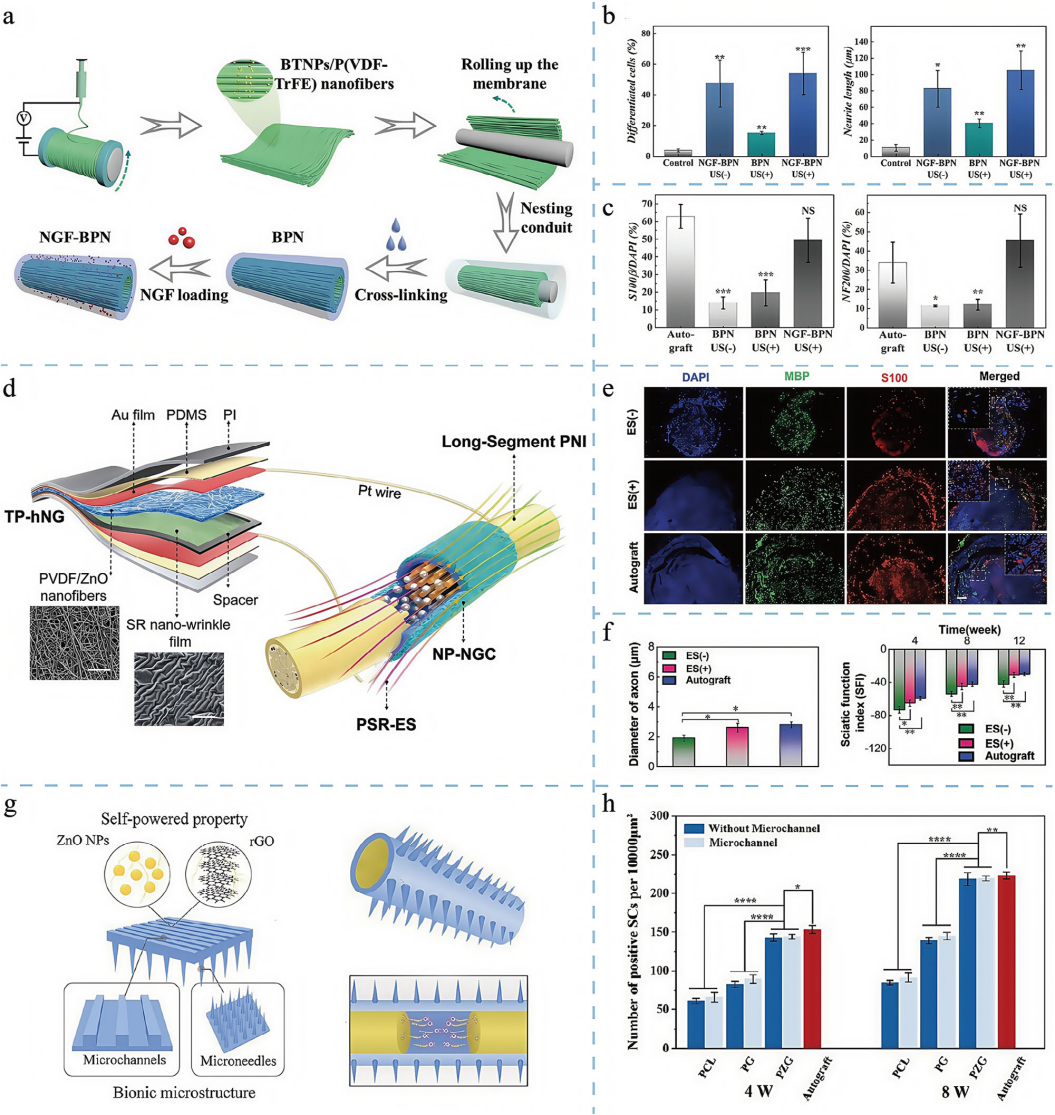
The cases for piezoelectric materials for peripheral nerve regeneration. a) Production process of the piezoelectric hydrogel conduit. b) Pro‐neural differentiation capacity in vitro. c) Promotion of neural markers S100β and NF200 in vivo. Reproduced with permission.^[^ ^171c^ ^]^ Copyright 2024, Wiley‐VCH. d) Structure of the self‐generating electrical nerve stimulation system. e) Promotion of neural markers MBP and S100 in vivo. f) Promotion of nerve regeneration motor function recovery in vivo. Reproduced with permission.^[^ ^173a^ ^]^ Copyright 2021, Wiley‐VCH. g) Structure of the microneedle nerve conduit. h) Promotion of neurogenesis in vivo. PG, PCL/rGO. PZG, PCL/Zno NPs/rGO. Reproduced with permission.^[^ ^173c^ ^]^ Copyright 2024, American Chemical Society.

Since rats with sciatic nerve injury objectively lack active locomotion ability, activation of the piezoelectric conduit in a passive manner may be a better choice in comparison.^[^ ^171b,c^ ^]^ Tai et al. used a 4‐bar intensity in vitro shockwave to excite the P(VDF‐TrFE) conduit in rats, which produced an OV of about 200 mV and promoted axonal and myelin regeneration as well as motor function recovery in rats.^[^ ^171b^ ^]^ By leveraging shockwave stimulation, Tai et al. demonstrated how external methods can precisely harness PVDF's piezoelectric properties to enhance therapeutic outcomes in nerve regeneration. Recently, Xu et al. provided an excellent work that skillfully combines the mechanical and thermal effects of US, the drug‐carrying capacity of hydrogels, and the piezoelectric properties of nerve conduits.^[^ ^171c^ ^]^ They introduced BT NPs and fabricated BTNPs/P(VDF‐TrFE) conduits with aligned nanofibers by electrostatic spinning method and encapsulated them with thermosensitive poly(N‐isopropyl acrylamide) hybrid hydrogel loaded with nerve growth factor (NGF) in the outer layer, producing a US‐responsive NGF‐releasing piezoelectric hydrogel nerve conduit (**Figure** **8**a).^[^ ^171c^ ^]^ Considering that 7 wt% BTNPs/P(VDF‐TrFE) produces the maximum OV (10.51 V) under a cyclic pressure stimulation of 30 N and exhibits adequate tensile properties, it was selected for subsequent experiments. The underwater OV by 0.75 W cm^−2^ US excitation was 1.69 V. The conduit promoted the differentiation and neurite extension of PC12 cells under 1 MHz, 0.75 W cm^−2^ US treatment in vitro (Figure 8b). US stimulation of the same intensity was further used to activate hydrogel conduits grafted to the rat sciatic nerve for 5 minutes per day during the first week, and 30 minutes per day during the second week, and efficacy was assessed eight weeks after the procedure. Evaluation of sciatic nerve regeneration and motor function recovery showed that US promotes ES and NGF release from the material, and aligned fibers guide nerve growth, with an increase in the percentage of immunofluorescence‐positive regions for the neuromarkers S‐100β and NF200 (Figure 8c).^[^ ^171c^ ^]^ Xu et al.’s design exemplifies a paradigm shift, integrating ultrasound‐activated piezoelectricity with drug delivery to synergistically provide electrical and biochemical cues, significantly advancing nerve growth guidance. Xiong et al. also developed an aligned piezoelectric nerve conduit. The difference is that they introduced polypyrrole (PPy) component to prepare PPy/PDA/PLLA conduit, which makes it have the function of scavenging ROS, but its biodegradability is not ideal.^[^ ^172^ ^]^ This innovation underscores a critical trade‐off: while PPy enhances functionality by mitigating oxidative stress, its poor biodegradability poses challenges for clinical applicability.

In recent years, a number of other novel designs have emerged.^[^ ^173^ ^]^ Similar to the above‐mentioned EABP/HTP‐NG composite device repairing FCD with the help of piezoelectric signals generated by the movement of the knee joint,^[^ ^165^ ^]^ Jin et al. designed a piezoelectric loop device that generates ES with the help of natural movements of the chest wall (e.g., breathing, heartbeat) to repair nerves.^[^ ^173a^ ^]^ They loaded ZnO onto a PVDF nanofiber mat as the negative layer and silicone rubber with a rough topological surface as the positive layer to form a tribo/piezoelectric hybrid nanogenerator (TP‐hNG) capable of providing piezoelectricity and friction electricity (Figure 8d). When the working area is 16 cm^2^, the OVmax can reach an amazing 500 V. The inner layer of chitosan and PD and the outer layer of PLLA were assembled into a nanoporous nerve guide conduit (NP‐NGC), which was implanted in the sciatic nerve defects of the rats and was connected to a generator implanted in the subcutaneous adipose tissue of the chest via platinum wire. Experimental results at 12 weeks postoperatively showed that this self‐generating loop system was comparable to autografts for nerve regeneration and motor function recovery (Figure 8e,f).^[^ ^173a^ ^]^ Jin et al.’s reliance on natural motion highlights its potential as a non‐invasive stimulation source, yet it sparks debate: while practical, it lacks the precision of external methods like ultrasound or shockwaves, potentially compromising regeneration consistency. Tan et al. established a skin full‐excision model of the rat dorsum to investigate the role of piezoelectric patches on cutaneous nerve regeneration and sensory recovery.^[^ ^173b^ ^]^ The piezoelectric smart patch, consisting of polarized PVDF film and GelMA gel (gelatin/methacrylic anhydride) loaded with rGO, CXCL12 chemokine, and G‐Exos (ginseng‐derived exosome‐like vesicles), was placed in the wound home position. Wound tissue CD90 expression was used to assess the recruitment of BMSCs. Scratching movements of rats to itchy skin sites (itchy skin model) and the expression of nerve‐related proteins were used to assess the regeneration of cutaneous nerves. The results showed that the smart patch was able to achieve in situ regeneration of skin nerves and sensation within 23 days, possibly by releasing CXCL12 to promote the recruitment of BMSCs. And ES and G‐Exos promoted neural differentiation.^[^ ^173b^ ^]^ Recently, Hu et al. provided an interesting study in which they prepared piezoelectric PCL/ZnO NPs/rGO (PZG) conduits indicating a micro‐needle (MN) structure on the outside and a micro‐channel structure on the inside by simulating the morphology of sea cucumber (Figure 8g).^[^ ^173c^ ^]^ The microneedle structure pierces muscle tissue near the nerve to provide ES and prevent atrophy, and the microchannel topology guides the directional growth of the nerve. In vitro and in vivo experiments showed that the PZG conduit promoted Schwann cell viability (Figure 8h), nerve regeneration, and vascularization and inhibited gastrocnemius muscle atrophy. Immunofluorescence staining showed that piezoelectric signals had a better effect in promoting nerve regeneration compared with topography.^[^ ^173c^ ^]^ Hu et al.’s microneedle/microchannel architecture illustrates how piezoelectric materials can integrate with surrounding tissues, like muscle, to address multifaceted aspects of nerve injury recovery.

Collectively, these findings suggest piezoelectric conduits accelerate peripheral nerve repair by modulating cell signaling, potentially via voltage‐gated ion channels in Schwann cells. Their flexibility and ability to replicate bioelectrical cues are key strengths, yet inconsistent stimulation methods and biodegradability challenges persist. The optimal activation strategy—natural motion versus precise external techniques—remains unresolved. Future efforts should prioritize biodegradable piezoelectric composites and implantable microdevices delivering tailored, sustained electrical and topological cues for peripheral nerve needs. While animal models are promising, long‐term safety and standardized protocols are essential to translate these advances into clinical practice, aligning them with the complexities of human nerve regeneration.

### 6.4 Central Nervous System Repair

Building on advances in peripheral nerve repair, the application of piezoelectric materials in central nervous system (CNS) repair—including both spinal cord and retinal repair—is logical and promising. The electromechanical conversion capabilities, as well as the structural support of piezoelectric materials, can be used to stimulate and direct neuronal growth in the spinal cord, close to their application in peripheral nerves. Meanwhile, P(VDF‐TrFE) materials have garnered increasing attention in retinal repair due to their remarkable flexibility and tunable capacitance.^[^ ^174^ ^]^ These materials can convert the mechanical strain generated by light‐induced isomerization into changes in capacitance and voltage, thereby modulating ion channels and mimicking neural impulses.^[^ ^175^ ^]^ This ability is a crucial component in the photonic signal transmission of artificial retinas. Recent studies highlight the ability of piezoelectric materials to restore or enhance neural function in models of both spinal cord injury and retinal degeneration (Table 5).^[^ ^176^ ^]^ This growing field holds great promise for the development of effective treatments for CNS injuries, potentially transforming the landscape of neuroregenerative medicine.

**Figure 9:**
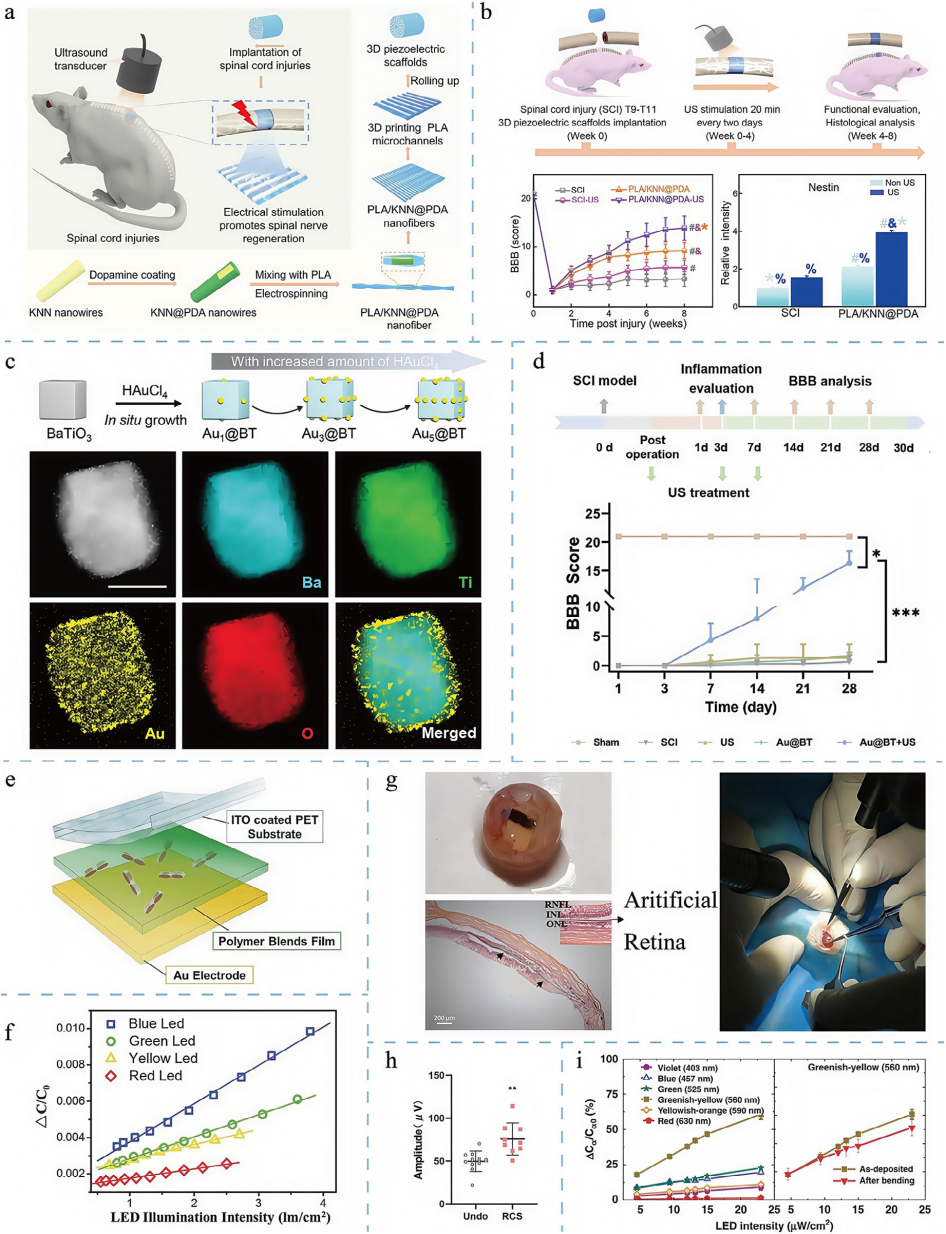
The cases for piezoelectric materials for central nervous system repair. a) Structure of the 3D piezoelectric PLA/KNN@PDA scaffold. b) Promotion of spinal cord injury recovery in vivo. Reproduced with permission.^[^ ^176a^ ^]^ Copyright 2022, American Chemical Society. c) Structure of the Au@BT nanoparticles. Scale bar, 100 nm. d) Motor function recovery properties in vivo. Reproduced with permission.^[^ ^176b^ ^]^ Copyright 2024, Wiley‐VCH. e) Structure of the photo‐sensitive artificial retina. f) Capacitance response curve under different lighting conditions. Reproduced with permission.^[^ ^174c^ ^]^ Copyright 2016, Wiley‐VCH. g) Artificial retina implantation surgery. h) Enhanced flash visual evoked potential. Reproduced with permission.^[^ ^174a^ ^]^ Copyright 2020, American Chemical Society. i) Capacitance response curves under different lighting and bending conditions. Reproduced with permission.^[^ ^174b^ ^]^ Copyright 2022, Springer Nature.

For spinal cord repair, Chen et al. provided a good example.^[^ ^176a^ ^]^ They loaded PDA‐modified KNN nanowires into PLA substrates and fabricated PLA/KNN@PDA piezoelectric films with aligned PLA microbands by electrostatic spinning, which were rolled up to obtain piezoelectric 3D scaffolds with multiple channels (**Figure** **9**a). The scaffolds were encapsulated into generators with Au electrodes and PDMS to evaluate piezoelectric performance. At a pressure of 0.5 N, 10 Hz, OV_max_ increased from 0.52 V to 17.9 V, and OC_max_ increased from 0.12 to 2.6 µA. Under ultrasonic excitation at a frequency of 1 MHz, a pulse width of 5 µs, a pulse interval of 10 ms, and an acoustic pressure of 150 KPa, the OV is 12.09 V, and the OC is 20.8 µA when the generator is in deionized water. And when the generator is in the body, the OV is 11.56 V. The above data demonstrates good piezoelectric properties. The scaffolds boosted neural differentiation in vitro, with Nestin, Tuj1, and MAP2 mRNA levels rising (p < 0.05) in the PLA/KNN@PDA‐US group against the PLA group after 7 days. The rat spinal cord was created with a 2‐mm transection at T9‐T11, implanted with a 3D scaffold, and treated with US at 100 KPa for 20 min every 2 days for 4 weeks. The efficacy of the treatment was observed after 2 months. It was found that US treatment raised BBB scores (*p* < 0.05) by week 8 (Figure 9b), enhanced myelin (LFB up, *p* < 0.05), neural differentiation (Nestin up, *p* < 0.05 (Figure 9b)), and angiogenesis (CD31/VEGF up, *p* < 0.05) in rats, indicating the important role of piezoelectric signals in the repair of the spinal cord.^[^ ^176a^ ^]^

Recently, You et al. developed a piezoelectric nanoparticle, Au@BT, by loading Au NPs onto the surface of BT NPs, which demonstrated ultrasound‐responsive H_2_ release capabilities.^[^ ^176b^ ^]^ The construction of the Schottky heterojunction endowed the NPs with excellent piezo‐catalytic efficiency (Figure 9c). The generated H_2_ can react with ROS to form H_2_O, exhibiting significant antioxidative stress and cytoprotective properties in vitro. The underlying mechanism is likely through the activation of signaling pathways such as PI3K/AKT, providing anti‐apoptotic effects. Medical chitosan hydrogels loaded with Au@BT NPs were injected into the lesion site of rat models with spinal cord contusion. Ultrasound treatment (1.75 W cm^−2^, 5 min, twice) was administered on postoperative days 3 and 7. The results indicated that the Au@BT NPs, when activated by ultrasound, significantly reduced inflammation and promoted the recovery of hind limb motor function in rats with spinal cord injury (Figure 9d).

These contrasting approaches illuminate a dynamic interplay between material innovation and the specific challenges of spinal cord repair. While Chen et al.’s 3D scaffolds leverage aligned microchannels and electrical stimulation to provide a structural framework for neural growth and vascularization, You et al.’s nanoparticles shift the focus to biochemical modulation, releasing hydrogen under ultrasound to combat oxidative stress. This strategic divergence—structural versus molecular facilitation—suggests a hybrid strategy combining the physical guidance of scaffolds with the molecular benefits of nanoparticles could yield a more holistic repair approach. Yet, the field remains divided on optimal material properties, debating rigid scaffolds versus injectable nanoparticles, which complicates scalability and integration into clinical settings.

For retinal repair, Shen's team has made significant contributions. In 2016, they reported a P(VDF‐TrFE)‐based photodetector,^[^ ^174c^ ^]^ consisting of a flexible, high‐transmittance electrode layer of indium tin oxide (ITO)‐coated polyethylene terephthalate (PET), a photoelectric signal transduction layer incorporating light‐responsive azo‐benzene polymer P(8‐AZO‐10) with P(VDF‐TrFE), and a layer of Au electrode (Figure 9e). Specifically, under illumination at various wavelengths, the trans/cis isomer ratios of P(8‐AZO‐10) are altered (Figure 9f), mimicking the light sensitivity of retinal pigments in the human eye (e.g., rhodopsin). This rapid mechanical motion, generated during the isomerization, is converted into an electrical signal by P(VDF‐TrFE), thereby facilitating calcium ion signaling among neuronal cells. Theoretically, this device offers sufficient animation‐capturing ability (able to distinguish stimuli appearing every 100–500 ms) and resolution (25 000 PPI) to satisfy the human eye's requirements. Although achieving an artificial retina with image resolution comparable to the human retina remains distant—challenges include sensitivity differences across color spectra (human: green; artificial: blue)—this work represents a highly promising strategy for retinal biomimetics. Additionally, the team attempted to construct a photoelectric transduction layer by combining folate‐azide‐functionalized poly(ethyl 5‐(thiophen‐3‐yl)pentanoate‐2,5‐diyl) (P3EPT‐FA) with P(VDF‐TrFE) and [6,6]‐phenyl‐C61‐butyric acid methyl ester (PCBM), sandwiched between ITO and Au electrodes.^[^ ^174a^ ^]^ This composite device was then implanted into the subretinal space of dystrophic Royal College of Surgeons (RCS) rats, a model for retinal degeneration (Figure 9g), where the implanted group demonstrated an enhanced flash visual evoked potential (Figure 9h). More recently, Vijjapu et al. assembled an artificial retina by sandwiching a perovskite ferroelectric nanocomposite (PFNC) between Al and Au electrodes.^[^ ^174b^ ^]^ The PFNC consists of poly(vinylidene fluoride‐trifluoroethylene‐chlorofluoroethylene) (PVDF‐TrFE‐CFE) and methylammonium lead bromide perovskite (MAPbBr3). This device demonstrates a strong linear response to various wavelengths of light, with the highest sensitivity observed in the yellow‐green spectrum, similar to the human retina, and maintains robust performance under bending conditions (Figure 9i). Notably, this study points out that the materials used in this artificial retina are non‐degradable, suggesting that replacement‐oriented materials, similar to orthopedic implants in joint surgeries, can indeed be non‐degradable. While their durability suits prosthesis‐like replacement, it clashes with repair objectives that demand material absorption and seamless tissue integration. This inherent conflict between immediate functionality and long‐term biocompatibility may confine these technologies to severe injury cases, where replacement overshadows regeneration. This perspective may evolve as the field progresses, shifting from a “replacement” approach aimed at severe damage to a more universal “repair” strategy. Compounding this challenge is the limited scope of robust in vivo studies, with successes largely confined to preliminary models, exposing a critical gap between laboratory promise and clinical reality.

Moreover, there has been a remarkable success in 3D‐printing retinal cells.^[^ ^177^ ^]^ This naturally sparks the imagination: combining piezoelectric materials like P(VDF‐TrFE) with photosensitive materials and 3D cell printing technology could be a groundbreaking path toward retina repair, potentially restoring light to those who have lost their vision. However, like spinal cord injury repair, the lack of animal studies highlights the challenges in translating these technologies into in vivo applications. These challenges underscore the exploratory and promising future of CNS repair. Yet, both of these visionary paths reaffirm the translational potential of piezoelectric materials in CNS repair.

### 6.5 Wound Healing

Piezoelectric materials play an important role in wound healing, and representative studies in recent years have been summarized in Table 6. Recent research underscores their dual capacity to combat infection and accelerate tissue regeneration within skin‐specific environments. Specifically, the materials can achieve antimicrobial effects through piezoelectric catalytic effects,^[^ ^178^ ^]^ as well as ES to promote wound tissue regeneration in different contexts, such as diabetes and burns,^[^ ^179^ ^]^ and the prevention of scar formation.^[^ ^180^ ^]^ A common thread across these investigations is the application of mechanical stimuli—either via US or natural tissue movements—to trigger the piezoelectric effect, producing electrical signals or ROS that underpin therapeutic outcomes.

Zhu et al. loaded zeolitic imidazolate framework‐8 (ZIF‐8) core shells with ciprofloxacin (CIP) on the surface of BT NPs.^[^ ^178a^ ^]^ They created a Staphylococcus aureus‐infected full‐length wound on the back of the mice, added pellets, closed the wound with a medical isolation membrane, and supplemented it with US treatment (1.5 W cm^−2^, 1 MHz, 50% duty cycle, 1 min) 24 hours after the procedure. At the initial stage of treatment, the composite NPs have a stronger ROS generation efficiency than BT NPs due to the synergistic effect of BT and ZIF‐8. It works together with the released CIP to achieve the desired antimicrobial effect. With the degradation of ZIF‐8, ROS levels decreased, and zinc ions were released, promoting wound healing.^[^ ^178a^ ^]^ Zhu et al.’s use of BT exemplifies a strategic approach to tailoring the piezoelectric response to the skin's mechanical and biological milieu, with US stimulation amplifying antimicrobial efficacy.

Similarly, Chen et al. loaded BT particles with surface‐deposited silver nanodots into a composite gel of SF and poly (ethylene glycol) diacrylate (PEGDA).^[^ ^178b^ ^]^ SF was modified to SF‐MA with glycidyl methacrylate (GMA) to facilitate 3D printing. A 5‐min US treatment (1.5 W cm^−2^, 1 MHz) was performed 24 hours after mouse surgery. In vitro and in vivo experiments showed excellent killing of *E. coli* and *Staphylococcus aureus*, respectively, with a wound healing rate of 99.9% on day 12.^[^ ^178b^ ^]^

However, the reliance on US stimulation raises unresolved questions about its safety, particularly concerning tissue heating and long‐term effects, which remain insufficiently explored and could complicate clinical adoption.

Wang et al. established a rat model of type Ι diabetes mellitus (T1DM) and fabricated a full dorsal wound to investigate the ability of PVA/PVDF (7:3 ratio) piezoelectric composite hydrogel to repair diabetic wounds.^[^ ^179a^ ^]^ Piezoelectric signals were excited by natural traction on rat back tissues. The results showed that PVA/PVDF piezoelectric hydrogel accelerates diabetic wound repair by reducing the epithelial gap to complete closure by day 14 (still gaps in other groups).^[^ ^179a^ ^]^ Wang et al.’s work highlights the synergy between PVDF's piezoelectric properties and the diabetic wound environment, where natural traction enhances cellular regeneration, reflecting a deliberate customization of material choice to wound‐specific challenges.

Likewise, Chen et al. prepared poly(N, N‐dimethylacrylamide) (PDMAA)/PVDF hydrogel patches for repairing first‐ and second‐degree scalded skin in mice, where daily 10‐minute massages were used to elicit piezoelectric signals. The results showed that the PVDF/PDMAA30 patch, when massaged daily for 10 minutes, enhances scalded skin regeneration by promoting collagen deposition and increasing hair follicles, glands, and blood vessels. On day 18, treated grade I scald wounds exhibited a compact dermis with abundant hair follicles and blood vessels, compared to sparse regeneration in untreated controls.^[^ ^179b^ ^]^ Liang et al. prepared PVDF/sodium alginate (SA) composite hydrogel scaffolds loaded with ZnO NPs with excellent tensile strength (TS, 1415 KPa) at the optimal concentration of ZnO (0.5%).^[^ ^180^ ^]^ The piezoelectric properties were described as bidirectional piezoelectricity, whereby piezoelectricity was generated in two distinct phases: initially, during the early stages of the rat skin defect, when exudates were absorbed by the scaffold, causing it to expand vertically, and subsequently, during the later stages, the skin rubbed horizontally against the scaffold. It has been postulated that the reduction in α‐SMA protein and the concomitant increase in growth factor expression during the late healing phase serve as mechanisms to prevent the formation of scar tissue.^[^ ^180^ ^]^ The strategic selection of PVDF in this design adapts to the dynamic mechanical environment of the healing skin, illustrating how material choice can influence therapeutic outcomes across different healing stages.

In addition to their use in the skin, piezoelectric materials have been demonstrated to facilitate wound healing in the oral mucosa.^[^ ^181^ ^]^ Chernova compared the ability of PTFE and a copolymer of vinylidene fluoride and tetrafluoroethylene (P(VDF‐TeFE)) membranes to repair the oral mucosa of rats. The results demonstrated that the P(VDF‐TeFE) group exhibited faster mucosal healing and less scar formation than the PTFE group. Additionally, the TS of the P(VDF‐TeFE) film was three times higher than that of the PTFE film.^[^ ^181^ ^]^

**Figure 10:**
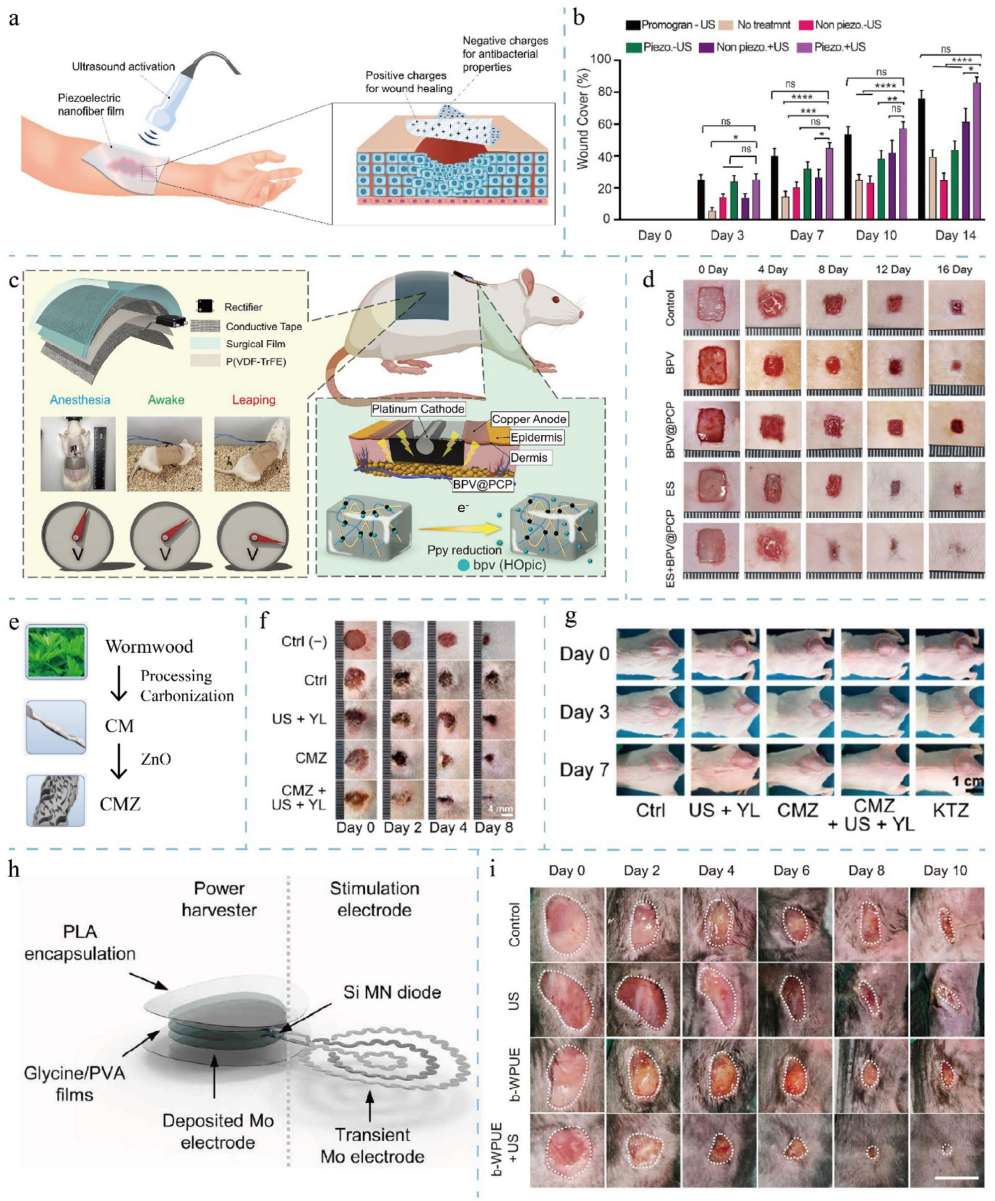
The cases for piezoelectric materials for wound healing. a) Illustration of the PLLA skin‐wound scaffold. b) Promotion of wound healing in vivo. Reproduced with permission.^[^ ^182b^ ^]^ Copyright 2023, Elsevier. c) Structure of the piezoelectric skin patch. d) Promotion of wound healing in vivo. Reproduced with permission.^[^ ^182c^ ^]^ Copyright 2023, American Chemical Society. e) Structure of the moxa‐modified zinc oxide nanosheets. f,g) Anti‐superficial and deep fungal resistance of nanosheets in vivo. CM, carbonized moxa. CMZ, carbonized moxa@ZnO. KTZ, ketoconazole. Reproduced with permission.^[^ ^182d^ ^]^ Copyright 2024, American Chemical Society. h) Structure of the γ‐glycine/PVA composite device. i) Promotion of wound healing in vivo. Scale bar, 6 mm. Reproduced with permission.^[^ ^182e^ ^]^ Copyright 2024, American Association for the Advancement of Science.

Enriching the structure and function of materials and improving their degradability, piezoelectric simplicity, and piezoelectric stability have been the focus of research in recent years.^[^ ^182^ ^]^ Huang et al. fabricated a piezoelectric patch with a Janus structure comprising an upper layer of PEGDA hydrogel loaded with Au‐modified BT NPs for piezoelectric catalysis and a lower layer of GelMA loaded with VEGF for promoting tissue regeneration.^[^ ^182a^ ^]^ The patches were utilized to treat wounds infected with Staphylococcus aureus in rats, with the addition of 3 min of 1.5 W cm^−2^ US treatment per day for the previous 3 days. The results demonstrated that this patch exhibits a favorable US response for sterilization and tissue regeneration.^[^ ^182a^ ^]^ Yet, the incomplete degradability of BT poses a potential hurdle for clinical translation, as non‐biodegradable residues could interfere with the skin's remodeling process. Das et al. prepared biodegradable piezoelectric PLLA scaffolds (OV = 300 mV) (**Figure** **10**a) by controlling the electrostatic spinning parameters (3000 rpm) and optimizing the process (annealing, cutting at 45°) and subsequently implanted them in the back defect site of rats.^[^ ^182b^ ^]^ Notably, possibly based on trust in the degradability of PLLA, they buried the edges of the scaffolds under the skin rather than fixing them to the surface of the skin. In vitro, results showed that the scaffold had a good piezoelectric catalytic function and showed a significant killing effect against Staphylococcus aureus and Pseudomonas aeruginosa. A 20‐min treatment with 40 kHz US at a power density of 0.12 W cm^−2^ was administered to mice in vivo for a period of 2 weeks, with five treatments per week. Under the US, the negatively charged side of the PLLA scaffold acted as an antimicrobial agent, and the positively charged side acted as a skin regeneration promoter, and complete wound closure was observed in 14 days (Figure 10b).^[^ ^182b^ ^]^

The phosphatase and tensin homolog (PTEN) is a molecular brake that inhibits cell proliferation and differentiation. Therefore, inhibition of PTEN may be a potential way to promote tissue regeneration. Fu et al. loaded the PTEN inhibitor bpV(Hopic) into a PAM/carboxymethyl chitosan (CMCS)/PPy composite hydrogel and placed it in the dorsal wound of rats (Figure 10c).^[^ ^182c^ ^]^ Wearable piezoelectric power‐generating patches were made by adding P(VDF‐TrFE) pads in the middle of the conductive tapes and overlaid with medical PU tape. This kind of patch had voltage output capabilities of 0.42 V, 1.32 V, and 2.63 V during anesthesia, wakefulness, and jumping in rats, respectively, and was connected to the hydrogel through a rectifier and electrodes. Fifteen minutes of treadmill exercise per day and shaving treatments every 4 days were applied to energize the piezoelectric patches and prevent device shedding, respectively. Drug release assays showed that bpV concentrations could be controlled within the therapeutic window (50–300 µg L^−1^). Experiments in rats showed that the composite device increased the rate of wound healing by 21.33% (Figure 10d).^[^ ^182c^ ^]^ Fu et al.’s integration of piezoelectric systems with a pharmacological inhibitor suggests a promising synergy with emerging therapies, such as gene or stem cell approaches, which could amplify therapeutic efficacy in future applications.

Based on the possible anti‐inflammatory function of the traditional Chinese medicine moxa, Weng et al. prepared carbonized moxa‐modified ZnO nanosheets for the treatment of subcutaneous and deep fungal infections (Figure 10e).^[^ ^182d^ ^]^ Yellow light (5.5 W cm^−2^) and US (1.5 W cm^−2^, 1 MHz, 50% duty cycle) were used to achieve synergistic treatment of photocatalysis and piezoelectric catalysis to enhance the bactericidal ability of the nanosheets in a two‐day cycle of eight cycles of 30 seconds each. The results showed that the nanosheets had significant efficacy in repairing fungal‐infected wounds (Figure 10f,g).^[^ ^182d^ ^]^ Recently, Xue et al. prepared degradable piezoelectric devices containing γ‐glycine/PVA (2:1) films (Figure 10h) with good piezoelectric properties due to the oriented growth of γ‐glycine (d_33_ = 10.4 pC N^−1^, g_33_ = 324 × 10^−3^ Vm N^−1^).^[^ ^182e^ ^]^ US (1 MHz, 0.1 W cm^−2^, 60% duty cycle) for 5 min for seven consecutive days was used to excite the films to assess the ability of the generated piezoelectric signals to repair wounds on the back of rats. The results demonstrated that piezoelectric devices exhibited notable tissue repair efficacy in response to the US, with a 40% reduction in wound repair time (Figure 10i).^[^ ^182e^ ^]^

While these findings affirm the potential of piezoelectric interventions, they also underscore the need for standardized material selection and stimulation protocols to optimize outcomes across diverse wound types. A deeper mechanistic understanding of how piezoelectric signals affect skin‐specific cellular processes, such as keratinocyte migration and fibroblast activity, could open up new design principles for next‐generation materials, ultimately advancing the clinical translation of piezoelectric technology in regenerative dermatology.

### 6.6 Dental Tissue Regeneration

Recent piezoelectric applications in dental tissue, tendon, myocardial, and corneal regeneration are summarized in Table 7. Piezoelectric materials are currently being developed in dentistry for the treatment of inflammatory periodontal defects,^[^ ^183^ ^]^ promotion of gingival healing,^[^ ^184^ ^]^ and regeneration of dentin,^[^ ^185^ ^]^ based on their anti‐inflammatory and antimicrobial abilities as well as tissue regeneration. Dental tissue piezoelectric implants are typically stimulated by the natural movements of the mouth, such as chewing.

**Figure 11:**
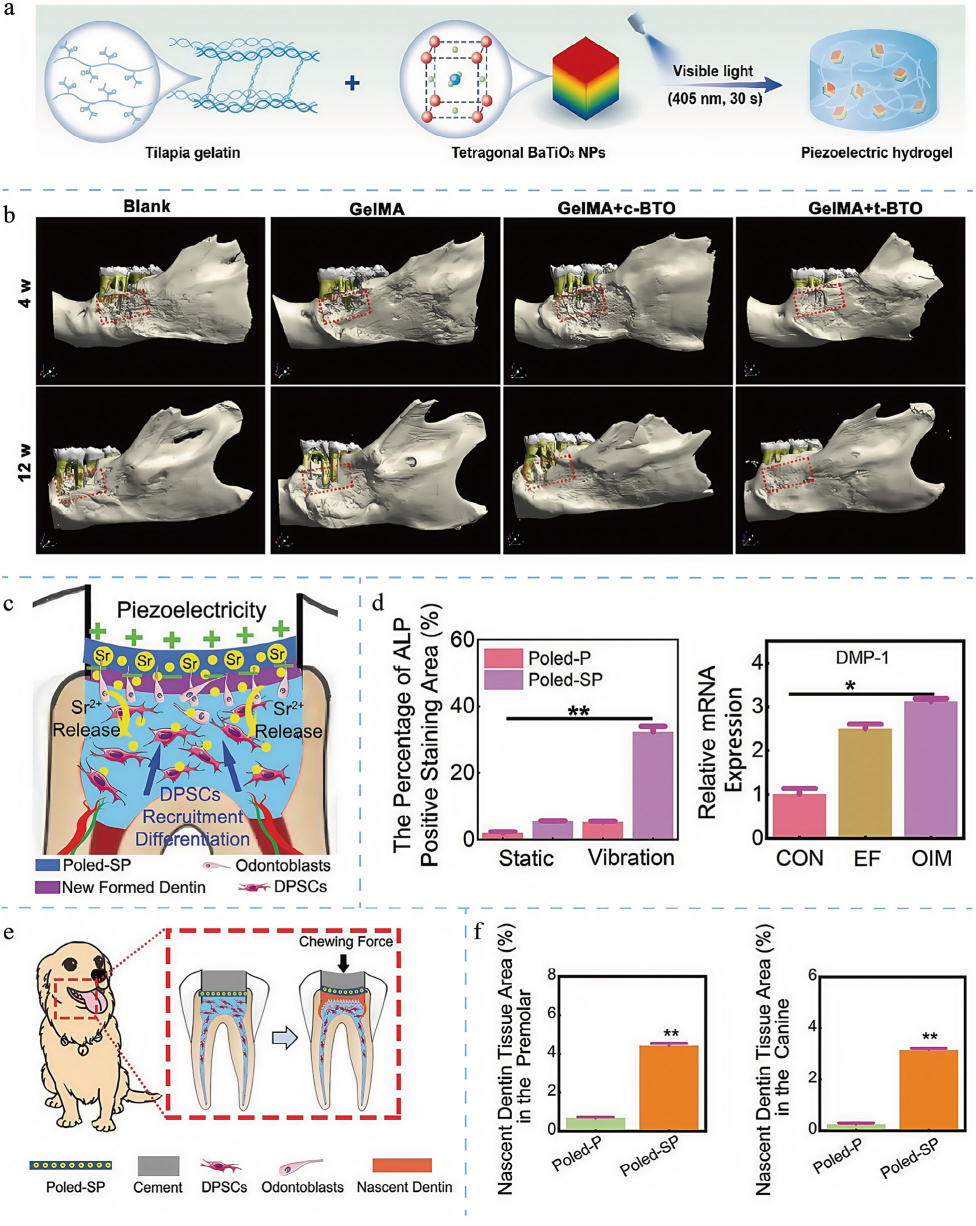
The cases for piezoelectric materials for dental tissue regeneration. a) Structure of the t‐BT/GelMA hydrogel.: b) Promotion of the repair of inflammatory periodontal defects. Reproduced with permission.^[^ ^183b^ ^]^ Copyright 2024, Ke Ai Publishing. c) Structure of the strontium‐containing P(VDF‐TrFE) film. d) Promotion of the differentiation of dental pulp stem cells. e,f) Promotion of dentin regeneration in vivo. Reproduced with permission.^[^ ^185^ ^]^ Copyright 2024, Wiley‐VCH.

Periodontitis is known to result in defects in the alveolar bone. Consequently, piezoelectric materials with anti‐inflammatory and osteogenic effects have the potential to be applied in the treatment of periodontitis. Studies by Roldan et al.^[^ ^183a^ ^]^ and Liu et al.^[^ ^183b^ ^]^ demonstrated the utility of BT‐loaded GelMA hydrogels in combating periodontitis, with the former relying on natural oral biomechanics and the latter relying on controlled ultrasonic stimulation to activate a piezoelectric response. The precision of ultrasound is pitted against the variability of mastication, emphasizing the need to balance utility and precision in clinical applications, especially in the unique mechanical environment of dental tissues. Roldan et al. prepared BT/GelMA hydrogels that were injected into inflammatory periodontal defects in mice and light‐cured for fixation.^[^ ^183a^ ^]^ Following a one‐month period, the mice exhibited notable improvements in periodontal pocket depth (0.55 mm to 0.2 mm), defective alveolar bone (75% bone volume change), and the inflammatory environment (73 blood vessels, close to the healthy group). Liu et al. prepared BT NPs with an excellent piezoresponsive tetragonal phase structure (t‐BT) by an annealing process and subsequently loaded them into GelMA hydrogels (**Figure** **11**a). The periodontal ligament stem cells (PDLSCs) were induced into an inflammatory state with lipopolysaccharide (LPS). Subsequently, the PDLSCs were co‐cultured with 50 µg/mL of t‐BT NPs and subjected to US stimulation (0.6 W cm^−2^, 1 MHz, 50% duty cycle, 60 s).^[^ ^183b^ ^]^ The results demonstrated that US‐activated piezoelectric t‐BT NPs elevated intracellular ATP levels and mitochondrial membrane potential. Additionally, the NPs up‐regulated and down‐regulated osteogenic and inflammation‐related genes, respectively, activated cytoskeletal reorganization by increasing intracellular calcium ion concentration and the expression of phosphorylated myosin light chain (p‐MLC). Furthermore, they promoted the expression of arginase‐1, a marker of M2 macrophages. In vivo tests in rats indicated that this composite hydrogel may facilitate alveolar bone regeneration in inflammatory environments (Figure 11b) by inducing cytoskeletal reorganization and macrophage M2 polarization.^[^ ^183b^ ^]^

Meanwhile, innovations in polarized PVDF films by Lai et al.^[^ ^184^ ^]^ introduced a new strategy for the simultaneous repair of bone and soft tissues, exploiting surface charge differences to direct cellular behavior—a key consideration for dual‐tissue interfaces in periodontal defects. Lai et al. prepared polarized PVDF films with osteogenic and gingival soft tissue regeneration ability on the positive and negative surfaces, respectively.^[^ ^184^ ^]^ Specifically, the negative side attracted calcium ions and BMPs (positively charged proteins) and up‐regulated osteogenic genes (Runx2, Ocn) about three‐fold under 14 days of co‐culture. And the positive side elevated roughly 1.5‐fold the number of human gingival fibroblasts (hGFs) migrating.

Complementing this finding, Li et al. integrated strontium into P(VDF‐TrFE) films, not only enhancing dentin regeneration but also highlighting the potential for multifunctional material design, which is particularly relevant for the mineral‐rich microenvironment of dentin.^[^ ^185^ ^]^ Li et al. prepared a strontium‐containing P(VDF‐TrFE) film (Figure 11c). In vitro, experiments have demonstrated that the expression of ALP and odonto‐differentiation genes (Runx2, DSPP, and DMP‐1) in dental pulp stem cells (DPSCs) is enhanced by direct voltage stimulation (Figure 11d). Consequently, piezoelectric stimulation has been shown to facilitate the repair of dentin by DPSCs. DPSCs located on strontium‐containing piezoelectric films under vibrational stimulation equivalent to 200 mV OV exhibited higher DSPP and DMP‐1 expression, as well as calcium mineral deposition, in comparison to DPSCs on simple P(VDF‐TrFE) films. The findings indicate that strontium ions may act in concert with DPSCs to facilitate tooth formation. Furthermore, pulpotomy was conducted on a Labrador dog with a piezoelectric membrane covering the pulp and dental cement applied to the membrane (Figure 11e). The results demonstrated that the piezoelectric membrane induced in vivo dentin regeneration, with significantly higher dentin tissue area both in the premolar and in the canine (Figure 11f).^[^ ^185^ ^]^

Overall, these efforts confirm the diversity of piezoelectric platforms; however, they also reveal key gaps. The durability and biocompatibility of these materials in long‐term use, especially in dynamic oral conditions, are still underestimated and require rigorous longitudinal studies. In addition, standardization of stimulation parameters—whether natural or external—and a deeper understanding of the cellular mechanisms that drive regeneration are critical. Given the unique immunological and biomechanical background of human dental tissues, the transition from animal models to human subjects remains a critical step. Therefore, future research should prioritize these aspects while exploring synergies with other regenerative agents and refining application techniques. By addressing these challenges, piezoelectric materials could redefine non‐surgical interventions in dentistry, providing minimally invasive and highly effective solutions for dental tissue regeneration.

### 6.7 Tendon Regeneration

**Figure 12:**
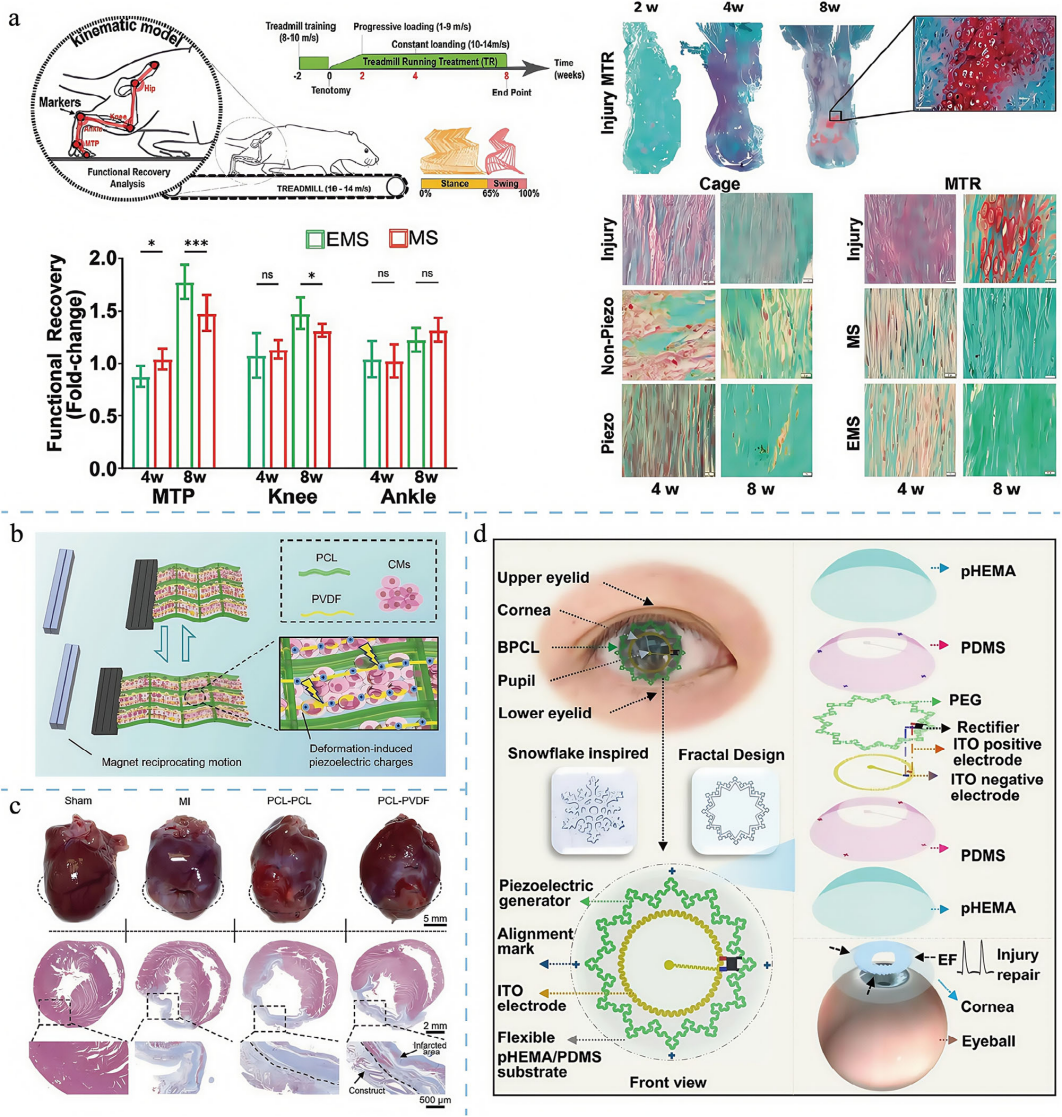
The cases for piezoelectric materials for tendon, myocardial, and corneal regeneration. a) Self‐powered piezoelectric tendon device promotes tendon recovery in vivo. Reproduced with permission.^[^ ^186a^ ^]^ Copyright 2021, Wiley‐VCH. b) Structure of the 3D printing of the cardiac serpentine scaffold. c) Improvement of myocardial infarction in vivo. Reproduced with permission.^[^ ^187^ ^]^ Copyright 2024, Wiley‐VCH. d) Structure of the Blink‐driven piezoelectric contact lens. Reproduced with permission.^[^ ^188^ ^]^ Copyright 2023, Nature.

Fernandez‐Yague et al. and Ge et al. presented two studies demonstrating the potential use of piezoelectric materials in tendon injury repair.^[^ ^186^ ^]^ Fernandez‐Yague et al. prepared P(VDF‐TrFE) scaffolds with aligned fibers with good piezoelectric (d_33_ = 36.5 pC N^−1^) and mechanical properties (EM = 62 MPa, TS = 31 MPa, elongation (EL) = 39%) by strain‐hardening and thermal annealing.^[^ ^186a^ ^]^ In vitro co‐culture with human tendon‐derived cells (hTDCs) demonstrated that periodic MS (4% dynamic strain at 0.5 Hz for 8 h d^−1^) of piezoelectric scaffolds resulted in more persistent up‐regulation of tendon‐related genes, such as SCX and TNMD expression, in hTDCs compared to non‐piezoelectric scaffolds. MS activates MSICs (e.g., PIEZO1/2 and TRP family) and simultaneously activates the BMP pathway for osteogenesis. The phenomenon of electromechanical synergy has the capacity to modulate the hyperactivation of the BMP pathway. The sophisticated modulation of the BMP pathway suggested that piezoelectric signals subtly fine‐tune differentiation pathways, potentially mitigating aberrant calcification—a frequent challenge in tendon injuries. A rat Achilles tendon injury model and treadmill exercise were employed to assess the in vivo effects of piezoelectric scaffolds (**Figure** **12**a). The findings demonstrated a reduction in calcification and an enhanced expression of tendon‐specific proteins (type I/III collagen) in the electromechanical group. This further substantiates the potential of piezoelectric signals to facilitate the regeneration of tendon tissue.^[^ ^186a^ ^]^

In a complementary effort, Ge et al. advanced this field by developing a piezoelectric elastomer, PETRR, with PDMS as a control, to sustain the tendon phenotype and promote functional recovery.^[^ ^186b^ ^]^ Their results demonstrated that piezoelectric signals facilitate the maintenance of the tendon phenotype in rat‐derived tendon cells in vitro (TNMD up, around 1.5‐fold) and the recovery of motor function in tendon‐injured rats (total travel distance up, around 1.3‐fold) in vivo. Additionally, PETRR was implanted in the rabbit Achilles tendon in combination with NFC modulus, which was able to monitor local temperature and determine movement patterns by monitoring voltage signals.^[^ ^186b^ ^]^ This dual role of the piezoelectric material as both a regenerative scaffold and a real‐time monitoring device marks a significant leap forward, offering the potential for personalized and adaptive regenerative therapies tailored to tendon injuries.

Collectively, these studies highlight the critical importance of aligning the mechanical and piezoelectric properties of scaffolds with the natural anisotropic structure of tendons to achieve organized and functional tissue regeneration. Yet, despite these advances, the precise mechanisms by which piezoelectric signals influence cellular behavior remain poorly understood, particularly in balancing tendonogenesis against osteogenic differentiation. Future research must focus on unraveling these mechanisms, refining stimulation protocols, and confirming the long‐term safety and efficacy of piezoelectric scaffolds for clinical translation.

### 6.8 Myocardial Regeneration

As previously stated, Jin et al. employed chest movements to activate piezoelectric/friction electric generators.^[^ ^173a^ ^]^ It can be seen, therefore, that the beating heart, as a natural source of motion and electromechanical coupling, can be combined with the electromechanical properties of piezoelectric materials, which have the potential to be used for in situ regeneration of myocardial tissue by harnessing their unique ability to convert the heart's mechanical motion into electrical signals that stimulate tissue repair. Two recent studies have provided evidence to support this hypothesis, in vitro and in vivo.^[^ ^187^, ^189^ ^]^ Doustvandi et al. cultured human umbilical vein endothelial cells (HUVECs) on PVDF/GO piezoelectric films (optimal GO concentration of 0.5 wt%) and observed that cell proliferation and adhesion were enhanced, which are critical drivers of tissue regeneration.^[^ ^189^ ^]^ Han et al. successfully printed PCL/PVDF composite scaffolds. In particular, one side of the scaffold comprised a serpentine PCL/PVDF elastic structure, while the other side was a PVDF/Fe_3_O_4_ magnetic structure (Figure 12b).^[^ ^187^ ^]^ In vitro assessment of the effect of piezoelectric signals released by the materials on rat cardiomyocytes utilized magnetic actuation. It demonstrated elevated levels of cardiac markers such as α‐actinin and CX43. The stent was further moved into a rat model of myocardial infarction (Figure 12c) with an in vivo OV of approximately 0.5‐1 mV. As a result, ejection fraction increased by 23% and fractional shortening by 14% in the PCL‐PVDF group compared to the MI group, infarct size decreased from 45.3 ± 2.05% to 22.7 ± 1.70%, CD68‐positive M1 macrophage area reduced from 13.88 ± 1.14% to 1.44 ± 0.27%, CD163‐positive M2 macrophage area rose to 24.6 ± 2.41%, microvessel density reached 74 ± 3.7 mm⁻^2^, and left ventricular internal diameter at end‐systole dropped from 7.54 ± 0.22 mm to 5.46 ± 0.40 mm after 28 days.^[^ ^187^ ^]^ These outcomes reflect notable improvements in cardiac markers and functional parameters, yet, nevertheless, the results did not demonstrate a statistically significant increase in survival in rats. This limitation underscores a broader challenge: although these materials adeptly replicate the heart's electromechanical milieu and foster cellular regeneration, their clinical therapeutic impact requires further refinement. Additionally, while incorporating GO boosts electrical conductivity and Fe_3_O_4_ imparts magnetic properties, the degree to which these enhancements integrate with myocardial tissue and support sustained benefits warrants deeper investigation. To confirm and optimize the role of piezoelectric materials in the treatment of heart attacks, larger samples of animals are needed, and future efforts should emphasize tailoring these materials to regenerate tissue effectively within the cardiovascular system while ensuring enduring compatibility with the heart's dynamic nature, paving the way for translating these encouraging findings into viable clinical therapies.

### 6.9 Corneal Regeneration

It is noteworthy that Yao et al. drew inspiration from the shape of a snowflake to prepare a flexible piezoelectric contact lens (BPCL) that utilizes electrical signals generated by blinking motions to repair corneal damage.^[^ ^188^ ^]^ In particular, the BPCL is comprised of a piezoelectric polypropylene electret film (generator), ITO/PET film (electrodes), and a micro‐rectifier (Figure 12d). The strategic use of these flexible and transparent materials ensures that the pupil area remains unobstructed while preserving the cornea's optical clarity—a vital attribute that sets it apart from other tissues and underscores the device's innovative edge in corneal regeneration. A moderate corneal alkali burn (grade II) model was developed to evaluate the in vivo effects of BPCL. To address the issue of low blink counts in mice and rabbits, an MS regimen was employed, comprising 4 KPa, 1 Hz, and 60 min, with a frequency of every 2 days. Yet, this dependence on external mechanical stimulation reveals a limitation: the current piezoelectric output may struggle to leverage the blink frequencies of humans, potentially hindering its practical application. The results demonstrated that the BPCL increased corneal clarity by 53% in mice and 50% in rabbits, with repair rates rising by 159% in mice and 52% in rabbits after 8 days, compared to controls.^[^ ^188^ ^]^ This marked disparity in species‐specific repair rates points to unresolved differences in corneal physiology, such as epithelial thickness or stromal responses, while mechanistically, the BPCL likely boosts epithelial migration through enhanced endogenous electric fields. Looking ahead, advancing this technology for clinical use will require not only enhancing the piezoelectric output with materials boasting higher voltage coefficients to eliminate reliance on MS, but also investigating bioactive coatings tailored to cornea‐specific pathways, ensuring both efficacy and comfort in applications where transparency remains paramount.

### 6.10 Frontier Practices from a Mechanism Perspective: Where Is the Road?

The previous sections outlined seven core pathways—intracellular Ca^2^⁺ levels, ion channels (VGCC, PIEZO1/2, and TRPV4), ECM/Integrin/FAK, Hippo (YAP/TAZ), Wnt signaling, TGF‐β/SMAD, and piezoelectric catalytic effects—that serve as the regenerative mechatronic synergism (Section 3). While these mechanisms have been extensively characterized, their practical applications in diverse tissues—bone, cartilage, peripheral nerves, central nervous system, wounds, dental tissues, tendons, myocardium, and cornea—show both progress and a great deal of untapped potential. This section assesses the extent to which these mechanisms are utilized in current applications and charts a course for advancing clinical translation.

Existing studies have shown that piezoelectric materials, in the form of scaffolds, hydrogels, and films, are involved in a subset of these mechanisms with varying success (Table 8). In bone regeneration, intracellular Ca^2^⁺ and ion channels, in particular VGCC and PIEZO1/2, are explicitly utilized, such as scaffolds to promote calcium influx and osteogenic gene expression via PI3K/AKT signaling.^[^ ^157^, ^161^, ^165^ ^]^ Cartilage repair is prominently characterized by the TGF‐β/SMAD pathway, and hydrogels promote cartilage differentiation through calcium signaling and ECM recruitment.^[^ ^169b,c^ ^]^ Peripheral nerve regeneration involves ion channels, possibly VGCC in Schwann cells, to drive axon growth,^[^ ^171^, ^173^ ^]^ whereas repair of the central nervous system, including the spinal cord and retina, involves Ca^2^⁺ signaling and PI3K/AKT pathways to promote neural differentiation and antioxidant effects.^[^ ^176^ ^]^ Wound healing uses the piezoelectric catalytic effect to generate ROS‐mediated antimicrobial effects alongside Ca^2^⁺‐driven tissue repair.^[^ ^178^, ^182^ ^]^ Dental regeneration promotes osteogenesis and dentin formation through cytoskeletal reorganization via ECM/Integrin/FAK and Ca^2^⁺ signaling.^[^ ^183^, ^185^ ^]^ Tendon repair activates PIEZO1/2 and TRPV4 ion channels and regulates BMP pathways,^[^ ^186a^ ^]^ whereas myocardial regeneration relies on Ca^2^⁺ influx to promote cardiomyocyte maturation.^[^ ^187^ ^]^ Corneal repair utilizes endogenous electric fields (possibly through ion channels) to promote epithelial cell migration.^[^ ^188^ ^]^ In these applications, ion channels and intracellular Ca^2^⁺ became generalized mechanisms, reflecting their role as primary responders to piezoelectric signals, whereas pathways such as TGF‐β/SMAD (cartilage, bone), ECM/Integrin/FAK (bone, teeth, wounds), and piezocatalytic (wounds) exhibited tissue specificity.

**Table 8:** Piezoelectric materials in tissue regeneration: applied mechanisms by tissue type.

Tissue	Mechanism	Refs.
Bone	intracellular Ca^2^⁺, ion channels (VGCC, PIEZO1/2)	[157, 161, 165]
Cartilage	TGF‐β/SMAD pathway	[169b,c]
Peripheral Nerves	ion channels (possibly VGCC in Schwann cells)	[171, 173]
Central Nervous System	Ca^2^⁺ signaling, PI3K/AKT pathways	[176]
Wounds	piezoelectric catalytic effect, Ca^2^⁺‐driven tissue repair	[178, 182]
Dental Tissues	ECM/Integrin/FAK, Ca^2^⁺ signaling	[183, 185]
Tendons	PIEZO1/2, TRPV4 ion channels, BMP pathways	[186a]
Myocardium	Ca^2^⁺ influx	[187]
Cornea	endogenous electric fields (possibly through ion channels)	[188]

Despite this progress, the field is still in the preclinical stage, and applications to mechanisms are limited by incomplete utilization and understanding. For example, while ion channels and Ca^2^⁺ signaling are widely activated, their downstream specificities‐such as how VGCC activation in bone differs from neural repair‐remain underexplored.^[^ ^161^, ^171^ ^]^ The TGF‐β/SMAD pathway, which is critical for cartilage, is not evident in the neural environment, suggesting a tissue‐specific impairment or oversight.^[^ ^169^, ^176^ ^]^ The Hippo pathway has rarely been explicitly cited despite its mechanosensitizing potential, possibly due to the focus on direct effectors such as Ca^2^⁺ rather than transcriptional regulators (Section 6). Similarly, Wnt signaling, which is critical for osteogenesis, lacks consistent application outside of bone,^[^ ^160a^ ^]^ while piezoelectric catalysis is largely limited to wound healing.^[^ ^178a^ ^]^ This uneven application suggests that while mechanisms are being translated into practice, their full scope has not yet been realized, and research tends to prioritize empirical results over mechanistic depth. Notably, the TGF‐β and wnt pathways lie at the translational frontier in Section 3.8, but are still little developed in animal experimental practice, which profoundly highlights where the field's potential lies.

**Figure 13:**
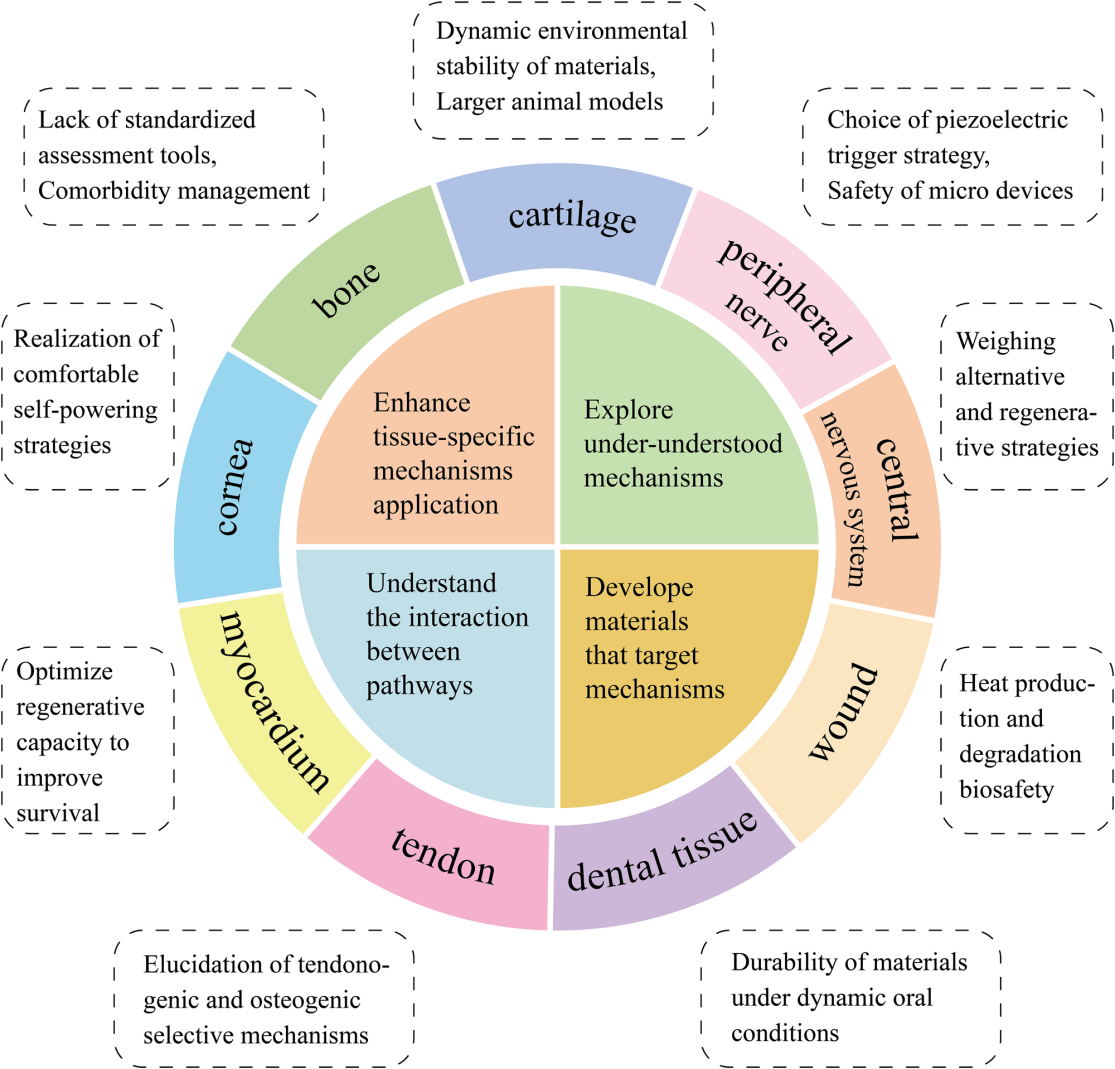
The mechanism‐driven strategy and current issues of the tissue application of piezoelectric materials. The four sections in the center are the four core elements of the mechanism‐driven strategy. The peripheral sections are the issues at this stage.

To bridge this gap and drive clinical translation, a mechanism‐driven strategy is one of the top pillars for future work (**Figure** **13**). First, enhancing tissue‐specific mechanism targeting is critical. In bone, expanding Wnt signaling could optimize osteoblast activity, building on current calcium‐driven successes,^[^ ^160^, ^161^ ^]^ and in nerve, elucidating the role of ion channels in axon guidance could refine conduit design.^[^ ^171c^ ^]^ Cartilage regeneration can benefit from fine‐tuning TGF‐β activation to balance chondrogenesis and avoid fibrosis.^[^ ^169b^ ^]^ Second, exploring underutilized pathways can provide untapped potential. The role of the Hippo pathway in mechanotransduction promotes the proliferation of mechanoresponsive tissues such as bone and cartilage, which warrants targeted studies (Section 3.4). Third, understanding the interactions between pathways‐for example, how PIEZO1/2‐triggered Ca^2^⁺ influx regulates TGF‐β or Wnt signaling‐may have synergistic effects, as in the network of mechanisms that we initially put together in Section 3. Fourth, it is crucial to develop materials that can selectively activate mechanisms. For example, designing piezoelectric coefficients according to scenarios that favor Ca^2^⁺ signaling in the myocardium and ROS generation in wounds can improve specificity.^[^ ^182^, ^187^ ^]^ Finally, the establishment of mechanistic biomarkers, such as Ca^2^⁺ flux for ion channel activity or SMAD phosphorylation for TGF‐β engagement, enables real‐time monitoring of efficacy, aligning preclinical insights with clinical needs.

In summary, while piezoelectric materials have begun to exploit key mechanisms across tissues such as ion channels and Ca^2^⁺ signaling, their clinical translation is still lagging due to the development of partial mechanisms and a lack of tailored applications. By prioritizing the optimization of tissue‐specific pathways, exploring neglected mechanisms such as Hippo, and engineering materials for precise mechanism control, the field can translate these pathways into clinically viable therapies. Such a mechanism‐driven strategy promises to elevate piezoelectric interventions from promising preclinical tools to transformative regenerative solutions.

## 7 Conclusion and Perspectives

Piezoelectric materials hold great promise for applications in tissue regeneration, especially the development of biodegradable natural piezoelectric polymers. Many tissues and components in living organisms are piezoelectric, such as bone, cartilage, tendon, cornea, sclera, skin, teeth, cochlear hair cells, blood vessels, and lungs. Theoretically, all life activities related to electromechanical signal conversion can be attempted to be modulated by introducing piezoelectric materials. Since electrical signals are inevitably present in tissues, all movements in the human body, from macroscopic joint movements, respiration, and heartbeat to microscopic hearing conduction, etc., can be attempted to be associated with piezoelectric materials to reprogram bioelectrical signals. Thus, ideas can be inspired. To improve the biomimetic properties of the materials, the piezoelectricity of human tissues can be re‐quantified using modern techniques, such as PFM and AFM, to obtain comprehensive and accurate piezoelectric data of human tissues.

To advance these materials toward clinical application, evaluation of piezoelectric materials is a top priority. Since both tumors and fibrosis are inherently uncontrollable tissue growth, many of the pathways associated with tissue regeneration are linked to tumor or fibrotic disease, requiring prudent development of piezoelectric materials for related functions. Enhanced precision targeting of disease will ensure safe clinical translation. In addition, excessive intensity of both ES and ultrasound may cause side effects in the tissue, so determining the optimal safety parameters is a prerequisite for the initiation of research. The mechanical characterization of the material should be adapted to the tissue and the mode of repair, e.g., repair of bone and cartilage focuses on the compressive strength of the material, and repair of nerves and tendons focuses on the material's tensile strength. Unilateral overflow of mechanical properties is not necessary, and attention should be paid to achieving all‐encompassing bionic mechanical properties. The piezoelectric evaluation should also be more precise, matching the tissue's movement or the stimulus's direction, such as vertical or shear stress. In addition, more comparative experiments are needed to illustrate the differences in the performance of materials in tissue regeneration, such as the increase in healing speed in similar animal models, to minimize the waste of research resources. Finally, degradability itself is an excellent material property, but there is a need to ensure that the tissue can be fully repaired before the material degrades completely to avoid the trauma of secondary implantation.

In addition, clinical translation requires deeper translation of mechanisms as well as standardized assessment metrics. We introduced seven pathways in the Mechanisms section, yet existing applications link few mechanisms, usually only the simplest Ca^2+^ signaling. Finding ways to modulate specific repair pathways in specific tissues with specific materials is the key to unlocking this black box of clinical translation. For a standardized process, all key parameters of the experimental procedure, including excitation parameters, piezoelectric output, and tissue regeneration effect data, must be uniformly defined and transparently disclosed to allow for direct comparisons and to determine the optimal material for a given application. For example, differences in current micro‐CT settings (resolution, threshold, region of interest) are somewhat of an impediment to reliable BV/TV comparisons. Standardizing these metrics and making inter‐material comparisons would take current research efforts out of the mud.

Furthermore, bridging the gap between animal models and human applications is critical. Human biomechanics, such as joint loading in bone and cartilage repair, differ significantly from animal models, and larger studies are needed to explain these differences. Similarly, neural applications must ensure that piezoelectric signals do not disrupt natural electrophysiologic activity, and translational studies incorporating patient‐specific factors, such as age and comorbidities, are needed to ensure safety and efficacy.

Finally, fostering collaboration between materials scientists, clinicians, and regulators is critical to overcoming translational barriers. Establishing interdisciplinary partnerships will streamline the materials development, clinical trial design, and regulatory approval processes, ensuring that innovative products such as biodegradable piezoelectric polymers and injectable hydrogels reach patients safely and effectively. And, harmonization of animal models is essential. Both different animal model types and inconsistent areas or lengths of tissue defects can lead to incomparable baselines.

Through these targeted advancements, piezoelectric materials have the potential to bridge fundamental regenerative mechanisms with practical, safe applications, moving closer to their clinical integration in tissue repair.

## Conflict of Interest

The authors declare no conflict of interest.

## Author Contributions

Y.Q. and V.S. conceived the idea. X.W. led the literature review and writing, with supervision provided by S.T.S. All authors reviewed and edited the paper before submission.
